# The claustrum contributes to working memory maintenance in mice

**DOI:** 10.1038/s41467-026-78363-6

**Published:** 2026-10-14

**Authors:** Anindya S. Bhattacharjee, Maxime Juventin, Chieko Huber, Burak Unsal, Jean-Rodolphe Renfer, Ivan Rodriguez, Alan Carleton

**Affiliations:** 1 Department of Basic Neurosciences, Faculty of Medicine, University of Geneva, 1 rue Michel-Servet, Geneva, Switzerland; 2 Department of Genetics and Evolution, Faculty of Sciences, University of Geneva, quai Ernest-Ansermet, Geneva, Switzerland

**Keywords:** Neural circuits, Working memory

## Abstract

Working memory (WM) enables the temporary maintenance and manipulation of sensory information to guide behavior, yet the neural circuits supporting this process remain incompletely defined. The claustrum is a highly interconnected brain structure positioned to integrate distributed cortical activity, but its contribution to WM has remained unclear. Here, we combined microendoscopic calcium imaging, chemogenetic and optogenetic manipulations in mice performing sensory-guided WM tasks to examine the role of the claustrum in WM. Claustrum neurons exhibited cue-evoked activity that persisted across delay periods and evolved dynamically over time, with population states retaining information about cue identity on single trials. Reducing claustrum excitability during encoding, delay maintenance, or target comparison similarly impaired WM performance across sensory modalities, without affecting sensory processing, motor output, arousal or inhibitory control. Together, these findings reveal an important causal contribution of the claustrum to WM maintenance.

## Abstract (web summary)

Neural mechanisms underlying working memory are not fully understood. Here authors show that in mice, the claustrum helps keep memories alive: its neurons sustain information during working memory, while disrupting claustrum activity impairs memory across sensory modalities.

## Introduction

Working memory (WM) is a core cognitive process that enables temporary maintenance and manipulation of information necessary for reasoning, problem-solving, language comprehension, and decision-making^1,2^. Deficits in WM are prominent in neurological and psychiatric disorders, including ADHD and schizophrenia^3^. Despite decades of research, the neural mechanisms supporting WM remain incompletely understood. Current models emphasize that WM emerges from distributed interactions across cortical and subcortical circuits rather than being localized to a single region^4–6^.

The prefrontal cortex (PFC), particularly the dorsolateral PFC in primates and its equivalent, the medial PFC in rodents^7^, has long been considered a central component of WM networks^4,8,9^. Early studies linked WM to sustained delay-period activity in PFC pyramidal neurons^10–16^. However, as recording technology advanced and enabled the monitoring of larger groups of neurons, transient rather than sustained responses became more commonly observed, with population activity sometimes disappearing during the delay only to reemerge later^9,17–20^. These findings led to theories that information might be maintained not solely through PFC firing activity but also through short-term plasticity in synaptic connections during silent intervals^21,22^ or through a combination of both mechanisms^23^.

Although contributing to WM, the PFC is not the sole region involved, and evidence suggests it may play a more prominent role during WM task learning, rather than in information storage after the task is mastered^15^. Research in humans and other mammals indicates that WM relies on a distributed network, encompassing sensory cortices^24–27^, associative areas within the frontal^10–16,28,29^ and parietal^30,31^ lobes, the basal ganglia^32,33^, thalamus^34–36^, and hippocampus^37–39^. Within this distributed architecture, different regions are thought to contribute complementary functions, including encoding, maintenance, transformation, and readout of task-relevant information. However, how these distributed representations are sustained and transformed over time remains incompletely understood.

The claustrum (CLA), a subcortical structure located between the striatum and insular cortex, is well positioned to contribute to these processes. It consists primarily of glutamatergic projection neurons and exhibits widespread, reciprocal connectivity with nearly all cortical regions^40–45^. Through these connections, the CLA can influence cortical activity in a context-dependent manner, as demonstrated by optogenetic studies showing region- and cell type–specific modulation of cortical circuits^44,46,47^. These anatomical and functional features suggest that the CLA may contribute to the maintenance of task-relevant activity within distributed WM networks.

Here, we combined microendoscopic calcium imaging with causal manipulations in mice performing sensory-guided WM tasks to examine how CLA activity contributes to WM representations and behavior.

## Results

### Working memory performance across distinct sensory modalities in mice

**Fig. 1:**
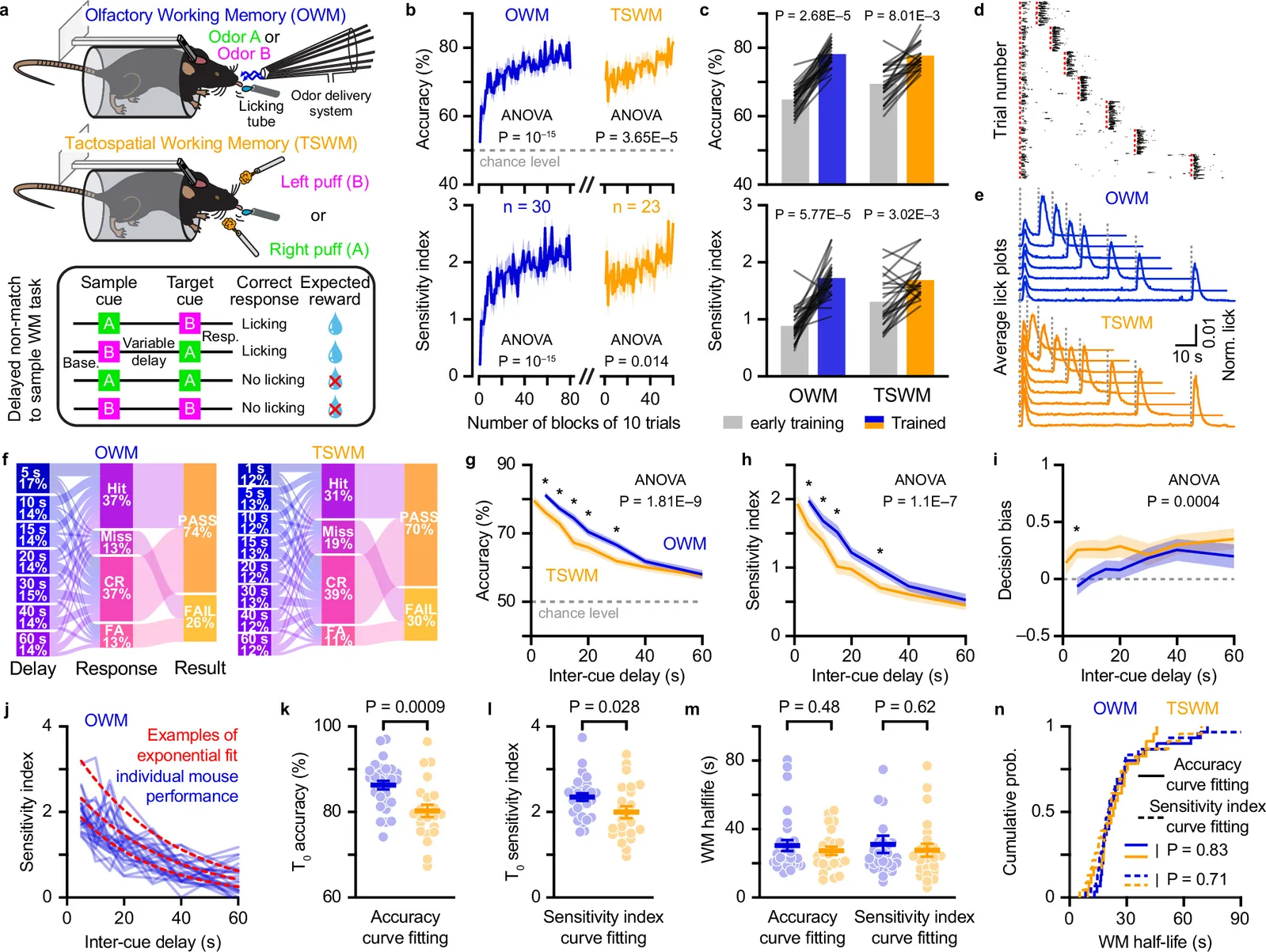
Working memory performance across distinct sensory modalities in mice. **a** Schematic representation of the behavioral paradigm designed to evaluate olfactory and tactospatial WM (OWM and TSWM, respectively) in head-restrained mice. Both sample and target cues were presented for one second. Base.: baseline, Resp.: response window. **b** Learning curves for OWM and TSWM tasks (fixed inter-cue delay of 5 and 1 s, respectively; n = 30 and 23 mice, respectively; one-way repeated measures ANOVA). **c** Change in performance between the first and the last 100 trials of the training phase shown in b for each mouse (black lines; n = 30 and 23 mice for OWM and TSWM, respectively; 2-sided paired t-test) and the mean population performance (colored bars). **d** Example lick raster from a single mouse performing the OWM task (trials sorted by delay duration). Dotted lines indicate cue onset. **e** Peri-stimulus time histogram showing normalized lick initiation counts averaged across all mice during OWM and TSWM sessions (*n* = 30 and 23 mice, respectively). **f** Sankey plots summarizing the distribution of trial outcomes across mice for different delay durations in both tasks. CR: correct rejections, FA: false alarms. **g**–**i** WM performances plotted as a function of the inter-cue delay duration (2WRM ANOVA, post-hoc Fisher’s test *: P < 0.05). **j** Variability of WM performance across individual animals shown for sensitivity index computed during the OWM task. Examples of exponential fit (used to calculate the amplitude at 0 delay and the half-life) of a few individual performance curves are illustrated (red dashed lines). **k**–**m** Comparison of WM performance amplitude at 0 delay and half-life for OWM and TSWM tasks (*n* = 30 and 23 mice, respectively; 2-sided unpaired t-test). **n** Cumulative probability distribution of half-lives for OWM and TSWM tasks (*n* = 30 and 23 mice, respectively; Kolmogorov-Smirnov test). Data in **b, e, g-i, k-m** are presented as mean ± SEM. See Supplementary Table 1 for detailed statistics. Source data are provided as Source Data file.

We first aimed to compare WM performance across two different sensory modalities, olfactory and tactospatial information, in head-restrained mice (Fig. 1a). To achieve this goal, we employed a go/no-go paradigm, which entailed presenting sequences of either two odorants or two tactile stimuli as sensory cues. A sample cue was presented for one second and was required to be retained during a delay period. Subsequently, the mouse encountered a target cue, which could either match the sample stimulus (match trials) or be a different cue (non-match trials). To earn a reward, mice were trained to lick a reward tube within a defined response window, specifically for non-match trials, thus following a delayed non-match-to-sample design. Successful trials demonstrated the ability of the animals to transiently store information in their WM for future comparison, as neither the first nor the second cue alone could reliably predict the reward outcome. Pairs of cues for olfactory WM (OWM) and tactospatial WM (TSWM) tasks were defined using two easily discriminated odorants^48^ and an airflow stimulation of the left or right whisker pad, respectively (Fig. 1a). We computed the percentage of correct responses, or accuracy, specifically referring to non-match trials in which licking during the response window exceeded that during the pre-response window, and match trials in which licking during the response window was absent or lower than during the pre-response window (see methods and Supplementary fig. 1a, b). Trials not meeting these criteria were classified as errors. Furthermore, we distinguished between false alarms, correct rejections, hits, and misses to calculate the accuracy, sensitivity index, and decision bias. The mice were initially trained to perform an OWM task with a fixed inter-cue delay of five seconds. They rapidly learned the task and started performing with >70% accuracy in blocks of 10 trials within ~400 trials (Fig. 1b, c). Subsequently, mice performed daily sessions in which inter-cue delays were varied across the session (5–60 s). Each delay was presented in short blocks of 2–4 consecutive trials, with block order randomized. Match and non-match trials were independently randomized and presented in equal proportions. Next, the mice were trained to perform the TSWM task with a fixed inter-cue delay of one second. The majority of mice (23 out of 25; the remaining two mice dropped out of WM tasks as they stopped engaging in the tasks) swiftly adjusted to the new sensory dimension and attained high-performance levels (Fig. 1b, c). Finally, the animals underwent sessions during which the tactospatial inter-cue delay was randomly varied across blocks of trials.

During multi-delay sessions, mice reliably initiated licking during the sample cue, independent of cue type (Fig. 1d, e and Supplementary fig. 1a). This behavior, which arose from the pretraining requirement to obtain a small water reward following cue presentation, was neither discouraged nor punished during later sessions, as it could provide a useful measure of arousal and engagement. While some licking occurred during cue presentation, mice rapidly learned to suppress licking during the inter-cue delay period before responding to the target cue. This behavior was consistent across all delays, indicating sustained task engagement and intact motor control, and demonstrating that mice could withhold licking for up to 60 s before responding to the target cue (Fig. 1d, e and Supplementary fig. 1a, b). To classify behavioral responses, licking during the response window was compared with a pre-response baseline spanning the entire delay period (see Methods). By keeping the pre-response baseline proportional to the delay duration, we normalized it to the time available for spontaneous, non-specific licking, thereby reducing the likelihood that delay-dependent increases in such licking would be misclassified as Hits or False Alarms. As expected for a WM task, increasing delay duration imposed a greater cognitive load, resulting in a progressive decline in performance (Fig. 1f-h). This decline was characterized by increases in both false alarms and misses (Supplementary fig. 1c), consistent with reduced discriminability between match and non-match stimuli. Importantly, the same pattern was observed when trial outcomes were reclassified post hoc using a fixed 5 s baseline composed of the first 2.5 s following sample onset and the final 2.5 s immediately preceding the response window (Supplementary fig. 1d). These results demonstrate that the delay-dependent decline in performance is not attributable to the duration of the pre-response baseline used for trial classification. Furthermore, increasing the delay shifted mice toward a more conservative response criterion, as reflected by an increase in decision bias (Fig. 1i). At both population average and individual levels of analysis, WM performance for both tasks showed an exponential decline with increasing inter-cue delay (Fig. 1g,h,j). Although performance was consistently slightly higher for the OWM task than for the TSWM task (Fig. 1k,l), memory half-life remained independent of the sensory dimension used ( ~ 20 and 30 seconds for the median and mean, respectively; Fig. 1m, n). Despite some variability in performance across individual sessions and mice, population-level performance remained stable across consecutive sessions and across the two different odor pairs tested (Supplementary fig. 1e-h). Similar trends were observed in mice that performed both OWM and TSWM tasks (Supplementary fig. 1i). In summary, these findings demonstrate that WM performance can be reliably assessed across multiple sensory dimensions in mice.

### Sensory cues evoke persistent, non-motor CLA population activity throughout WM delays

**Fig. 2:**
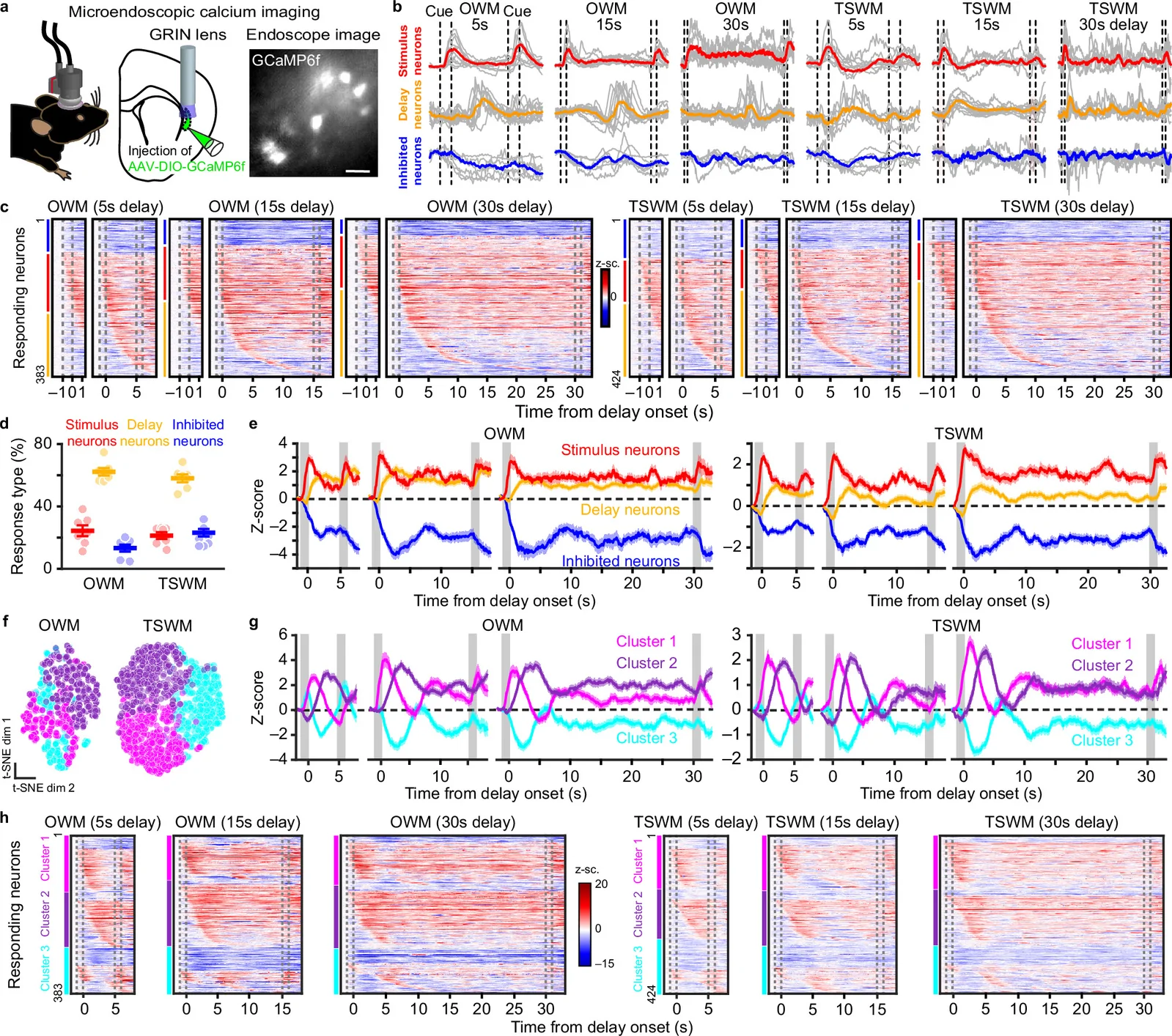
Persistence of claustrum neuron responses after sensory cue presentation. **a** Schema of the calcium imaging procedure. Image of CLA neurons expressing the genetically encoded calcium indicator GCaMP6f captured through the GRIN lens of the endoscope camera (scale bar: 50 µm). **b** Examples of individual neuron responses during OWM and TSWM tasks. Grey and colored traces represent individual and averaged correct trials for different response types, respectively. Dashed black lines mark cue application periods. **c** Pseudo-colored heat maps presenting trial-averaged responses from correct trials for all stimulus, delay, and inhibited neurons (distribution indicated by the color lines on the left, same code as in **b**). Neurons were pooled from 7 mice and sorted by response type and latency (z-score min: –15, max: 20 and 15 for OWM and TSWM maps, respectively). For each delay duration, an expanded zoom including the first second of the delay is shown. Dashed grey lines indicate cue applications. **d** Quantification of the different response types recorded in 7 different mice (each data point represents the mean value computed across different delays for each animal; data presented as mean ± SEM). **e** Average population calcium activity (data presented as mean ± SD). Grey bars represent cue applications. **f** t-SNE projection of neurons clustered using k-means clustering (all delay durations used). **g** Average population calcium activity of neurons assigned to each cluster (mean ± SD). Grey bars indicate the timing of cue presentations. **h** Pseudo-colored heatmaps showing trial-averaged responses from correct trials for the three clusters identified using k-means clustering (Cluster identity is indicated by the color bars on the left, matching the color scheme used in **f**). Neurons were pooled from 7 mice and sorted by response latency within each cluster. Dashed grey lines indicate cue applications. Source data are provided as Source Data file.

We next monitored the activity of genetically identified CLA neurons using microendoscopic calcium imaging in mice performing the WM tasks described above (Fig. 2a and Supplementary fig. 2a). To achieve this, we virally expressed the genetically encoded calcium indicator GCaMP6f in CLA projection neurons expressing either the vesicular glutamate transporter 2 (VGLUT2; Fig. 2) or *Smim32* (Supplementary fig. 2), two partially overlapping genetic markers of CLA projection neurons^44,45,49^. Changes in calcium activity were tracked as an indicator of modulation in firing rates. To mitigate the risk of phototoxicity, imaging was restricted to trials with delays of 5, 15, and 30 seconds. To quantify CLA activity during the WM tasks, we first averaged the z-scored calcium signals across all correct trials, irrespective of the cue used. CLA neurons reliably altered their activity across trials during the WM tasks and could be categorized into three groups based on their temporal response profiles during the trial structure (Fig. 2b-e and Supplementary fig. 2b, c). The first type, referred to as stimulus neurons, displayed increased activity immediately upon presentation of the sample cue. These neurons exhibited complex response patterns during the delay period, with some possible periods of inhibition and/or excitation. The second group, termed delay neurons, displayed heightened calcium activity during the inter-cue delay, consistently peaking at specific time intervals across trials. Finally, a third group exhibited inhibition during extensive portions of the trial. Notably, these distinct response types were preserved across sensory modalities, delay durations, animals, and Cre-driver lines (Fig. 2b-d and Supplementary fig. 2b,c). On average, ~25% of the cells were categorized as stimulus neurons, ~15% as inhibited neurons and ~60% as delay neurons (Fig. 2d).

To further investigate organizational structure within the CLA population beyond response latency, and without relying on threshold-based classifications, we applied unsupervised k-means clustering. Across experiments, the elbow method consistently identified three neuronal clusters as the optimal solution, which were visualized in t-SNE space (Fig. 2f). This approach revealed activity patterns initiated by the sample cue that largely recapitulated the response motifs identified by the latency-based classification, while highlighting enhanced fluctuations in calcium activity during the delay period (Fig. 2g,h and Supplementary fig. 2d–f). Moreover, response dynamics differed across clusters, suggesting that cue information might be encoded through temporally evolving population activity rather than static response states. Consistent across classification methods, population-averaged activity remained sustained, though dynamically modulated, throughout the delay period until target cue presentation for all inter-cue durations (Fig. 2e,g and Supplementary fig. 2c,f).

**Fig. 3:**
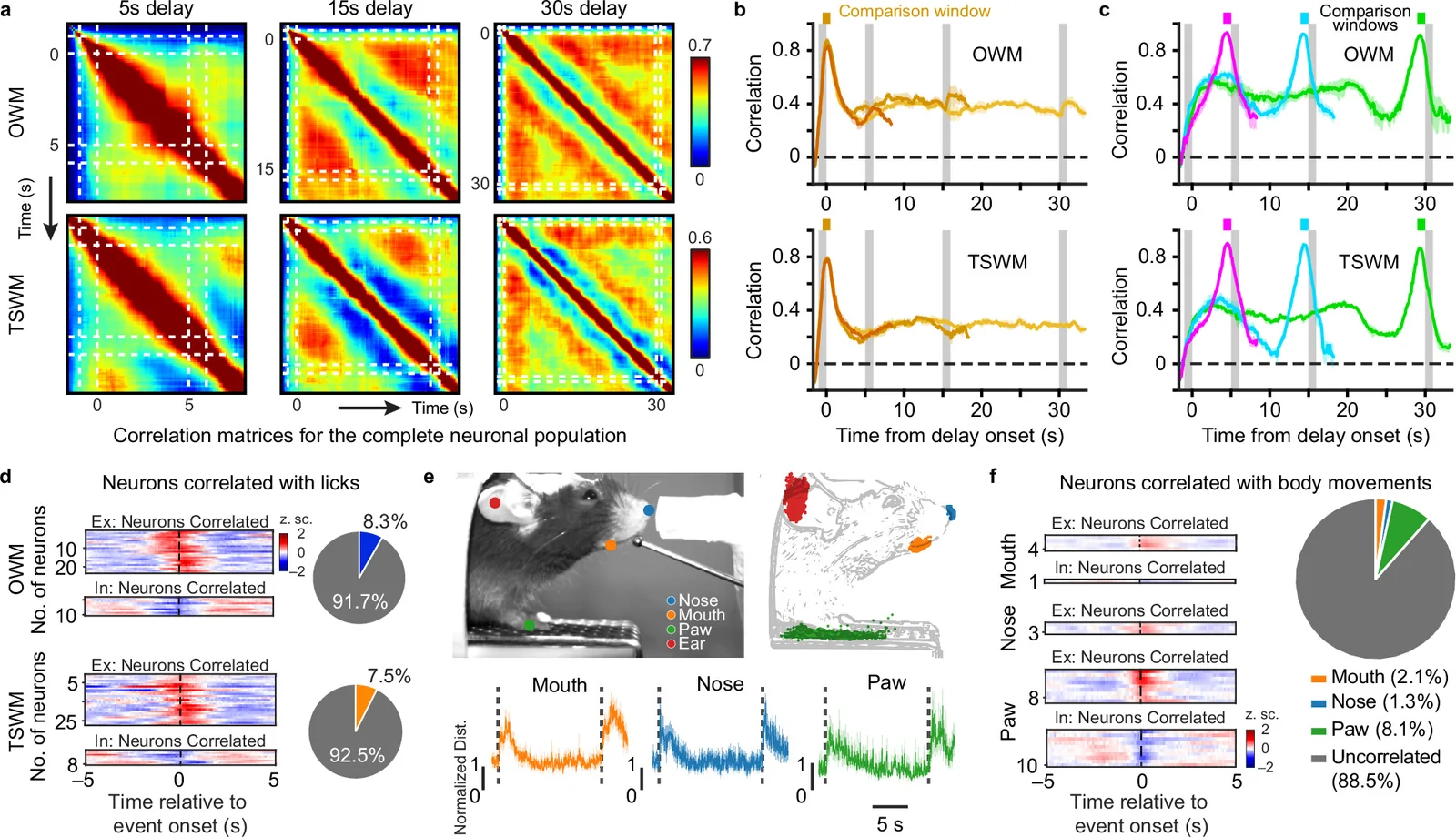
CLA population dynamics and its correlation with body movements. **a** Correlation matrices showing all possible correlations computed between pairs of population activity vectors at different time points (time bin = 100 ms). Dashed white lines represent cue presentation. **b**, **c** Time course of ensemble correlations for different trial structures (sample A–target B, sample A–target A, sample B–target A, sample B–target B; mean ± SD), plotted for various delay durations in the two WM tasks. Comparison windows (1 s) are color-coded above the graphs; grey bars denote cue presentation. **d** Pseudo-colored heatmaps of neurons significantly correlated with lick onset, grouped by excitation or inhibition. The pie chart indicates the fraction of lick-correlated neurons relative to the total recorded population for OWM and TSWM tasks (blue and orange, *n* = 383 and 424 neurons, respectively). **e** Video snapshot showing body-part tracking during CLA imaging in a WM task. Right panel: representative coordinates for mouth, nose, and right paw. Below: normalized movement traces (mean ± SEM) averaged across mice for all trials with a 15 s inter-cue delay. **f** Pseudo-colored heatmaps of neurons correlated with tracked body movements, aligned to movement onset and grouped by excitatory or inhibitory responses. No significant correlations were detected with ear movements. The pie chart shows the proportion of neurons correlated with movements of each body part relative to the total recorded population in the OWM task (*n* = 228 neurons). Source data are provided as Source Data file.

To further characterize the structure of these dynamics across different delay durations and sensory modalities, we constructed high-dimensional population vectors, in which each dimension corresponded to an individual responsive neuron pooled across animals. For each dimension, we computed the mean z-scored calcium activity averaged across all correct trials for each task–delay condition in 100 ms time bins. This framework enabled us to represent the temporal evolution of CLA population activity as a vector time series for each WM task and delay duration. To quantify changes in population activity over time, we calculated the similarity between population vectors using Pearson correlation coefficients. Correlation matrices revealed that presentation of the sample cue evoked a population response that persisted throughout the delay period, although correlation strength fluctuated over time, reflecting dynamic reconfiguration of the active neuronal ensemble (Fig. 3a and Supplementary fig. 2g). When all vectors were compared to those at the peak stimulus-evoked response, correlations gradually declined during the delay before reaching a plateau, yet remained consistently above baseline, indicating partial maintenance of cue-evoked population states (Fig. 3b). Similarly, aligning comparison windows to the end of each delay period revealed correlations that again exceeded baseline during cue presentation, but with more pronounced temporal fluctuations, suggesting increased ensemble reorganization over time (Fig. 3c). Collectively, these analyses demonstrate that, across delay durations and sensory domains, sensory cues elicited changes in CLA neuron activity that persisted throughout the entire delay period, albeit with evolving population dynamics over time.

Previous studies have shown that CLA neurons can be modulated by movement, particularly licking behavior during task engagement^50^. To determine whether the population dynamics observed in CLA neurons during WM tasks were driven by licking during the inter-cue delay period, we identified neurons whose activity was temporally correlated with lick events. Depending on the CLA neuronal population imaged, approximately 3% of *Smim32*⁺ and 8% of *Vglut2*⁺ neurons exhibited lick-correlated activity during WM tasks (Fig. 3d and Supplementary fig. 2h). Most of these neurons showed increased activity preceding lick onset, whereas a smaller subset displayed inhibitory responses prior to licking. These findings indicate that although a minor fraction of CLA neurons tracks licking behavior, the predominant population dynamics observed during WM tasks are unlikely to be explained by licking alone. To further assess whether CLA activity reflected other forms of movement in head-fixed mice, we tracked the positions of the nose, mouth, and forepaw during imaging sessions (Fig. 3e). Cross-correlation analyses revealed that only a small proportion of neurons were modulated by these movements (2.1% for nose, 1.3% for mouth, and 8.1% for paw; Fig. 3f). Together, these results demonstrate that sensory cues evoke persistent CLA population activity throughout WM delays that is largely independent of overt motor behavior, indicating that the observed dynamics are not merely a byproduct of gross movements.

### Claustrum ensembles maintain cue information during working memory

**Fig. 4:**
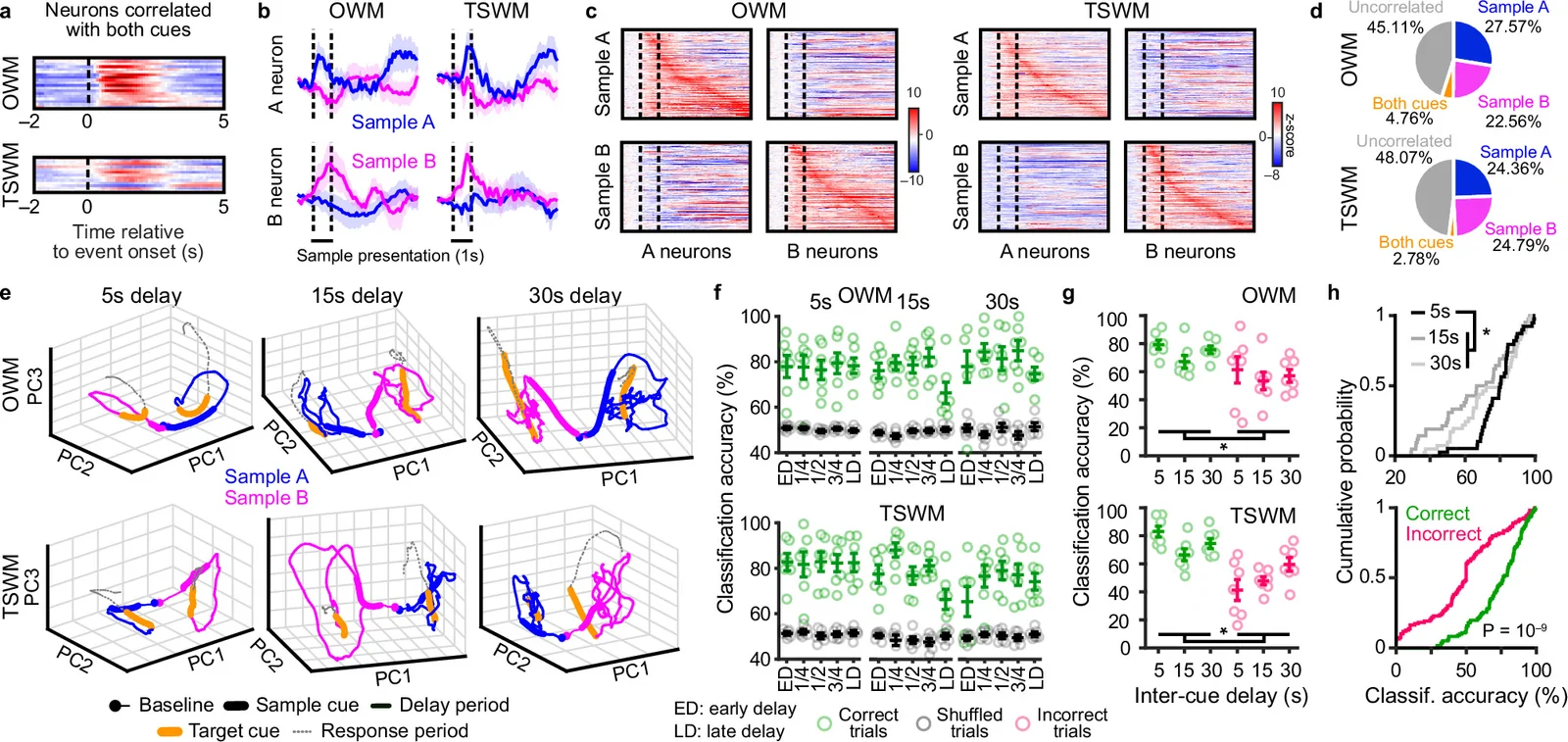
Claustrum ensembles maintain cue information during working memory. **a** Heatmaps of neurons significantly correlated with both sample cues (n = 19 & 13 for OWM & TSWM, respectively). **b** Averaged calcium traces from four cue-selective stimulus neurons (mean ± SD). **c** Heatmaps of trial-averaged responses from all cue-selective stimulus neurons, sorted by peak latency for the preferred cue. Neuron order was preserved for the non-preferred cue (n = 165, 150, 187, 191 neurons for samples A & B in OWM and TSWM tasks, respectively). **d** Pie charts showing proportions of neurons correlated with both sample cues or only one, relative to the total recorded population in OWM and TSWM tasks (n = 383 and 424 neurons, respectively). **e** Population activity trajectories for the averaged correct trials with Sample A or B, plotted in PCA space. **f** Classification accuracy for decoding sample identity at regularly spaced time points across the delay period: early delay (ED; 1 s after sample offset), one-quarter delay, mid-delay, three-quarter delay, and late delay (LD; 1 s before target onset). Classification accuracy is shown for correct trials and shuffled controls in both tasks (*n* = 7 mice, RM ANOVA: shuffling effect F_(1,40)_ = 350.9 P = 1.0E–15 and F_(1,40)_ = 353.2 P = 1.0E–15, effect across time periods: F_(4,30)_ = 1.4 P = 0.24 and F_(4,30)_ = 1.396 P = 0.26, for OWM and TSWM, respectively). **g** Classification accuracy for decoding sample identity during late delay periods in correct and incorrect trials for both tasks (*n* = 7 mice, correct vs. incorrect trials: RM ANOVA F_(1,6)_ = 15.48, P = 0.0077 and F_(1,6)_ = 27.71, P = 0.0019 for OWM and TSWM tasks, respectively). **h** Cumulative probability distributions of sample cue classification accuracy. Top graph: correct trials for different delays (n = 42 animal/session/task triplets, Kolmogorov-Smirnov test P = 3E-4, 0.009 and 0.29 for 5 s vs. 15 s, 5 s vs. 30 s, and 15 s vs. 30 s, respectively). Bottom graph: comparison between correct and incorrect trials (n = 126 & 124 animal/delay/session/task quadruplets for correct & incorrect trials, respectively). Data are presented as mean ± SEM. See supplementary Table 1 for detailed statistics. Source data are provided as Source Data file.

We next examined whether the identity of the sample cue could be decoded from CLA population activity. We first quantified the proportion of neurons exhibiting cue-selective responses in each WM task. We found that a small fraction of CLA neurons responded non-selectively to both sample cues (4.8% and 2.8% during OWM and TSWM tasks, respectively; Fig. 4a). These broadly tuned responses may reflect overlapping response profiles or, alternatively, the presence of a small subset of CLA neurons that are recruited by general task demands or stimulus presentation rather than encoding specific cue identity. In contrast, a substantially larger proportion of neurons exhibited a selective increase in calcium activity during the presentation of one cue but not the other (Fig. 4b). Approximately one quarter of neurons responded selectively to each cue in both OWM and TSWM tasks (Fig. 4c,d). Intriguingly, presentation of the alternative cue could elicit either inhibitory responses or delayed increases in calcium activity during the delay period (Fig. 4b,c). This observation suggests that the three response groups identified in Fig. 2b–e likely reflect functional tuning to different cue identities rather than representing fixed, distinct neuronal subpopulations^51,52^.

We next examined the dynamics of cue-evoked activity across the entire recorded CLA population for each sensory modality. Using dimensionality reduction, we projected population activity during averaged correct trials into a low-dimensional neural space defined by the first three principal components, which captured the greatest variance. Following cue presentation, population activity departed from baseline and evolved along cue-specific trajectories that remained distinct for the two cues, not only during cue presentation but throughout the delay period (Fig. 4e). This separation was consistently observed across delay durations and sensory modalities. As anticipated from the correlation analyses (Fig. 3a–c), population representations evolved dynamically over the delay period while remaining sufficiently distinct to potentially preserve information about cue identity (Fig. 4e).

We next assessed how reliably cue identity was maintained by the CLA population on individual trials. To this end, we applied a decoding approach to predict cue identity across animals, delays, and tasks. Specifically, we implemented a bootstrapping procedure to randomly sample correct trials and trained a classifier to determine whether CLA population activity could discriminate between sample cues during the WM task. Classifier performance was evaluated at regularly spaced time points across the delay period: early delay (ED; the first second after sample offset), one-quarter delay (1/4), mid-delay (1/2), three-quarter delay (3/4), and late delay (LD; the final second before target presentation). Classifier performance across the delay periods was comparable and consistently exceeded chance levels (i.e., relative to shuffled data), indicating that cue identity was retained throughout the delay (Fig. 4f, g). Furthermore, decoding accuracy declined at longer delays (Fig. 4h). Notably, although fewer incorrect trials were available, classifiers trained on correct trials performed poorly when applied to incorrect trials, with prediction accuracy markedly reduced across delays and tasks (Fig. 4g, h and Supplementary fig. 2i, j). Together, these results indicate that cue identity is robustly decodable at the single-trial level and that this information is broadly represented across the CLA population during the delay period. These findings suggest that the CLA not only encodes cue-specific information during stimulus presentation but also maintains this information across the delay, with neural decoding performance paralleling behavior.

### Chemo- and optogenetic inhibition of CLA neurons disrupts working memory

**Fig. 5:**
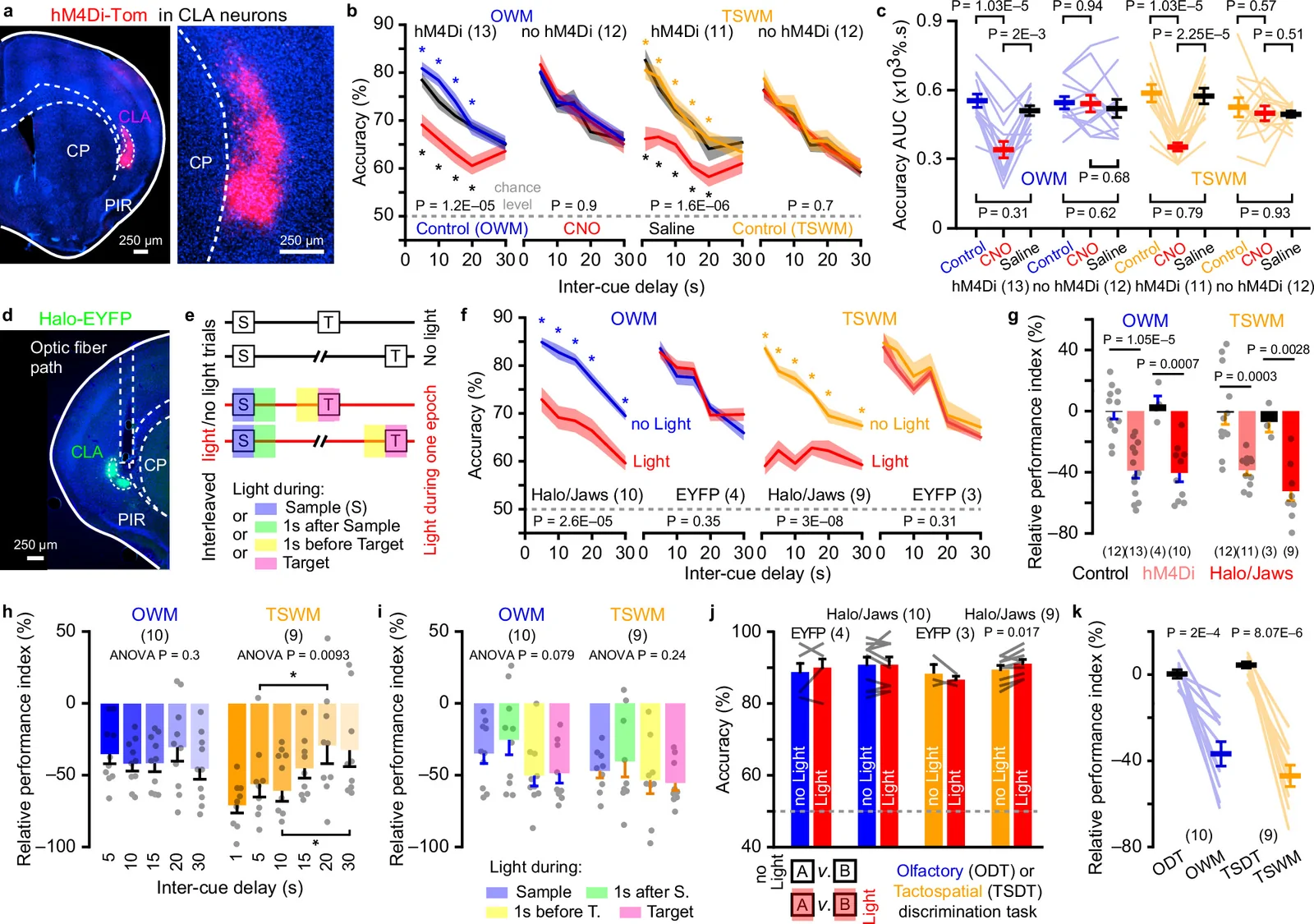
Chemogenetic and optogenetic inhibition of CLA neurons disrupt working memory. **a** Photograph of virally targeted CLA neurons. CP: caudate putamen, PIR: piriform cortex. **b** WM performances as a function of inter-cue delay duration for mice expressing hM4Di in the CLA and control mice without hM4Di. Control (no injection), CNO, and saline sessions were performed sequentially (control v. CNO: RM ANOVA; post-hoc Fisher’s test *P < 0.05: Control v. CNO and Saline v. CNO shown in blue/orange and black, respectively). **c** Quantification of global WM performance during the first 30 seconds using the Area Under the Curve (AUC) of curves shown in **b**. **d** CLA neurons expressing the inhibitory opsin eNpHR3.0-EYFP. **e** WM sessions with interleaved light/no-light trials across delays. In light trials, CLA neurons were inhibited during one of four epochs (one epoch per session; see Supplementary fig. 3e). **f** WM performance averaged across stimulation epochs as a function of delay for mice expressing Halo/Jaws or EYFP in the CLA. Performance for light and no-light trials is compared within each group (RM ANOVA; post-hoc Fisher’s test comparisons *: P < 0.05). **g** Relative performance index (relative to control performance, i.e. no CNO or no light conditions) following chemogenetic or optogenetic CLA inhibition in OWM and TSWM tasks shown in b & f (comparison between control and experimental groups: 2-sided unpaired t-test *P < 0.05). h, Effect of optogenetic CLA inhibition on WM performance across delays (RM one-way ANOVA and Tukey’s post-hoc test*: P < 0.05). **i** Effect of CLA inhibition on WM during different light epochs for both tasks (RM ANOVA across epochs). **j** Performance in olfactory discrimination task (ODT) and tactospatial discrimination task (TSDT) with interleaved light/no-light trials. (2-sided Paired t-tests). **k** Comparison of photoinhibition effects on sensory discrimination tasks v. WM tasks with light during the sample period (2-sided Paired t-tests). The number of animals is indicated in parentheses in each figure panel. Data are presented as mean ± SEM. See Supplementary Table 1 for detailed statistics. Source data are provided as Source Data file.

We next asked whether sensory information encoded within CLA population activity is functionally required for WM performance. To test this, we selectively reduced CLA neuronal excitability using a chemogenetic approach (Fig. 5 and Supplementary fig. 3). Specifically, we expressed the inhibitory designer receptor exclusively activated by designer drugs (DREADD) hM4Di^53^ in CLA neurons of adult mice (Fig. 5a). Chemogenetic suppression of CLA excitability via clozapine-N-oxide (CNO) administration impaired WM performance regardless of the sensory dimension probed (Fig. 5b and Supplementary fig. 3a). This effect was transient, with performance returning to baseline following saline injection on subsequent days. Importantly, the impairment was specific to DREADD activation, as control mice lacking hM4Di expression exhibited no WM deficits after CNO treatment (Fig. 5b, c and Supplementary fig. 3a, b). Overall, reducing CLA excitability decreased WM performance by approximately 40%, irrespective of sensory modality and the genetic strategy used to target CLA neurons (Fig. 5c, g and Supplementary fig. 3b, d).

Although DREADD-mediated inhibition enables broad and uniform suppression of targeted CLA neurons, prolonged silencing may indirectly perturb cortical circuit dynamics over extended periods, potentially confounding the interpretation of the CLA’s specific contribution to WM. Moreover, the limited temporal resolution of chemogenetic approaches precludes identification of discrete task epochs during which CLA activity is required (e.g., encoding versus maintenance). To overcome these limitations, we expressed inhibitory optogenetic actuators in either *Vglut2*⁺ or *Smim32*⁺ CLA neurons and selectively suppressed their activity during defined task epochs: sample cue presentation, early or late delay periods, or target cue presentation (Fig. 5d, e and Supplementary figs. 3-5). On average, WM performance was reduced during trials with optogenetic inhibition compared to randomly interleaved no-light trials within the same sessions in both *Vglut2*⁺ and *Smim32*⁺ cohorts (Fig. 5f, Supplementary fig. 3c and Supplementary fig. 4c). To enable direct comparisons across inter-cue delays and experimental cohorts while accounting for potential differences in baseline accuracy between cohorts (Supplementary fig. 3e and Supplementary fig. 5a,b), we defined a “relative performance index” that quantified, for each subject and delay, the change in performance relative to the corresponding control condition above chance level. Using this metric, WM performance was reduced by approximately 40–60% across both tasks (Fig. 5g, Supplementary fig. 3d and Supplementary fig. 5d). The impairment was modality-independent, consistent across all delay periods and specific to optogenetic inhibition, as light stimulation in the EYFP control group did not negatively impact WM performance (Fig. 5g, h and Supplementary fig. 3d-f).

To determine whether the timing of CLA inhibition differentially affected WM performance, we analyzed each stimulation epoch separately. Optogenetic suppression of CLA neuronal excitability consistently impaired WM performance across all task phases, irrespective of whether inhibition occurred during sample cue presentation, early or late delay, or target cue presentation (Fig. 5i and Supplementary fig. 3e, g). This effect was specific to optogenetic manipulation, as performance in the control group or in interleaved no-light trials remained stable or modestly improved across days, possibly reflecting continued task learning (Supplementary fig. 3f, g). To rule out the possibility that CLA inhibition during cue presentation disrupted sensory perception/processing rather than WM per se, we trained the same cohort of animals on Go/No-Go olfactory and tactospatial discrimination tasks, with random optogenetic inhibition of CLA neurons in a subset of trials. Performance did not differ between light and no-light trials in these sensory discrimination paradigms (Fig. 5j). In contrast, robust impairments were observed in the WM task when CLA neurons were inhibited during sample cue presentation in the same mice, indicating that the deficits reflect disrupted WM processes rather than altered sensory perception (Fig. 5k).

Given the extensive connectivity of the CLA with both cortical and subcortical regions, it is well-positioned to influence diverse brain functions, raising the possibility that CLA inhibition could elicit broader physiological effects unrelated to WM processing. Indeed, prior studies have shown that the claustro-cingulate pathway can exert complex and sometimes opposing influences on pain modulation^54–56^. To determine whether optogenetic inhibition of CLA neurons during WM tasks induced nociceptive-, stress-, or arousal-related responses, we monitored pupil size and body movements in *Smim32*-cre mice expressing eNpHR3.0 in CLA neurons, with light delivered for one second following sample cue presentation (Supplementary fig. 4). While CLA inhibition impaired WM performance (Supplementary fig. 4c), it did not produce detectable differences in pupil dynamics or gross body movements between light and no-light trials (Supplementary fig. 4d). In all conditions, movements were minimal and largely restricted to salient task epochs such as trial onset, tone and cue presentations, and animals remained behaviorally engaged even during long delays without excessive movement that could spuriously influence responses (Supplementary fig. 4a). Moreover, movement features from voluntarily controlled body parts failed to predict cue identity during either sample cue presentation or the delay period in no-light trials, indicating that animals did not rely on posture-based strategies in place of WM (Supplementary fig. 4b). Taken together, these results suggest that the observed performance impairments cannot be attributed to pain, stress, altered arousal, or gross motor dysfunction.

**Fig. 6:**
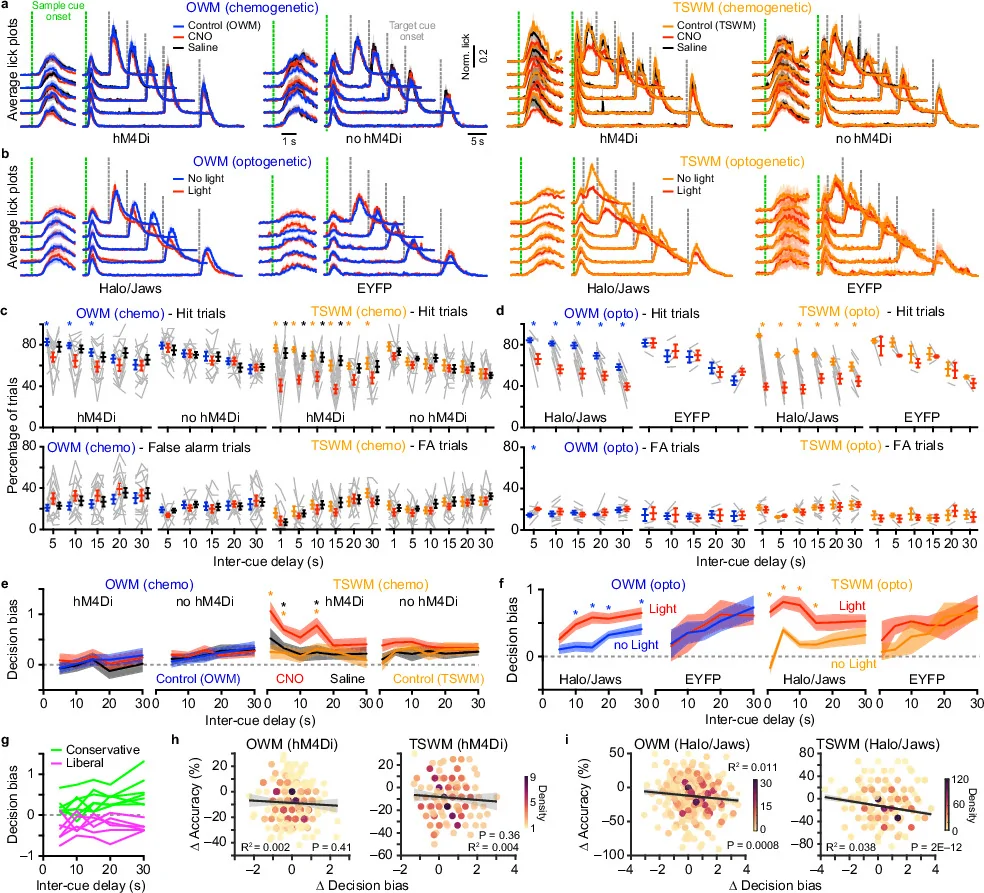
CLA manipulations influence behavioral responses and decision bias. **a**, **b** PSTH of normalized lick onset events across conditions (hM4Di: OWM: *n* = 13, TSWM: *n* = 11 mice; no hM4Di controls: *n* = 12 for both; Halo/Jaws: OWM *n* = 10, TSWM *n* = 9; EYFP: OWM *n* = 4, TSWM *n* = 3 mice). Licking after sample cue (left) and during full trial (right) are shown separately. Green and grey dotted lines mark sample and target onset, respectively. **c** Percentage of Hit and FA trials during control, CNO, and saline sessions for OWM and TSWM tasks (number of mice indicated in **a**; RM ANOVA; post-hoc Fisher’s test comparisons *: P < 0.05, significant comparisons for Control v. CNO and Saline vs. CNO are color-coded in blue/orange and black, respectively). **d** Percentage of Hit and FA trials during light and no-light trials for both WM tasks (number of mice indicated in **b**; RM ANOVA; post-hoc Fisher’s test *P < 0.05). **e** Decision bias v. inter-cue delay for mice expressing hM4Di (OWM: *n* = 13, TSWM: *n* = 11) and controls (*n* = 12 for both). Sessions included control (no injection), CNO, and saline in order (RM ANOVA; post-hoc Fisher’s test; color coding as in **c**). **f** Decision bias v. delay duration for mice expressing Halo/Jaws or EYFP. Light v. no-light trials compared within groups (Halo/Jaws: OWM *n* = 10, TSWM *n* = 9; EYFP: OWM *n* = 4, TSWM *n* = 3; RM ANOVA; post-hoc Fisher’s test). **g** Individual variability in decision bias (from **e**) as a function of delay: hM4Di-expressing mice in control OWM sessions showed divergent licking strategies (conservative v. liberal). **h** Accuracy difference (CNO – control) v. change in decision bias during chemogenetic experiment in Fig. 5b (color indicates point density, *n* = 300 and 198 mouse/session/delay triplets for OWM and TSWM, respectively). **i** Accuracy difference (light – no-light) v. decision bias change during optogenetic experiment in Fig. 5f (color shows point density *n* = 1020 and 1296 mouse/session/delay/light-period quadruplets for OWM and TSWM, respectively). Data are shown as mean ± SEM. See Supplementary Table 1 for full statistics. Source data are provided as Source Data file.

We next examined whether the reduction in WM performance could be attributed to impaired motor execution, particularly the licking responses used to report choices. Lick patterns showed no systematic differences during sample cue presentation and delays across chemogenetic or optogenetic manipulations (Fig. 6a, b), indicating intact motor output and task engagement^57^. Importantly, licking traces revealed no increase in premature or sustained licking during the delay period, even at long delays, arguing against heightened impulsivity or impaired response withholding under CLA inactivation. Instead, CLA inhibition produced a selective and reproducible reduction in hit rates (Fig. 6c, d), while false alarm rates varied across individuals, suggesting alterations in decision strategy rather than disengagement. Consistent with this, mice showed heterogeneous baseline response biases, spanning conservative to liberal strategies, and tended to adopt a more conservative response criterion as task difficulty increased (e.g. longer delays or when using tactile cues), even in control conditions (Fig. 1i and Fig. 6e-g). Although CLA inhibition sometimes increased this conservative bias, the effect was not consistent across conditions. Moreover, changes in decision bias did not reliably correlate with performance decline between manipulated and control trials (Fig. 6h,i), suggesting that WM impairments are unlikely to arise from shifts in response criterion or motor suppression.

In summary, these analyses make it unlikely that sensory deficits or nonspecific factors, including pain, altered arousal, impulsivity, impaired inhibitory control, disengagement, motor abnormalities, or decision bias, account for the performance impairments observed following chemo- and optogenetic manipulations. Instead, the findings indicate that appropriate CLA activity contributes to multiple phases of the WM task, including cue encoding, delay maintenance, and target processing. Disruption of CLA function appears to preferentially compromise the internal maintenance of task-relevant information required for WM-guided behavior. Together, these findings support a central role for the claustrum in working memory maintenance.

## Discussion

WM is widely understood to rely on distributed activity across cortical and subcortical networks, with different regions contributing to the encoding, maintenance, and readout of task-relevant information^4–6^. Numerous studies have identified brain regions that exhibit delay activity or are necessary for specific WM tasks, including prefrontal, sensory, parietal, and subcortical areas^10–16,24–39^. However, how individual nodes within this distributed architecture contribute to sustaining memory representations over time remains incompletely understood. Here, by combining population imaging with causal manipulations across multiple task phases and sensory modalities, we identify the CLA as a key contributor to the WM network.

Although persistent WM representations lasting 15–20 seconds have been documented for decades^58,59^, many recent studies have focused on shorter delays (1.5–5 seconds), where residual sensory activity may confound memory-specific coding^15,26,27,29,38^. By examining delays ranging from 1 to 30 seconds, our study extends this work to longer timescales. We find that the CLA population activity evolves dynamically throughout the delay while remaining selectively associated with cue identity, indicating that cue information is maintained within a continuously evolving ensemble state. These observations are consistent with models in which WM is supported by dynamic population activity rather than sustained firing of individual neurons^9,60,61^. In this framework, time-varying neural states support the retention of task-relevant information through structured population trajectories. Although a subset of CLA neurons exhibited movement-related activity, the majority encoded task-relevant sensory information. Notably, decoding performance closely paralleled the time-dependent fluctuations in behavioral accuracy, linking CLA population dynamics to working memory fidelity.

Causal manipulations demonstrate that CLA activity contributes to WM performance. Chemogenetic and optogenetic inhibition impaired behavior across sensory modalities and across all task phases (including encoding, delay maintenance, and target comparison) without affecting sensory processing, motor output, arousal, or inhibitory control. These findings indicate that CLA activity contributes to WM across the temporal structure of the task. In contrast to regions such as the premotor cortex, where perturbations primarily affect late-delay or response-related processes^29^, CLA inhibition produced deficits throughout the trial structure, suggesting a more pervasive contribution to WM. The magnitude and nature of the impairment varied across manipulation strategies and genetic targeting. Notably, these effects were observed despite partial perturbation of the claustral population. Our interventions reduced neuronal excitability without fully abolishing spiking, and the proportion of claustral neurons affected bilaterally was necessarily partial. Targeting was likely limited to at most ~50% of the CLA population (see Methods), due to incomplete anteroposterior viral coverage on both hemispheres^42,43^, the specificity of available Cre lines^49^, and the extended and poorly delineated anatomical organization of the CLA, which makes precise quantification challenging^45^. The nature of the perturbation also differed across approaches: chemogenetic inhibition likely induced a sustained but moderate reduction in excitability, whereas optogenetic silencing produced stronger but temporally constrained effects. Despite these limitations, a consistent pattern emerged: the strongest behavioral impairments were observed with *Smim3*2-Cre–driven optogenetic inhibition (e.g., halorhodopsin), with WM performance reduced by ~60% on average and up to ~80% in individual mice (Supplementary fig. 5c,d). The stronger impairments observed with more effective claustral inhibition are consistent with a causal contribution of CLA activity to WM, while the persistence of residual performance indicates that WM can be partially supported by other brain regions.

A key finding is that CLA inhibition during any task phase produced comparable impairments. This indicates that CLA activity contributes to WM during encoding, delay maintenance, and target comparison, rather than being restricted to a single temporal stage. During encoding, sensory inputs may engage CLA neuronal assemblies that support the formation of memory representations. Disrupting this initial activation appears to interfere with the subsequent formation or stabilization of the memory representation. This is particularly notable given that sensory cortical activity is likely still present during early delay periods, yet appears insufficient to restore performance once CLA activity is perturbed. Mechanistically, CLA activity during cue presentation is likely driven in part by cortical inputs, while sustained activity during the delay may also depend on intra-claustral interactions. Although early models emphasized feedforward inhibition^62^, recent evidence indicates the presence of local excitatory connectivity among claustral projection neurons^63^, which could support persistent activity. Future work will be required to identify the circuit mechanisms underlying cue selectivity and the maintenance of claustral activity during WM.

Disrupting CLA activity at different stages of the task interfered with WM performance, indicating that CLA activity contributes to multiple stages of the ongoing neural processes supporting WM. Consistent with this, CLA inhibition produced broadly similar impairments across delays and task phases, arguing against a role restricted to a specific temporal stage or delay-dependent process. Instead, these findings suggest that CLA activity supports WM representations across multiple stages of the task, potentially by maintaining their strength or stability within a distributed network. The anatomical organization of the CLA provides a plausible substrate for such a role. Its extensive reciprocal connectivity with a wide range of cortical regions^40–43^ positions it to influence distributed activity patterns in a context-dependent manner. Within this framework, the CLA may contribute to sustaining task-relevant activity across cortical circuits rather than functioning as an isolated storage site. This view is broadly consistent with cognitive models that include intermediate buffers for temporarily holding information^1,64^, although our data suggest that any such function is embedded within distributed and dynamically interacting networks. Consistent with this interpretation, CLA inhibition produced relatively similar reductions in performance across delays, with no consistent evidence for a delay-dependent increase in impairment, arguing against a selective acceleration of memory decay. In recurrent network models of WM, the stability of population dynamics, which is limited by noise-induced drift, directly determines the fidelity of maintained information and behavioral performance^65,66^. Together, these observations support a model in which the CLA functions as an integrative node that contributes to the stabilization and maintenance of memory-related activity within distributed WM circuits.

After the preprint version of this work was posted^67^, and while the present manuscript was under revision, another study reported that *Vglut2*-negative neurons in the claustro-insular region projecting to the medial prefrontal cortex are active during successful maintenance of spatial working memory^68^. Consistent with our findings, optogenetic silencing of these neurons during the delay period impaired task performance. Interestingly, however, silencing during the decision-making phase enhanced behavioral performance. These results are broadly consistent with our conclusion that the CLA contributes to the maintenance of WM, extending this role to the spatial domain. However, the opposite effect observed during the decision-making phase differs from our findings. This discrepancy may reflect differences in task design, including the use of a freely moving T-maze paradigm in the previous study versus a head-fixed delayed non-match-to-sample task in our experiments. For example, the medial prefrontal cortex is dispensable for performance in the DNMS task once initial training has been completed^15^, whereas it plays a well-established role in spatial navigation and T-maze performance^69^. Such differences could contribute to the distinct effects observed during the decision-making phase. Alternatively, the difference may arise from the neuronal populations targeted. Given the cellular heterogeneity of the claustro-insular region, the targeting of *Vglut2*-negative neurons is unlikely to encompass a single homogeneous population^45^. Transcriptomic analyses and transgenic mouse lines indicate that not all claustrum neurons express *Vglut2*^45,49^. Consequently, the targeted population may include *Smim32*-positive/*Vglut2*-negative claustrum neurons, which could overlap with the neuronal population examined in our study. However, it may also include genetically distinct neuronal populations located within the claustrum “shell” and/or adjacent insular cortex, a cortical area also contributing to WM processes^70^. Future studies using more selective genetic driver lines will be required to determine the respective contributions of these distinct neuronal populations to WM maintenance and decision-making.

In conclusion, our findings identify the CLA as an important component of the WM network. By sustaining task-relevant population activity over time and contributing to WM performance across sensory modalities and task phases, the CLA emerges as a key node in the distributed neural architecture supporting WM maintenance.

## Methods

### Animals

A total of 61 mice (31 males and 30 females), aged between 3 and 11 months (at the end of some experiments), were included in the study. No sex differences were detected, and data from both sexes were pooled for all analyses. All experiments were conducted using either *Slc17a6*^*tm2(cre)Lowl*^/J heterozygote mice (referred to as *Vglut2*-cre in the text; the Jackson Laboratory, strain number 016963), Tg(*Smim32*-cre)61^*Irod*^ or *Smim32*-Cre knockin heterozygote mice (14 males and 17 females), or Tg(*Smim32*-cre)61^*Irod*^;*Gt(ROSA)26Sor*^tm1(CAG-CHRM4*,-mCitrine)Ute^/J double heterozygote mice, which were obtained by crossing *Gt(ROSA)26Sor*^tm1(CAG-CHRM4*,-mCitrine)Ute^/J homozygote mice^71^ (the Jackson Laboratory, strain number 026219) with Tg(*Smim32*-cre)61^*Irod*^. In *Vglut2*-cre mice, the cre coding sequence does not disrupt the *Slc17a6* gene coding sequence since it was placed after the stop codon^72^. The *Smim32*-Cre knock-in mouse line was generated by inserting an IRES-Cre recombinase cassette immediately downstream of the endogenous *Smim32* stop codon, thereby preserving endogenous *Smim32* expression. The Tg(*Smim32*-Cre)61Irod transgenic line was generated by introducing a Cre-expressing transgene under the control of the *Smim32* regulatory elements using a bacterial artificial chromosome (BAC). A detailed characterization of these mouse lines is available at^49^.

Housing conditions involved maintaining mice in groups of 2-5 individuals, following a standard 12 h light/dark cycle, within a room maintained at 24 °C, with *ad libitum* access to food. Prior to behavioral experiments, mice underwent a 12-14 h period of water restriction. After the behavioral sessions, mice were provided unrestricted access to water until satiety within their housing cages. All experiments were conducted during the light phase of the light/dark cycle. All experiments adhered to the ethical guidelines outlined in the Swiss Federal Act on Animal Protection and the Swiss Animal Protection Ordinance. The experimental protocols received approval from the University of Geneva and the Geneva state ethics committees (Licenses GE/94/20, GE368 and GE369).

### Preparation of mice for surgeries and post-operative care procedures

Mice were anesthetized using isoflurane at concentrations of 3-5% for induction and 1.5-2% for maintenance. Prior to any intervention, the skin overlying the skull was disinfected with a betadine solution and subsequently removed under local anesthesia using 25 µl of 0.25% carbostesin. To mitigate the risk of cortical edema during craniotomy, mice were administered an intraperitoneal injection of dexamethasone (20 µl at 4 mg/ml). For the procedure, mice were securely positioned using ear bars and a nose clamp on a stereotaxic apparatus (Stoelting) to ensure stability and precision. To prevent ocular dehydration, artificial tears (Lacryvisc™ eye gel) were applied, while the body temperature was monitored and maintained at ~37 °C using a heating pad. Specific surgical procedures were then performed as dictated by experimental requirements (see details below). Following completion, to counter any dehydration during anesthesia, a subcutaneous injection of 0.9 *%* NaCl ( ~ 500 µl) was administered. Furthermore, post-operative care included the provision of analgesia through a subcutaneous injection of carprofen (150 µl at 1 mg/ml) and the addition of paracetamol (2 mg/ml) to the drinking water for three days.

### Adeno-associated virus injections

AAV2-hSyn-DIO-hm4D(Gi)-mCherry (Titer GC/ml: 4.6×10^12^, Addgene catalog #: 44362-AAV2), AAV1-Syn-Flex-GCaMP6f.WPRE.SV40 (Titer GC/ml: 1.9×10^13^, Addgene catalog #: 100833-AAV1), AAV1-syn-FLEX-jGCaMP8f-WPRE (Titer GC/ml: 5.9×10^12^, Addgene catalog #: 162379-AAV1), AAV2-Ef1a-DIO-EYFP (titer: 4.5×10¹² GC/ml; UNC GTC vector core; catalog #: AV4842F), AAV1-Ef1a-DIO-eNpHR3.0-EYFP (titer: 2.1×10¹³ GC/ml; Addgene catalog #: 26966-AAV1), and AAV1-CAG-FLEX-rc[Jaws-KGC-GFP-ER2] (titer: 2.5×10¹³ GC/ml; Addgene catalog #: 84445-AAV1) were used in the experiments. Following the preparation of mice for surgical procedures, craniotomies were performed either unilaterally or bilaterally above the CLA using an air-pressurized driller. Glass pipettes filled with AAV solutions were inserted in one hemisphere at a time at the following coordinates: antero-posterior (AP) 1.1 mm and medio-lateral (ML) ± 2.85 mm relative to bregma, dorso-ventral (DV) −2.4 mm relative to the brain surface. Approximately 100 nl of the AAV solution was deposited per injection site. The pipette remained in place for 5 minutes before removal. For chemogenetic and optogenetic experiments, infections were bilateral, while for microendoscopic imaging experiments, infections were unilateral. Following viral injections, additional surgical procedures were performed based on the specific experiment requirements. A minimum period of three weeks was allowed for viral expression before mice underwent behavioral experiments.

### Headpost implantations

After securing the mouse on the stereotaxic apparatus, the overlying skin was removed, and the surgical site was cleaned with sterile saline. The overlying muscle and periosteum were gently removed from the skull, which was then allowed to air-dry. Gelfoam^®^ soaked in sterile saline was applied in case of minor bleeding to facilitate clotting. The skull was chemically etched using a dental etching agent to enhance surface roughness for better adherence to dental cement. Any excess etching agent was cleaned with saline, and the surface was air-dried. A thin coat of cyanoacrylate gum (Cyberbond) was applied to prevent fluid seepage and reinforce the skull for improved cement adherence. A dental primer was applied, air-dried, and polymerized with UV light. UV-polymerizing cement was then spread over the skull, fused with the periphery and polymerized. The head-post, coated with UV-polymerizing cement, was positioned and polymerized. Dental acrylic cement, mixed with a polymerizing agent and cyanoacrylate gum, was used to seal exposed areas on the skull.

### GRIN lens implantation

After preparing the mouse for surgery, a craniotomy was performed over the CLA. Following a unilateral injection of AAV1-Syn-Flex-GCaMP6f.WPRE.SV40, a 0.5 mm diameter GRIN snap-in imaging cannula (Model L, Doric Lenses Inc., Canada) was gradually lowered into the brain by approximately 2.35-2.45 mm at antero-posterior (AP) 1.1 mm and medio-lateral (ML) 2.85 mm relative to bregma. The GRIN cannula was securely affixed to the skull using cyanoacrylic glue (Vetbond™) and dental cement. Following the GRIN lens assembly, a head-post was attached to the skull following the aforementioned procedures. Mice were allowed a recovery period of at least three weeks post-surgery. After the recovery period, the optimal imaging plane was determined by finely adjusting the GRIN lens using the protrusion adjustment rings provided with each cannula. The lens position was then fixed by applying cyanoacrylic glue between the cannula and protrusion adjustment rings.

### Delayed Non-Match to Sample (DNMS) working memory tasks

Prior to starting behavioral experiments, the mice underwent a brief water restriction period lasting 12-14 hours. Before each session, mice were gently placed in a plastic/PVC tube and their head was securely restrained using a specialized metallic device affixed to a stable platform, as previously described^73,74^. All experiments were conducted using custom-made, computer-controlled olfactometers, following established protocols^75,76^. These olfactometers were designed to ensure precise delivery of sensory stimuli (tested with photoionization detectors^77^), including odorants or a clean airflow, and to accurately measure lick responses within predetermined time windows^73^. This was achieved through the integration of a licking circuit and a metallic water port.

In the DNMS WM tasks, each trial began with a 2 s baseline, followed by the presentation of a sample cue for 1 s, succeeded by a variable delay period, and then a target cue presented for 1 s, which could either be the same (match trial) or differ from the sample cue (non-match trial). Mice underwent pre-training to initiate licking responses specifically within a response window during non-match trials (see next section). The 2.5 s response window began 0.5 s after the target cue onset. Trials were separated by a fixed inter-trial interval of 10 s. To facilitate discrimination between trial onset and the inter-cue delay period, a brief 0.2 s tone was presented at the beginning of each trial as a trial-onset cue. Tone frequency varied between 3 and 9 kHz across animals but remained constant for each individual mouse. Behavioral sessions were conducted simultaneously across three to four rigs, with each mouse consistently trained on the same setup. Although auditory crosstalk between rigs was minimized, assigning distinct tone frequencies further ensured that each animal reliably associated its specific tone with trial onset.

During training, mice learned to associate non-match trials with licking within a designated response window to obtain a water reward. However, we observed frequent licking during sample cue presentation and occasional anticipatory licking during the delay period (Fig. 1d,e and Supplementary fig. 1a). To account for this behavior and ensure accurate trial classification, licking was monitored throughout the entire trial. A non-match trial was classified as “Hit” only if the number of licks during the response window exceeded those occurring during the pre-response period, defined as the interval from sample cue onset to the start of the target cue. Trials that did not meet this criterion were classified as “Misses”. Upon a Hit, mice received an immediate reward ( ~ 5 µl water). False Alarm trials were defined analogously: a match trial was classified as a False Alarm if licking during the response window exceeded pre-response licking. False Alarms were not punished, and no reward was delivered for Misses or Correct Rejections. Because licking during the sample cue and throughout the full delay period (up to 60 s) was included in the pre-response measure, this conservative definition penalized excessive or anticipatory licking that could not be compensated for during the relatively brief response window. To verify that trial classification was not biased by the variable duration of the pre-response period, we performed a post hoc reclassification using an artificial fixed baseline. This baseline was constructed by combining 2.5 s from sample-cue onset with 2.5 s immediately preceding the response window, thereby capturing periods in which sample-related and anticipatory licking were most likely to occur. This reanalysis was not performed for 1 s TSWM trials because the available pre-response period was insufficient for constructing the fixed baseline. Each mouse performed approximately 100 trials per day, all of which were included in the analysis. Trial events, including stimulus timing, licking behavior, and reward delivery, were automatically recorded by the behavioral control system.

In this study, we assessed WM across distinct sensory modalities, namely, olfactory WM (OWM)^15,70,78^ and tactospatial WM (TSWM). As our focus was on OWM rather than olfactory perception or discrimination, we used various monomolecular odorants (ethyl acetate, 2-pentanone, 1,4-cineole, eugenol, amyl acetate and ethyl butyrate; Sigma-Aldrich) known to be easily discriminated by mice^48,73,79^ and not requiring a functional reorganization of olfactory responses such as pattern separation associated to learning^80,81^. The odorants were initially diluted in mineral oil (2% odorant dilution) and further mixed with a clean carrier airflow (odorant: carrier ratio of 1:2), ensuring activation of multiple olfactory bulb glomeruli^79,82^ without eliciting the large responses observed following natural odorant application^84^. We did not synchronize odor application with respiration to minimize equipment crowding near the mouse snout. Consequently, we opted for a 1 s odor application to ensure at least two complete respiratory cycles during odorant presentation while minimizing strong olfactory afterimages in the olfactory system, which could complicate WM performance comparisons across sensory modalities^77^. For the same reason, we did not test the 1 s inter-cue delay in the OWM task to reduce the potential impact of residual olfactory afterimages as confounding factors in WM performance. Counterbalancing was employed to evenly distribute the selected odorants as sample and target cues across different trials.

In the TSWM task, we used identical equipment to administer a gentle air puff (400 ml/min) directed towards either the left or right whisker pad (Fig. 1a). During each trial, if the air puff was directed to the same whisker pad for both the sample and target cues, it was categorized as a match trial. Conversely, if the air puff was delivered to opposite whisker pads, the trial was classified as non-match. Additionally, we implemented counterbalancing of the air puff direction as sample and target cues across different trials.

The comprehensive behavioral experimental process included habituation, pre-training, training, and the evaluation of WM through DNMS tasks. During habituation, mice were gently head-fixed in behavioral setups and introduced to drinking water from the lick port, aided by manually delivered water. Once mice became accustomed to head restriction, the pre-training began. In the initial phase (20 trials), a water drop (2-3 µl) was promptly presented to mice following a 0.2 s tone. Subsequent trials introduced increasing delays between the tone and water reward, progressing to 2 s (10 trials) and 5 s (10 trials), aimed at eliciting licking behavior and dissociating the tone from water delivery. Upon mastering the ability to wait for water rewards, pre-training shifted focus exclusively to non-match trials. Ethyl acetate and 2-pentanone served as stimuli, randomly assigned as sample and target cues across trials. Sample and target cues were presented with a fixed inter-cue delay of 5 s. In the initial 20 trials of this phase, an automatic reward was delivered at the onset of the response window. Following this, the automatic reward was withheld, and any licking event during the response period triggered water delivery (20-40 trials). Once mice started licking regularly in the response period, the task rules were implemented. A correct trial now necessitated a higher count of licks during the response window compared to the pre-response window. Typically, mice took approximately 150-200 trials to learn the task rules and consistently exhibited licking behavior during the response window. Following this, the training phase for OWM was initiated, introducing a pseudorandomized implementation of both match and non-match trials. During this phase, 1,4-cineole and eugenol were used as stimulus cues and the inter-cue delay was maintained at 5 s. Specifically, one or two non-match trials and one or two match trials were presented in a randomly sequenced pattern, appearing in blocks of two or four trials. Subsequently, the trials were pooled together, and performance accuracy was determined by calculating the percentage of correct trials within each block of 100 trials. The performance accuracy, denoted as ‘Accuracy (%)’ in figure labels, is defined as follows in Eq. (1):

1 $${{\rm{Performance}}}\,{{\rm{accuracy}}}=\frac{{{\rm{number}}}\,{{\rm{of}}}\,{{\rm{hit}}}\,{{\rm{trials}}}+{{\rm{number}}}\,{{\rm{of}}}\,{{\rm{correct}}}\,{{\rm{rejection}}}\,{{\rm{trials}}}}{{{\rm{total}}}\,{{\rm{number}}}\,{{\rm{of}}}\,{{\rm{trials}}}}$$

In addition to performance accuracy, we extended our evaluation to include the sensitivity index and decision bias. While sensitivity index quantifies the ability to discern the relevant signal from background noise, offering a quantitative measure of mice’s capability to make accurate discriminations, decision bias is a parameter reflecting mice’s decision-making strategy (propensity to lick during response window). The calculation of sensitivity index and decision bias involved determining the Hit Rate (HR) and False Alarm Rate (FA) as follows in Eqs. (2) and (3):

2 $${{\rm{Hit}}}\,{{\rm{Rate}}}\,({{\rm{HR}}})=\frac{{{\rm{number}}}\,{{\rm{of}}}\,{{\rm{hits}}}}{{{\rm{number}}}\,{{\rm{of}}}\,{{\rm{hits}}}+{{\rm{number}}}\,{{\rm{of}}}\,{{\rm{misses}}}}$$

3 $${{\rm{False}}}\, {{\rm{alarm}}}\, {{\rm{rate}}}\, ({{\rm{FA}}})=\frac{{{\rm{number}}}\, {{\rm{of}}}\, {{\rm{false}}}\,{{\rm{alarms}}}}{{{\rm{number}}}\, {{\rm{of}}}\, {{\rm{false}}}\, {{\rm{alarms}}}+{{\rm{number}}}\, {{\rm{of}}}\, {{\rm{correct}}}\, {{\rm{rejections}}}}$$

Sensitivity index is defined by the Eq. (4):

4 $${{\rm{Sensitivity}}}\,{{\rm{index}}}\,=\,{{\rm{norminv}}}({{\rm{HR}}})-{{\rm{norminv}}}({{\rm{FA}}})$$

Decision bias is then defined by the Eq. (5):

5 $${{\rm{Decision}}}\,{{\rm{bias}}}=-0.5({{\rm{norminv}}}({{\rm{HR}}})+{{\rm{norminv}}}({{\rm{FA}}}))$$

where norminv is the inverse of the cumulative normal distribution function. To prevent issues related to infinite values, we applied a conversion to Hit and False Alarm rates. If either rate equaled 100%, it was adjusted to 0.99, and if the rate was 0, it was set to 0.01 during the conversion process.

In most instances, mice started performing with >70% accuracy in blocks of 10 trials within ~400 trials (Fig. 1b). To ensure the stability of their acquired skills, training was extended up to 800 trials. Once mice were proficiently trained, they were exposed to DNMS tasks with variable delay periods, all within a single session. The delay periods were pseudo-randomly varied in blocks of two or four trials (counterbalanced for rewarded and non-rewarded trials), encompassing delay periods of 5 s, 10 s, 15 s, 20 s, 30 s, 40 s, and 60 s. Each session consisted of 100 trials, with each delay period presented for 14-16 trials within the session. By maintaining a fixed number of trials at 100, we ensured that each mouse completed all trials within a single session. To bolster the robustness of our observations, mice underwent three such sessions across three consecutive days (Supplementary fig. 1e,f). This approach not only facilitated a comprehensive investigation of the mice’s response to variable delays but also enabled a thorough analysis by ensuring an adequate number of trials for each specific delay condition. In order to assess the generalizability of performance and ensure that results were not solely odor-specific, 12 out of the total 30 mice additionally underwent testing with amyl acetate and ethyl butyrate. Following confirmation of consistent performance in the DNMS task across various odors (Supplementary fig. 1g,h), performance was averaged across different odor sessions. This averaged performance data was then utilized for the analyses presented in Fig. 1.

In the TSWM DNMS tasks, mice were trained with a 1-second delay between sample and target stimuli. As TSWM training followed the completion of OWM tasks, mice had already mastered the procedural elements of the task paradigm. TSWM training specifically aimed to familiarize mice with the shift in sensory modality, teaching them to detect and distinguish the directionality of whisker pad stimulation. Remarkably, mice successfully adapted to this sensory shift within the initial 100-150 trials. To ensure proficiency in TSWM, all mice underwent training for up to 600 trials. Additionally, for TSWM with variable delays, an extra delay of 1 s was introduced alongside the delays used for OWM. All other aspects of the task design remained consistent with those of the OWM.

The accuracy and sensitivity index values of the WM tasks at varying delay durations were modeled using an exponential decay function in GraphPad Prism 9.0. We fixed the plateau to a constant value, set at 50 for accuracy and 0 for sensitivity index values, representing the theoretical chance level for the behavior. The function was defined by the Eq. (6):

6 $${{\rm{Y}}}=({{\rm{Y0}}}\,-\,{{\rm{Plateau}}})*\exp (-{{\rm{K}}}*{{\rm{X}}})+\,{{\rm{Plateau}}}$$

Using this equation, we calculated Y0, representing the extrapolated amplitude at X = 0, and the half-life, computed as ${\mathrm{ln}}\left(2\right)/K$.

Lick behavior was recorded using an integrated lick-detection circuit, capturing binary lick events per trial at a temporal resolution of 2 ms. Lick initiation events were tallied in non-overlapping 100 ms bins and normalized by the total number of trials per condition. The normalized lick initiation events were then plotted across different inter-cue delays and facilitated comparison of trial engagement during tasks with chemo- or optogenetic manipulations (Fig. 1d,e, Fig. 6a,b, Supplementary fig. 1a). Additionally, the absolute licking counts and ranges during the pre-response and response windows are calculated from individual mouse-session data and represented as box plots indicating the median, interquartile range, and outliers (Supplementary fig. 1b).

### Microendoscopic calcium imaging

Microendoscopic imaging was performed by genetically expressing GCaMP6f in CLA projection neurons expressing either *Vglut2* (Vglut2-Cre mice; 4 males and 3 females; data presented in Figs. 2–4) or *Smim32* (Tg(*Smim32*-cre)61^*Irod*^ mice; 4 males and 3 females; data presented in Supplementary fig. 2). Imaging acquisition utilized a snap-in fluorescence microscope body (2CFM model L Type D, Doric Lenses Inc., Canada) mounted on the implanted GRIN cannula. The Ce:YAG fiber light source (465 nm output, Doric Lenses Inc., Canada) was adjusted within the range of 1-2.7 mW. The field of view, corresponding to 320 µm x 320 µm, was captured at a spatial resolution of 600 pixels x 600 pixels and a frame rate of 10 Hz. Image processing involved the use of ImageJ (National Institutes of Health, USA), Doric Neuroscience Studio (Doric Lenses Inc., Canada), and custom MATLAB (MathWorks, Inc.) routines. On imaging days, mice completed the entire session of 100 trials, with only the 5 s, 15 s, and 30 s delay trials being imaged. Synchronization with the trials was achieved through an external TTL signal from the olfactometer software. Images were acquired over three days to ensure an adequate number of trials for each specific delay condition.

Images from different trials of a given session were concatenated using a custom script written in MATLAB. Individual image frames were corrected for infocal (XY) plane brain motion using the alignment function integrated into Doric Neuroscience Studio and the maximum projection of the concatenated trace served as a reference for the motion correction. Following motion correction, the edges of the images were cropped by ~5 pixels, and the trimmed images were downsampled by a factor of two using bilinear interpolation in MATLAB. Additionally, a Kalman stack filter in ImageJ was applied to remove high-gain noise from the images. To identify neurons and detect Ca^2+^ transients, we used a cell-sorting algorithm^83^ implemented in MATLAB. This algorithm integrates principal and independent component analyses (PCA-ICA) and is coupled with a segmentation routine optimized to minimize the occurrence of false positives. To remove slow trends and preserve faster dynamics in the signal, we applied discrete wavelet transformation to the traces from each neuron. After detrending, the traces were split into individual trials. For normalization, a baseline vector was constructed from all trials of a given neuron, with the baseline defined as the period 1 s before sample presentation. The median of this baseline vector was then used to calculate z-scored traces following Eq. (7):

7 $$y{{\rm{i}}}=\frac{{{\rm{xi-median(baseline)}}}}{{{\rm{median}}}(|{{\rm{xi-median}}}({{\rm{baseline}}})|)}$$

Neurons recorded from different mice were grouped to generate a high-dimensional neuronal population. To characterize the functional diversity of CLA neurons during WM tasks, we classified neurons into three categories: Stimulus neurons, Delay neurons, and Inhibited neurons. This classification was based on their temporal response profiles, specifically the latency of their inferred spike onset relative to stimulus presentation. We first computed the z-scored trial-averaged calcium traces for each neuron using correct trials only. The calcium fluorescence signals were then deconvolved using a fast nonnegative deconvolution method^85^, which inferred spike trains from calcium activity. A detection threshold of 1.5 z-scores was applied to define spike onset events. To categorize neurons, we analyzed the timing of the first inferred spike relative to the stimulus presentation. Neurons whose first spike occurred during the sample presentation window were classified as Stimulus neurons, as their activity likely reflects immediate cue-driven encoding. Neurons whose first spike occurred after the offset of the stimulus were classified as Delay neurons. Neurons that showed no above-threshold spikes during the task were designated as Inhibited neurons, reflecting task-induced suppression or low excitability. We note that virtually all recorded neurons showed significant activity changes at some point during the delay across the different delay durations. Because non-responsive neurons cannot be reliably identified from miniscope recordings and because we applied conservative criteria for identifying true calcium events, the reported percentages reflect only the subset of claustral neurons for which detectable activity changes could be measured, rather than the entire underlying neuronal population. To determine whether observed activity during the first spike exceeded chance levels, each neuron’s z-scored activity was circularly shifted ( ± 500 ms) 1000 times to generate a null distribution. A neuron was considered significantly active if its maximum mean activity at the first-spike latency bin exceeded the 95^th^ percentile of the shuffled distribution. Using this criterion, ~90% of Stimulus and Delay neurons exhibited statistically significant firing fields across all delays for both OWM and TSWM tasks. This classification scheme offers a nuanced understanding of the temporal dynamics and response characteristics exhibited by different neuron populations (Fig. 2b,d). The activity heat maps were generated by plotting the Inhibited neurons first, followed by the Stimulus neurons, and then the Delay neurons and visualized as a pseudo-color heat map using MATLAB (Fig. 2c, Supplementary fig. 2b).

Alternatively, to avoid reliance on arbitrary categorization thresholds and to adopt a more data-driven approach, we performed an unsupervised clustering analysis. Specifically, we applied k-means clustering to neural response profiles from correct trials, analogous to the response latency–based classification. First, neuronal responses were averaged across correct trials, and k-means clustering was conducted across a range of cluster numbers (k-values from 2 to 20) representing potential neuronal response classes. For each k-value, the sum of squared errors (SSE) was calculated, and the optimal number of clusters was determined using the elbow method, which identifies the point of diminishing returns based on the second derivative of the SSE curve. Using this optimal cluster number, k-means clustering was performed. Subsequently, a two-dimensional visualization was generated via t-distributed stochastic neighbor embedding (t-SNE), computed from the correlation matrix of the calcium activity data, with each neuron color-coded by its assigned cluster (Fig. 2f). Finally, heatmaps of neuronal activity were generated by grouping neurons according to cluster identity and sorting them within each cluster based on response latency (Fig. 2h, Supplementary fig. 2d,e).

We did not attempt to compare the representations evoked by cues across different sensory modalities due to the separation of imaging sessions by several weeks, raising uncertainty about whether the sampled CLA neurons were consistent across these time points.

To gain insights into the trial-dependent population response dynamics, temporal correlations were calculated for four distinct trial structures (sample-target): A-A, A-B, B-A, and B-B. To compute the temporal correlation matrices, a population vector with N dimensions was constructed, where N denotes the total number of responsive neurons recorded across all mice. Within each dimension, we calculated the mean calcium z-score amplitude averaged across all correct trials for all task-delay pairs every 100 ms. Consequently, the temporal evolution of CLA population activity was generated by calculating pairwise Pearson’s linear correlations between each pair of time bins, where each time bin corresponds to a duration of 100 ms. The resulting correlation matrix was visualized as a pseudo-colored heat map (Fig. 3a, Supplementary fig. 2g). To further grasp the temporal progression of population activity, we compared all time vectors to those recorded at the peak response time of stimulus neurons and at the conclusion of each delay period (Fig. 3b,c).

To evaluate neuronal selectivity to stimulus presentation, we computed the cross-correlation between neuronal activity and the timing of sample and target cues across the entire session, regardless of stimulus identity (A or B). For each neuron, Pearson’s correlation coefficient and corresponding significance (p < 0.05) were calculated. Cross-correlograms were generated, and the maximum absolute correlation coefficients along with their associated time lags were retained. To assess the statistical significance of stimulus modulation, we performed permutation testing using 100 circular time shifts ( ± 5 s). Neurons were classified as stimulus-correlated if they met all three criteria: (1) a significant Pearson correlation (p < 0.05), (2) a response significantly different from permuted controls, and (3) a response lag within ±500 ms of stimulus onset. The results were visualized as heatmaps of z-scored peri-stimulus average activity, sorted by response latency (Fig. 4a).

Next, a two-step analysis was undertaken to identify neurons with stimulus-specific responses (i.e., sample A or sample B in Fig. 4b,c). Firstly, the average responses were calculated from the correct trials where either stimulus A or stimulus B served as the sample for each neuron separately. Subsequently, for each neuron, a two-sided Wilcoxon rank sum test was conducted, comparing the activity during the sample presentation to discern whether the neuron exhibited specificity to stimulus A or B. The neurons displaying specificity to a particular stimulus were then visually represented through a pseudo-colored heat map. These selective neurons were sorted based on the latency of their maximum activity to enhance interpretability. This particular ordering of neurons was maintained to visualize their responses toward the non-specific stimulus. For the analysis of population trajectories, the average responses of all neurons toward stimuli A and B (from correct trials) were concatenated to construct a data matrix with rows representing individual neurons and columns representing time points. To visualize this high-dimensional data, we performed Principal Component Analysis (PCA), and the resulting first three Principal Components (PCs), which are eigenvectors with the largest eigenvalues of the covariance matrix of the original firing patterns, were used to generate trajectories shown in Fig. 4e.

### Logistic regression-based Classifier Analysis

To quantify whether sample information is maintained in neural population activity during the delay period, we trained a regularized logistic regression (linear) decoder to discriminate the two sample classes. Because the number of features (neurons) was large relative to the number of trials, we used L2 (ridge) regularization to reduce overfitting and improve generalization. Models were fit in MATLAB using ‘fitclinear’ (Learner = ‘logistic’, Regularization = ‘ridge’, Lambda = 0.1, Solver = ‘lbfgs’, uniform class priors).

Neural activity was analyzed separately for each delay condition (5 s, 15 s, 30 s). For each delay, trials were partitioned into groups defined by (Mouse × Task) combinations and arranged as 3D arrays (N_neurons_ × N_time_ × N_trials_). Two classes were defined using correct trials: Class A and Class B, depending on whether sample A or sample B was presented. Incorrect trials were assembled in parallel but were never used for model training. Within each group, correct trials were split using a stratified random partition (70% train / 30% test), performed independently within each class; incorrect trials were subsampled ( ~ 30% per class when possible, otherwise all available trials were retained) for evaluation only.

For each condition, the decoder was trained on population activity from individual group spanning the sample and delay window, and performance was quantified on held-out correct test trials as well as on incorrect trials. The trained weights were then applied to evaluate decoding performance at regularly spaced time points across the delay period: early delay, defined as the first second after sample offset; 1 s windows centered at one-quarter, mid-delay, and three-quarter delay; and late delay, defined as the final second before target presentation. This allowed us to track how stimulus information evolved across the trial. Decoder reliability was assessed using repeated stratified resampling (50 iterations) and a label-shuffled control in which training labels were randomly permuted to estimate chance-level performance while preserving the data structure.

### Lick event-locked analysis of calcium population activity

To assess neuronal selectivity to licking behavior, we computed the cross-correlation between neuronal activity and lick events. Raw lick data were downsampled to match the calcium imaging frequency (10 Hz). To differentiate lick modulation from stimulus-induced responses, lick events during the sample and target presentation phases were masked. Pearson’s correlation coefficient and its significance (p < 0.05) were calculated for each neuron. We computed cross-correlograms, retaining maximum absolute coefficients and associated lags. Statistical significance of lick modulation was evaluated via permutation tests (100 circular shifts ±5 s). Neurons were classified as lick-correlated based on significant Pearson correlation (P < 0.05), significant responses compared to permuted traces, and a response lag within ± 500 ms of lick onset. Results were visualized as heatmaps of z-scored peri-lick averaged activity sorted by response latency.

### Chemogenetic manipulation

We used two complementary approaches to inhibit CLA neural activity during behavior. In the first approach, *Vglut2*-Cre mice received bilateral injections of a Cre-dependent AAV encoding the inhibitory DREADD receptor hM4Di (AAV2-hSyn-DIO-hM4Di-mCherry) into the CLA. Activation of hM4Di by clozapine-N-oxide (CNO)^53^ induces Gi-coupled signaling, resulting in robust suppression of neuronal excitability. This strategy enabled localized inhibition of CLA neurons within the infected regions. Given the elongated anatomy of the CLA, which limits complete coverage with focal viral infections, we employed a second approach to achieve broader modulation. We used Tg(*Smim32*-Cre)61^Irod^ heterozygous mice crossed with a Cre-dependent hM4Di reporter line Gt(ROSA)26Sortm1(CAG-CHRM4*,mCitrine), allowing DREADD expression without viral delivery. In the claustro-insular region, *Smim32* is a selective marker of CLA projection neurons^45^, and prior characterization of this transgenic line demonstrated that Cre is expressed in approximately 70% of *Smim32*⁺ CLA neurons^49^. Because the endogenous *Smim32* mRNA labels a substantial fraction of CLA neurons (defined as ~80% of *Nr4a2*⁺ and ~65% of *Gnb4*⁺ populations), this approach is estimated to target roughly 50% of the total CLA population. However, the exact proportion may vary depending on the criteria used to define CLA identity, owing to the complexity of claustro-insular organization and the lack of complete specificity of *Nr4a2* and *Gnb4* for the CLA^45^.

In the chemogenetic inhibition experiments, mice participated in a sequence of OWM and TSWM sessions. The experimental timeline involved three initial sessions without treatment, establishing a baseline for the mice’s behavioral performance. Subsequently, mice received CNO treatment for three sessions, administered over three consecutive days. Following the CNO treatment, an additional three sessions were conducted with saline administration. Saline sessions served as an internal control, enabling behavioral comparisons before and after DREADD activation while accounting for potential injection-related stress. This design allowed us to evaluate the effects of chemogenetic inhibition on WM across different sensory modalities. The chemogenetic manipulation was carried out 30 minutes after the intraperitoneal injection of 60 µl of CNO (2 mg/kg, TOCRIS Bioscience) or after the administration of saline (60 µl of 0.9% NaCl). By incorporating both treatment and control sessions, we were able to isolate changes in cognitive performance attributable to chemogenetic modulation of neuronal activity. To control for potential non-specific effects of CNO, we performed parallel experiments in a DREADD-free cohort of *Vglut2*-Cre mice injected unilaterally in the CLA with pGP-AAV1-syn-FLEX-jGCaMP8f-WPRE. These mice were excluded for imaging analyses because they did not exhibit detectable calcium signals during initial screening. Thus, they were used as controls and underwent the same behavioral protocol, including baseline (no injection), CNO, and saline sessions. To quantify the impact of CNO on working memory (WM), we calculated the area under the curve (AUC) of performance across delays (5–30 s for OWM and 1–30 s for TSWM) using GraphPad Prism 9.0.

For OWM, the hM4Di group comprised 7 *Vglut2*-Cre and 6 Tg(*Smim32*-Cre):Rosa-lSl-hM4Di mice (9 males, 4 females), while the DREADD-free control group (no hM4Di) included 11 *Vglut*2-Cre mice expressing GCaMP8f (8 males, 3 females) and one female Rosa-lSlL-hM4Di mouse but Tg(*Smim32*-Cre) negative. No significant effect of treatment was observed (repeated-measures ANOVA: *F*_(2,33)_ = 0.10, P = 0.90). Similarly, for TSWM, the hM4Di group included 6 *Vglut2*-Cre and 5 Tg(*Smim32*-Cre) mice (8 males, 3 females), and the control group composition was identical to that described above. Again, no significant effect of treatment was detected (repeated-measures ANOVA: F_(2,33)_ = 0.35, P = 0.70).

### Optogenetic manipulation

For optogenetic manipulations, both Tg(*Smim32*-Cre)61^*Irod*^ heterozygote and *Smim32*-cre knockin mice were used. Tg(*Smim32*-Cre)61^*Irod*^ mice were divided into two groups for the optogenetic experiments. The control group (EYFP) included four mice (2 males and 2 females) injected with AAV2-Ef1a-DIO-EYFP. The experimental group (Halo/Jaws) consisted of animals injected with either AAV1-Ef1a-DIO-eNpHR3.0-EYFP (2 males and 3 females) or AAV1-CAG-FLEX-rc[Jaws-KGC-GFP-ER2] (1 male and 4 females). 10 *Smim32*-cre knockin mice (5 males and 5 females) were injected with AAV1-Ef1a-DIO-eNpHR3.0-EYFP and used for experiments to track body movements during optogenetic manipulation. The preparation of mice for surgical procedures, virus injections, and headpost implantations is mentioned in the above section. Following viral injections, two optical fibers (200μm diameter, 0.39 NA) with ceramic ferrules were implanted bilaterally, positioned ~300μm above the virus injection sites. Optical fibers with a transmission efficiency greater than 70–80% were selected for implantation. A mixture of dental acrylic cement and cyanoacrylate glue was applied around the ceramic ferrules to provide structural support.

Viral expression was allowed to proceed for at least four weeks before initiating laser illumination. A bifurcated optical fiber bundle (200 µm core, 0.39 NA) was coupled to the implanted optical fibers using ceramic sleeves. Laser power at the end of the external fiber was measured with a laser power meter (Thor Labs, PM100D) and adjusted to achieve maximum illumination without raising the local tissue temperature^86^. Illumination was provided with Ce:YAG fiber light source (Doric Lenses Inc., Canada). For EYFP and Halo groups, a 559 nm or 563 nm bandpass filter was used to produce yellow light, while a 612 nm bandpass filter produced orange light for the Jaws group. Illumination was controlled using Doric Neuroscience Studio (Doric Lenses Inc., Canada). Laser power was set to 5-6 mW for the Halo and EYFP groups and to 12 mW for the Jaws group.

In optogenetic inhibition experiments, mice participated in a series of OWM and TSWM sessions with different light-stimulation protocols. Each 100-trial session included an equal number of light-stimulation trials (light) and non-stimulation trials (no-light), arranged in interleaved pseudorandomized blocks of 2–4 trials. In light trials, CLA neurons were inhibited during one of four specific time epochs: (1) during the Sample presentation, (2) for 1 s after the Sample presentation, (3) for 1 s before the Target presentation, or (4) during the Target presentation (Fig. 5e). For *Smim32*-Cre knockin mice, during light trials, CLA neurons were inhibited only for 1 s after the sample cue presentation. Only one of these time epochs was targeted per daily session, and each epoch was repeated across six consecutive sessions before moving to the next. This design ensured sufficient trial counts across all delays for both light and no-light conditions. An exception to this protocol was made for post-Sample light stimulation in the OWM task (first design tested), where light and no-light trials were conducted in separate sessions (three sessions each). This slight adjustment in stimulation strategy did not affect the overall accuracy difference between light and no-light trials (Fig. 5i). To control for visual interference, a masking light of the same wavelength as the stimulation laser was flashed continuously above the animals’ head for both light and no-light trials. The effect of photoinhibition on WM performance was calculated as the relative performance index (%) within a session, using Eq. (8):

8 $$	{Relative}\,{performance}\,{index}\,(\%) \\=	\left(\frac{{Accuracy}\,{in}\,{light}\,{trials-Accuracy}\,{in}\,{no}\,{light}\,{trials}}{{Accuracy}\,{in}\,{no}\,{light}\,{trials}-50}\right)*100$$

To evaluate the effect of photoinhibition on sensory discrimination, a Go/No-Go task was designed for both olfactory (1,4-cineole vs. eugenol) and tactospatial (gentle air puff directed toward either the left or right whisker pad) modalities. Each trial began with a tone, followed by the 1-second sensory cue. The response period coincided with cue presentation and continued for 2 seconds, with a 10-second inter-trial interval. Mice were trained to lick in response to the Go stimulus. In Go trials, a lick during the response period was recorded as a Hit, while no lick was marked as a Miss. In No-Go trials, a lick during the response period was counted as a False Alarm, while no lick was a Correct Rejection. Hits were rewarded immediately with 2–3 µl of water, with no rewards or penalties for other responses. Go and No-Go trials were pseudorandomized, allowing no more than two consecutive trials of the same type. Training continued until mice reached performance levels above 80%. Once trained, mice completed a 120-trial session with interleaved light and no-light trials, balanced evenly across both conditions.

### Body movement analysis

Body movements (pupil, nose, mouth, right forepaw, and ear) were recorded using a CMOS camera (DMK 22AUC03, Imaging Source) equipped with a 2.7-13.5 mm varifocal lens (YV5x2.7R4B-2, Fujinon) at 30 frames per second. The camera was positioned on the right side of head-fixed mice and the operant chamber was illuminated with an infrared LED array (TSHF5410, Vishay). Coordinates and associated confidence likelihoods for each body part were extracted using DeepLabCut (v2.3.10)^87,88^, trained on ~20 frames per mouse sampled before and after the task or during inter-trial intervals. Data points with likelihood ≤ 0.99 were excluded. Regions of interest (ROIs) were then manually defined to remove tracking artifacts. Missing data points were filled through linear interpolation. Movement was quantified as the Euclidean distance between consecutive frames, and these measures were aligned to trial-specific behavioral events for further analysis.

Pupil dynamics were quantified from cropped videos of the eye region. To enhance contrast and improve edge detection, Contrast Limited Adaptive Histogram Equalization (CLAHE) was applied using OpenCV (v4.11.0.86). Pupil contours were then tracked using DeepLabCut, trained on ~20 annotated frames per mouse sampled before and after the task or during inter-trial intervals. Eight equidistant points along the pupil boundary were extracted, and the pupil area was estimated by fitting an ellipse to these points. Tracking data were excluded if fewer than four points had a likelihood >0.5, if any point was identified as an outlier, or if the resulting pupil area deviated markedly from neighboring values. Further, pupil tracking was not performed on mice with partially opened eyelids. During optogenetic sessions, laser delivery through the optical fiber introduced subtle changes in illumination captured by the camera, resulting in fluctuations in luminance and instantaneous, step-like shifts in measured pupil area. These changes did not match the temporal profile of physiological pupil constriction, which typically occurs with a latency of ~0.5 s in mice^89^. Instead, the shifts occurred immediately at light onset and persisted until light offset. The direction of these changes (apparent increases or decreases) varied across mice, likely due to differences in headpost positioning, and across sessions, likely reflecting variations in optical fiber placement. Similar effects were observed regardless of task engagement or whether the optical fiber was connected to the cannula implanted above the CLA, indicating a non-physiological origin. To correct for this artifact, the step-like offset introduced during the entire light stimulation period (from onset to offset) was subtracted from the pupil area traces, enabling accurate tracking of slower pupil dynamics.

To assess whether calcium activity recorded from CLA neurons was temporally coupled to body movements, we performed correlation analyses analogous to those used for licking behavior. This analysis focused on movements of the nose, mouth, right forepaw, ear, and pupil. Because these movements are continuous, we discretized them into binary events. Displacement traces for the nose, mouth, and paw were thresholded at their median values, generating movement events with a temporal resolution of 100 ms. Pupil area signals were first detrended using a wavelet filter, then differentiated and thresholded using the same median-based criterion. The resulting binary movement rasters were downsampled and temporally aligned to the imaging frames within each session. We also assessed whether individual neurons exhibited concurrent temporal coupling with multiple body movements.

### Histology

To validate the viral infections, lens or optic fiber implantations, mice underwent transcardial perfusion with 25 ml of phosphate-buffered saline (PBS) followed by 25 ml of 4% paraformaldehyde (PFA) in PBS. Subsequently, brains were dissected, post-fixed in 4% PFA for 24 h, and then stored in PBS. To visualize the expression patterns, consecutive 50 µm coronal sections were sliced using a vibratome (Leica VT1000S). The tissue sections underwent a washing regimen involving three 15-minute cycles with PBS. Staining with Hoechst (1:5000, Invitrogen, cat no: H3570) was performed for 15 minutes to highlight cell nuclei, followed by another 15-minute wash in PBS. Slices were mounted on slides, embedded in a mounting medium (Vectashield, Reactolab S.A.), and covered by a thin glass coverslip. The samples were imaged using a ZEISS Axiocam multi-channel fluorescence imaging microscope using either a 4X or 10X objective. Typically, the expressed fluorophore exhibited the distinctive ‘peanut’ shape of the CLA, as illustrated in Fig. 5a,d.

### Statistics

Statistical analyses were performed using MATLAB, OriginPro or GraphPad Prism 9.0. The study utilized paired and unpaired t-tests, ANOVA in conjunction with post-hoc multiple comparison tests, the Kolmogorov–Smirnov test, and the Wilcoxon rank-sum test. All tests were two-sided, and unless otherwise specified, error bars in the figures represent the standard error of the mean.

### Reporting summary

Further information on research design is available in the Nature Portfolio Reporting Summary linked to this article.

## Supplementary information

Supplementary Information

Reporting Summary

Transparent Peer Review file

## Data Availability

The data that support the findings of this study are made public at https://doi.org/10.6084/m9.figshare.33093071.

## Code availability

Relevant custom-written MATLAB scripts are made public at https://doi.org/10.6084/m9.figshare.33608527.
