# Supplementary material for: The claustrum contributes to working memory maintenance in mice
Source: Nat Commun. 2026 Oct 14;17:11620. doi: 10.1038/s41467-026-78363-6 (PMC13755406; doi:10.1038/s41467-026-78363-6)
Supplement: Supplementary file 1 [file 41467_2026_78363_MOESM1_ESM.pdf]

Supplementary Information

The claustrum contributes to working memory maintenance in mice

Anindya S. Bhattacharjee[1], Maxime Juventin[1], Chieko Huber[1,2], Burak Unsal[1,2], Jean-Rodolphe Renfer[1,2], Ivan Rodriguez[2,#], and Alan Carleton[1,#]

[1] Department of Basic Neurosciences, Faculty of Medicine, University of Geneva, 1 rue Michel-Servet, 1211 Geneva, Switzerland

[2] Department of Genetics and Evolution, Faculty of Sciences, University of Geneva, quai Ernest-Ansermet, 1211 Geneva, Switzerland

[#] These authors jointly supervised this work.

Correspondence: alan.carleton@unige.ch or ivan.rodriguez@unige.ch

Supplementary Figures 1-5: p. 2-7

Supplementary Table 1: p. 8-32

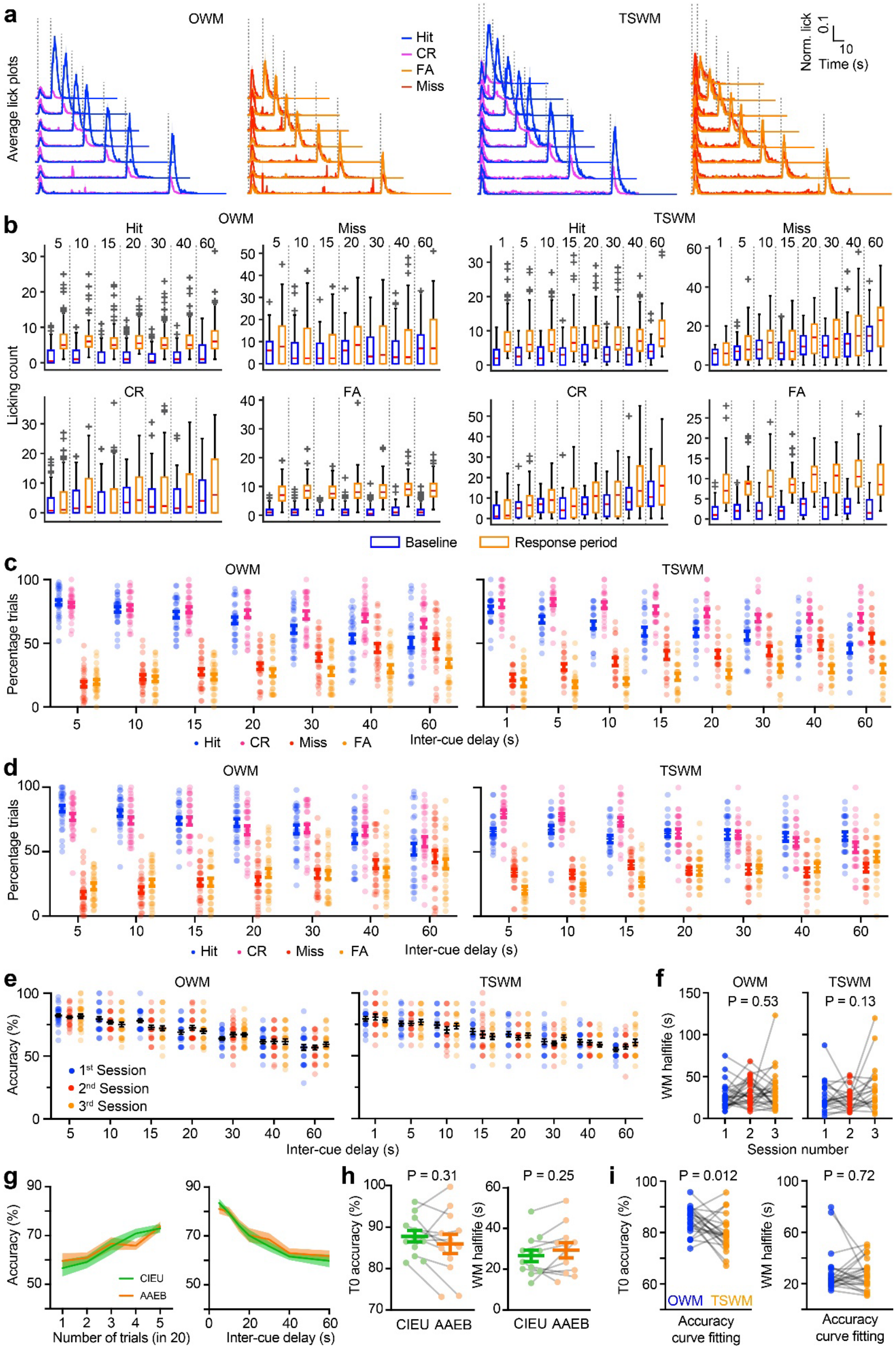
a
OWM
TSWM
Average lick plots
Hit
CR
FA
Miss
Norm. lick 0.1
10
Time (s)
b
OWM
TSWM
Hit
Miss
CR
FA
Licking count
Baseline
Response period
c
OWM
TSWM
Percentage trials
Inter-cue delay (s)
Hit CR Miss FA
d
OWM
TSWM
Percentage trials
Inter-cue delay (s)
Hit CR Miss FA
e
OWM
TSWM
Accuracy (%)
1st Session
2nd Session
3rd Session
Inter-cue delay (s)
f
OWM
TSWM
P = 0.53
P = 0.13
WM halflife (s)
Session number
g
Accuracy (%)
CIEU
AAEB
Number of trials (in 20)
Inter-cue delay (s)
h
P = 0.31
P = 0.25
T0 accuracy (%)
WM halflife (s)
CIEU AAEB
i
P = 0.012
P = 0.72
T0 accuracy (%)
WM halflife (s)
OWM TSWM
Accuracy curve fitting

Supplementary Figure 1. Stability of WM task performance across conditions.
a, PSTHs of normalized lick onset events for each behavioral outcome (Hits, Misses, Correct Rejections, and False Alarms) across inter-cue delays in OWM and TSWM tasks (n = 30 and 23 mice, respectively). Grey dotted lines mark the onset of sample and target cues. b, Lick counts during the baseline and response windows periods for each behavioral outcome (Hits, Misses, Correct Rejections, and False Alarms). Data are shown as box plots representing individual mouse-session measurements, with the median, interquartile range and outliers (gray crosses). Number of animals indicated in a. c, Proportions of behavioral outcomes (Hits, Misses, Correct Rejections, and False Alarms) across inter-cue delays in the OWM and TSWM tasks (number of animals indicated in a), using the trial-classification criterion employed during behavioral testing, in which the pre-response baseline spanned the entire delay period. d, Proportions of behavioral outcomes across inter-cue delays following post hoc reclassification using a fixed 5 s pre-response baseline composed of the first 2.5 s after sample-cue onset and the final 2.5 s immediately preceding the response window (Number of animals indicated in a). e, Session-wise accuracy comparison across delays in OWM and TSWM tasks above. f, Stability of WM performance assessed via session-wise half-life values (RM one-way ANOVA: OWM, $F_{(2,82)} = 0.647$ P = 0.53; TSWM, $F_{(2,61)} = 2.157$ P = 0.13; number of animals indicated in a). g, *Left*: Early learning curves for two odor pairs (CIEU and AAEB) using a fixed 5s inter-cue delay (2WRM ANOVA: $F_{(1,22)} = 0.03$, P = 0.86; n = 12 mice). *Right*: WM performance across inter-cue delays for the same odor pairs (2WRM ANOVA: $F_{(1,22)} = 0.07$, P = 0.80). h, Comparison of WM performance amplitude at 0 delay and half-life for the two odor pairs (2-sided paired t-test). i, Comparison of WM accuracy at 0s delay and the half-life value (parameters derived from exponential curve fitting) for the two WM tasks (2-sided paired *t*-test). Data are presented as mean ± SEM. See Supplementary Table 1 for detailed statistics. Source data are provided as Source Data file.

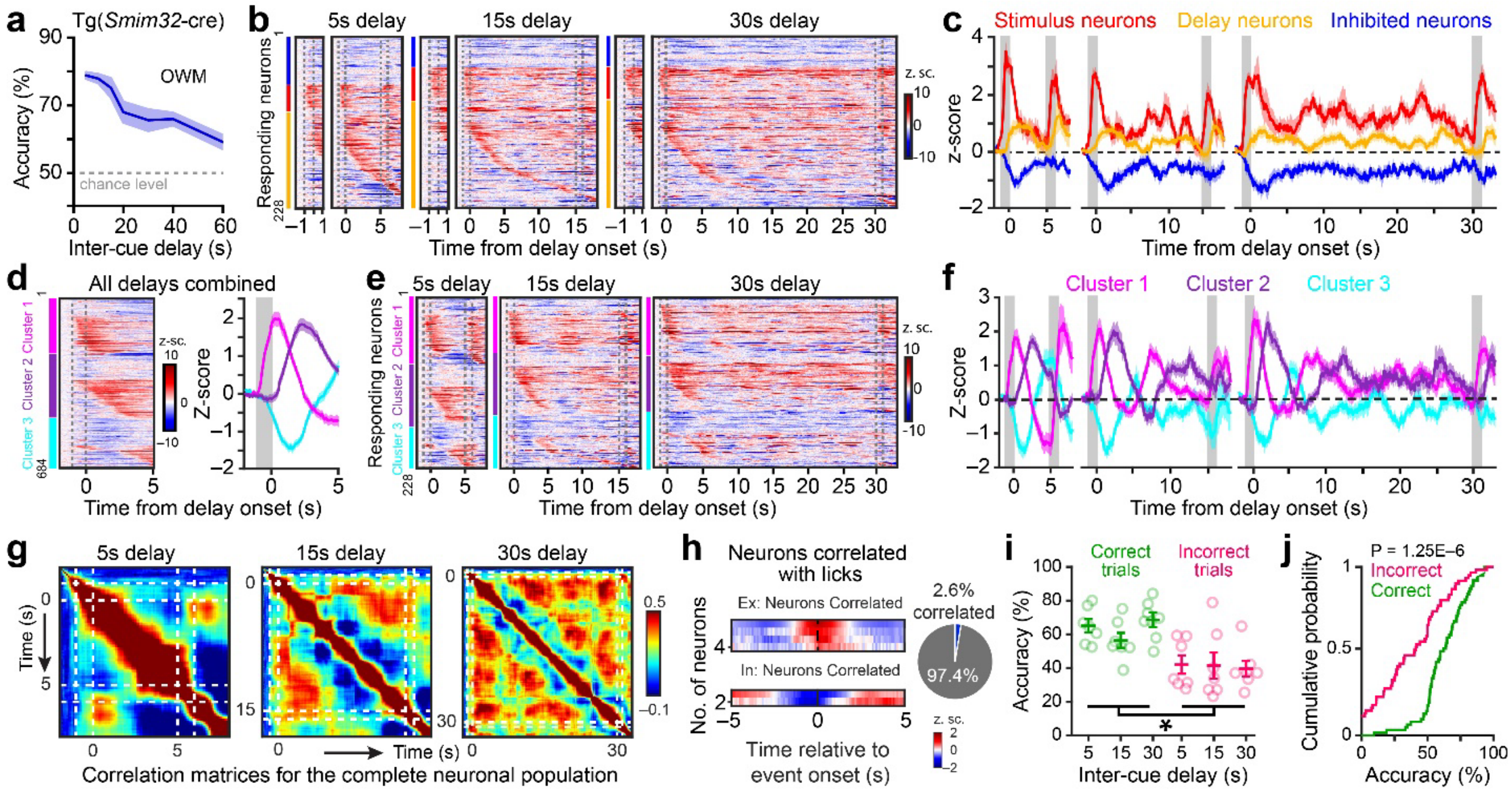

Supplementary Figure 2. Sustained responses of *Smim32*⁺ CLA neurons following cue presentation.
a, WM performance as a function of inter-cue delay duration during imaging sessions (n = 7 mice). b, Pseudo-colored heatmaps showing trial-averaged responses from correct trials for all stimulus-, delay-, and inhibition-responsive neurons (distribution shown by color lines on the left). Neurons pooled from 7 Tg(*Smim32*-cre) mice and sorted by response type and latency (z-score range: –10 to +10). For each delay duration, an expanded zoom including the first second of the delay is shown. Dashed grey lines indicate cue applications. c, Average population calcium activity (mean ± SD). Grey bars mark cue applications. d, Pseudo-colored heatmaps (Left panel) and average population calcium activity (right panel, mean ± SD) for neurons in three clusters identified using k-means (all delay durations used). Neurons (n = 228) pooled from 7 mice and sorted by response latency within each cluster. Responses across all delay durations are shown. Dashed grey lines and the gray box indicate cue timing. e-f, Same as in d, but neural data are plotted separately for each delay duration. g, Correlation matrices showing pairwise correlations of population activity vectors over time (bin size: 100 ms). Dashed white lines mark cue onset. h, Heatmaps of average responses from neurons significantly correlated with lick onset, grouped by excitatory or inhibitory responses. *Right*: Pie chart showing proportion of lick-correlated neurons (blue) relative to the total recorded population. i, Classification accuracy for decoding sample identity during late delay periods in correct and incorrect trials (n = 7 mice, data presented as mean ± SEM; correct vs. incorrect trials: RM ANOVA $F_{(1,6)} = 21.33$, P = 0.0036). j, Cumulative probability distributions of sample cue classification accuracy for correct and incorrect trials (n = 63 animal/delay/session triplets). See supplementary Table 1 for detailed statistics. Source data are provided as Source Data file.

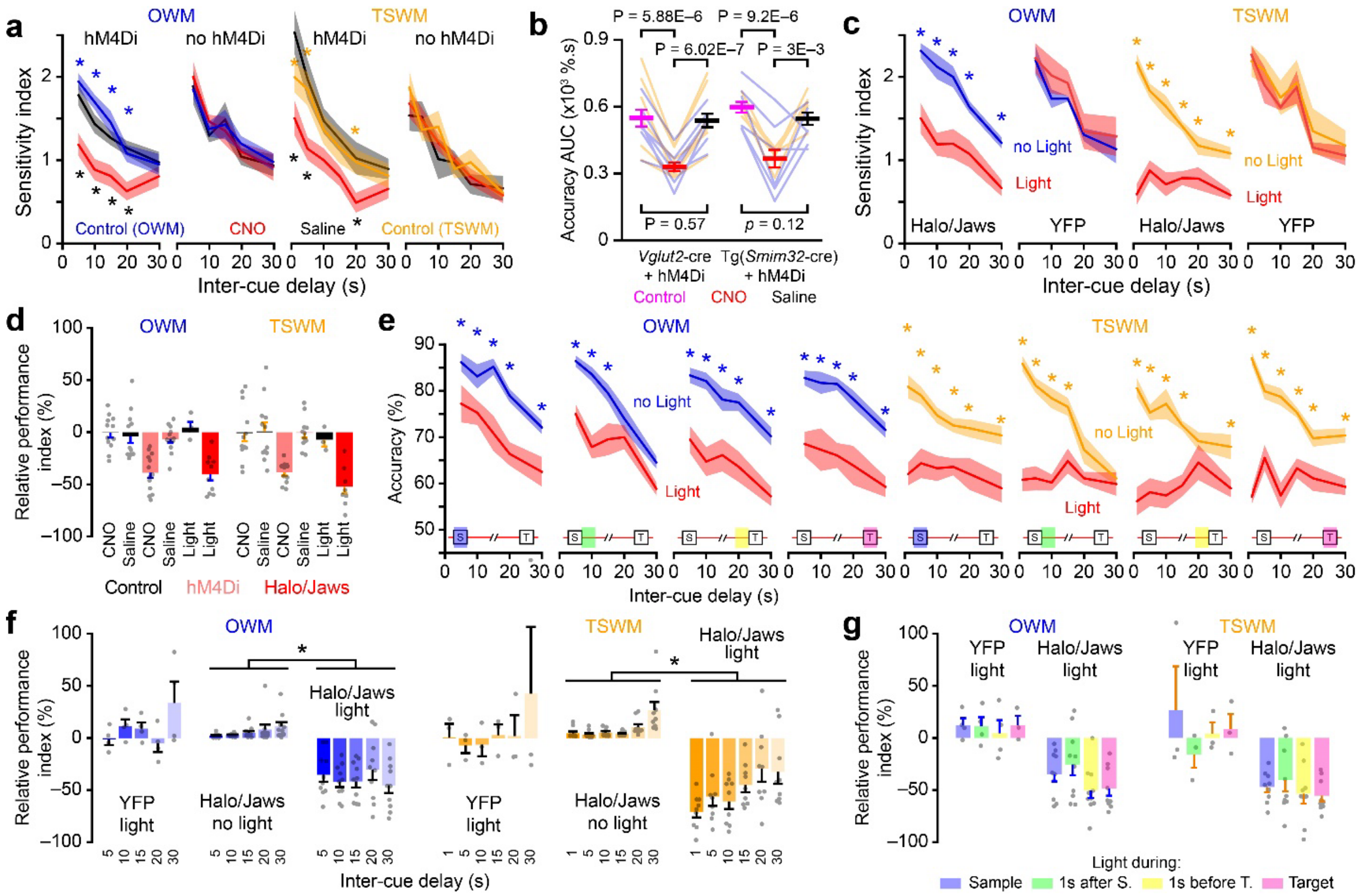

Supplementary Figure 3. Chemo- and optogenetic inhibition of CLA neurons impair working memory performance.
a, WM performances (d' values) plotted as a function of the inter-cue delay for mice expressing hM4Di in the CLA (OWM: n = 13, TSWM: n = 11) and control mice (n = 12 each). Control (no injection), CNO, and saline sessions were performed sequentially (RM ANOVA; post-hoc Fisher's test comparisons *: P < 0.05, significant Control vs. CNO and Saline vs. CNO comparisons are color-coded in blue/orange and black, respectively). b, WM AUC values quantified in two CLA-targeted transgenic lines. Lines represent AUC for individual mice across treatments and tasks (OWM n = 13 and TSWM n = 11, blue and orange, respectively; RM ANOVA with post-hoc Fisher's test comparisons). c, Sensitivity index averaged across four stimulation epochs as a function of inter-cue delay for mice expressing Halo/Jaws or EYFP in the CLA. (Halo/Jaws: OWM n = 10, TSWM n = 9; EYFP: OWM n = 4, TSWM n = 3). No-light and light trials are compared within each group (RM ANOVA; post-hoc Fisher's test *P < 0.05; significant comparisons in blue/orange). d, Relative performance index (relative to control: no CNO or no light) following chemogenetic or optogenetic CLA inhibition or saline injection in OWM and TSWM tasks. Grey dots indicate individual mice. Comparisons were made between CNO and saline (paired t-test *P < 0.05) and between Halo/Jaws and EYFP groups (unpaired t-test *P < 0.05). e, WM performance for individual stimulation epochs as a function of inter-cue delay for Halo/Jaws or EYFP mice. No-light and light trials compared within each group (RM ANOVA; post-hoc Fisher's test P < 0.05; significant comparisons in blue/orange). Epoch schematics shown within each plot. f, Effect of CLA inhibition on WM performance across various inter-cue delays. Relative performance index is calculated for light vs. no-light trials. For Halo/Jaws, no-light trials from each epoch are compared to the average of the other three epochs (2WRM ANOVA: $F_{(1, 80)} = 262.3$ $P = 10^{-15}$ and $F_{(1, 90)} = 217.3$ $P = 10^{-15}$ for OWM and TSWM, respectively). g, Effect of CLA inhibition on WM performance across light epochs for OWM and TSWM tasks in control and experimental groups (One-Way ANOVA between the different light stimulation epochs P > 0.05 for all comparisons). All data represented as mean ± SEM. See Supplementary Table 1 for detailed statistics. Source data are provided as Source Data file.

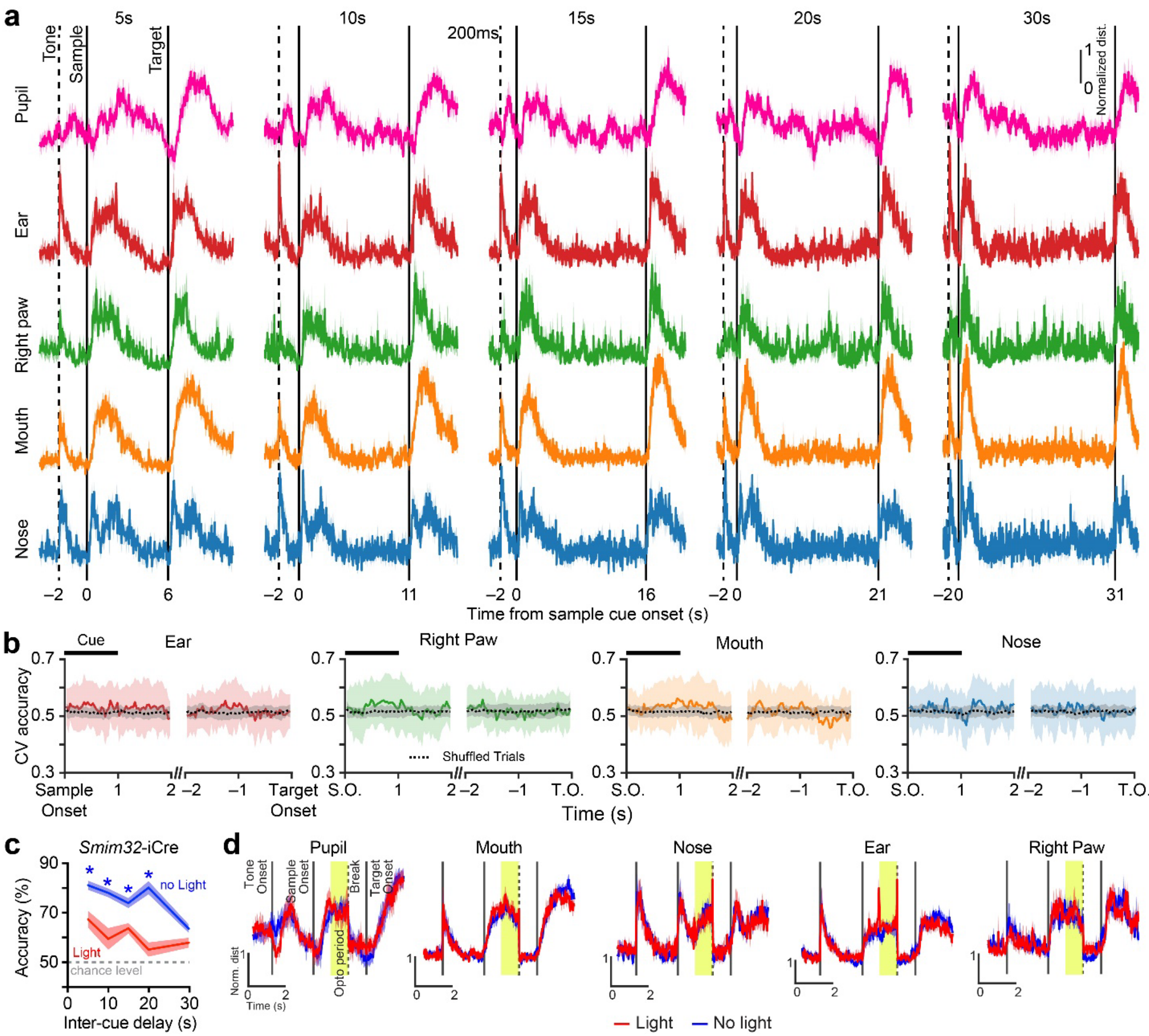

Supplementary Figure 4. Tracking various body movements during WM sessions with optogenetic inhibition of CLA neurons.

a, Normalized pupil area and body-movement traces averaged across mice for all trials with different inter-cue delays (mean ± SEM). Cue onsets (tone, sample, target) are indicated by dashed or solid black lines (For pupil tracking, n = 8 mice (2 mice excluded from analysis due to partially closed eyelids); for other body parts, n = 10 mice). b, Classification accuracy for decoding sample cue identity during the OWM task based on movements of various body parts during cue presentation, and early and late delay epochs (All correct trial durations included; shuffled data in black; shaded areas: classification S.D.). c, WM performance as a function of inter-cue delay in *Smim32*-iCre mice expressing halorhodopsin in the CLA. Light vs. no-light trials are compared (n = 10; RM ANOVA; post-hoc Fisher's test $P < 0.05$). d, Comparison of normalized movement traces (mean ± SEM) for different body parts (pupil, mouth, nose, ear, right paw) between light and no-light trials. To pool data across trials with different inter-cue delays, traces were truncated from 1 s after sample offset to 1 s before target onset. The yellow shaded area marks the optogenetic stimulation window during light trials. See Supplementary Table 1 for detailed statistics. Source data are provided as Source Data file.

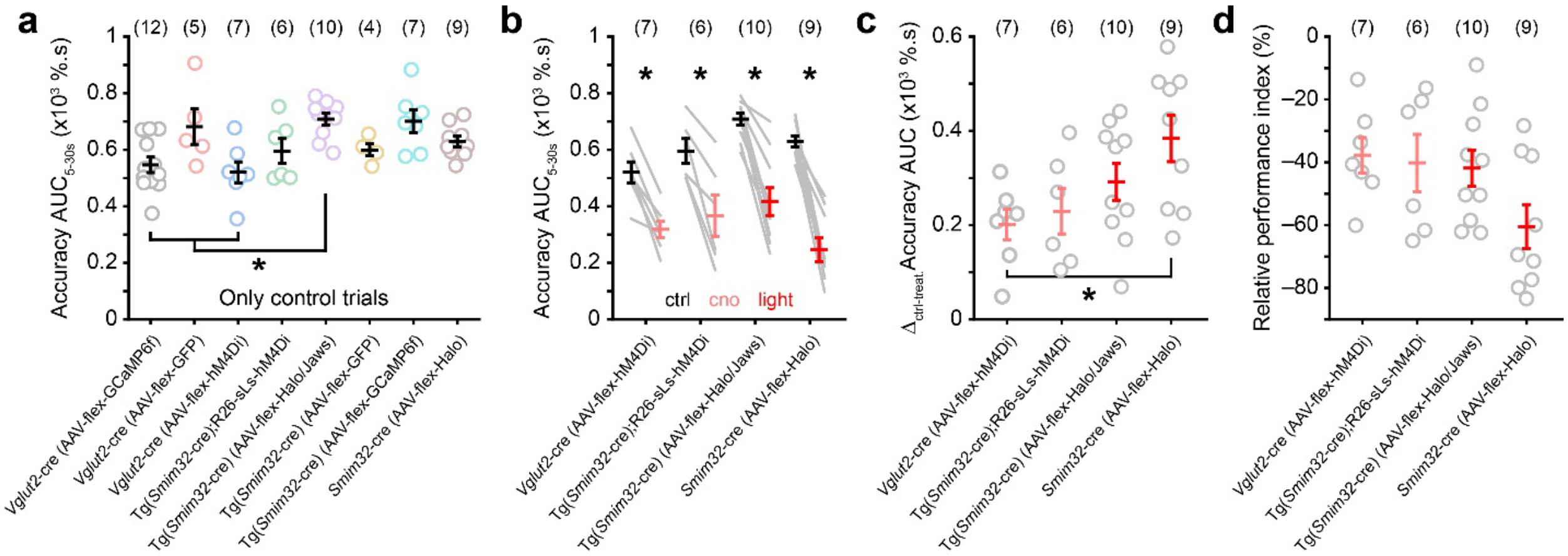

Supplementary Figure 5. Effects of CLA inhibition across optogenetic and chemogenetic manipulations in multiple Cre driver lines.

a, Quantification of overall OWM performance during the first 30 s, measured as the area under the curve (AUC), across the mouse cohorts shown in Fig. 2, Fig. 5b-f, Supplementary fig. 1a, and Supplementary fig. 4c. Only control conditions are included (Brown-Forsythe ANOVA $F_{(7,27.96)} = 4.57$ P =0.0017; post-hoc Dunnett's test *P < 0.05). b, Change in overall OWM performance following chemogenetic or optogenetic CLA inhibition for the different mouse cohorts shown in Fig. 2, Fig. 5b-f, Supplementary fig. 1a, and Supplementary fig. 4c (2WRM ANOVA treatment effect $F_{(3,28)} = 154.3$ P = 10^{-10} & cohort x treatment interaction $F_{(3, 28)} = 154.3$ P = 0.032, post-hoc Bonferroni's test *P = 0.00038). c, Difference in OWM AUC corresponding to the data shown in b (Brown-Forsythe ANOVA $F_{(3,25.22)}$ = 3.59 P = 0.028; post-hoc Dunnett's test *P < 0.05). d, Relative change in performance index (i.e. normalization to individual control conditions) for the data shown in b (Brown-Forsythe ANOVA $F_{(3,22.46)} = 2.44$ P =0.091). Genotype, manipulation type and number of mice are indicated in each panel. Source data are provided as Source Data file.

Supplementary Table 1- detailed statistics

Figure 1

**Panels 1b-c: Comparison: Early trained vs. Trained**		
**Panel 1b: RM ANOVA**	**P value**	**F (DFn, DFd)**
OWM Accuracy	1.0E-15	F (79, 2291) = 4.897
TSWM Accuracy	3.65E-05	F (59, 1298) = 1.942
OWM Sensitivity Index	1.0E-15	F (79, 2291) = 3.903
TSWM Sensitivity Index	1.43E-02	F (59, 1298) = 1.461
**Panel 1c: Paired two-tailed t-test**	**P value**	**t, df**
OWM Accuracy %	2.68E-05	t = 15.79, df = 29
TSWM Accuracy %	8.01E-03	t = 7.190, df = 22
OWM Sensitivity Index	5.77E-05	t = 9.332, df = 29
TSWM Sensitivity Index	3.02E-03	t = 3.332 df = 22
**Panels 1g-I and k-m Comparison: OWM vs. TSWM**		
**RM ANOVA**	**P value**	**F (DFn, DFd)**
**Panel 1g:** Accuracy	1.81E-09	F (1, 357) = 38.12
**Uncorrected Fisher's LSD**	**P value**	**t, df**
5	3.15E-03	2.973, 357
10	1.08E-02	2.563, 357
15	3.54E-05	4.189, 357
20	8.35E-03	2.652, 357
30	6.10E-03	2.759, 357
40	3.31E-01	0.974, 357
60	8.23E-01	0.224, 357
**Panel 1h:** Sensitivity Index	1.1E-07	F (1, 357) = 29.38
**Uncorrected Fisher's LSD**	**P value**	**t, df**
5	0.0042	2.883, 357
10	0.0235	2.276, 357
15	0.0002	3.807, 357
20	0.0673	1.835, 357
30	0.0437	2.024, 357
40	0.3612	0.914, 357
60	0.5484	0.600, 357
**Panel 1i:** Decision Bias	0.0004	F (1, 357) = 12.64
**Uncorrected Fisher's LSD**	**P value**	**t, df**
5	0.0097	2.6, 357
10	0.06	1.887, 357
15	0.1611	1.404, 357
20	0.0912	1.694, 357
30	0.8202	0.227, 357
40	0.7205	0.358, 357
60	0.2176	1.235, 357
**Unpaired two-tailed t-test**	**P value**	**t, df**
**Panel 1k:** T_0 Accuracy	0.0009	t = 3.529, df = 51
**Panel 1l:** T_0 Sensitivity Index	0.0278	t = 2.264, df = 51

**Panel 1m:** Half-life Accuracy	0.4767	t = 0.7169, df = 51
**Panel 1m:** Half-life Sensitivity Index	0.6123	t = 0.5099, df = 51
**Kolmogorov-Smirnov test**		
**Panel 1n: OWM vs. TSWM**	**P value**	**D value**
Half-life Accuracy	0.8259	0.1739
Half-life Sensitivity Index	0.7100	0.1942

Figure 2

**Panel 2d: RM ANOVA (post-hoc Sidak's multiple comparison test)**	**P value**	**t, df**
**OWM**		
Inhibited vs. Stimulus Cells	0.2268	t = 2.085, df = 6
Inhibited vs. Delay Cells	1.22E-05	t = 15.79, df = 6
Stimulus vs. Delay Cells	0.0015	t = 6.81, df = 6
**TSWM**		
Inhibited vs. Stimulus Cells	0.9949	t = 0.226, df = 6
Inhibited vs. Delay Cells	0.0005	t = 8.43, df = 6
Stimulus vs. Delay Cells	0.0003	t = 9.165, df = 6

Figure 4

**RM ANOVA (post-hoc Tukey's multiple comparison test)**		
**Panel 4f: OWM**	**P value**	**F**
Comparison of different time periods	0.24	F (4, 30) = 1.4
**Tukey's multiple comparisons test**	**P value**	**Q, dF**
ED vs. 1/4	0.9283	1.133, 30
ED vs. 1/2	0.9958	0.5236, 30
ED vs. 3/4	0.7516	1.696, 30
ED vs. LD	0.8351	1.47, 30
1/4 vs. 1/2	0.9925	0.6091, 30
1/4 vs. 3/4	0.9944	0.5637, 30
1/4 vs. LD	0.3704	2.603, 30
1/2 vs. 3/4	0.9194	1.173, 30
1/2 vs. LD	0.6265	1.993, 30
3/4 vs. LD	0.1933	3.166, 30
Correct v. shuffled trials	1.0E-15	F (1, 40) = 350.9
**Uncorrected Fisher's LSD**	**P value**	**t, df**
ED	1.0E-15	11.25, 200
1/4	1.0E-15	13.21, 200
1/2	1.0E-15	11.91, 200
3/4	1.0E-15	13.62, 200
LD	1.0E-15	9.406, 200
**Panel 4f: TSWM**	**P value**	**F**
Comparison of different time periods	0.2591	F (4, 30) = 1.3

Tukey's multiple comparisons test	P value	Q, dF
ED vs. 1/4	0.4375	2.433, 30
ED vs. 1/2	0.8084	1.547, 30
ED vs. 3/4	0.7273	1.757, 30
ED vs. LD	0.9995	0.3035, 30
1/4 vs. 1/2	0.9696	0.886, 30
1/4 vs. 3/4	0.9888	0.676, 30
1/4 vs. LD	0.3217	2.736, 30
1/2 vs. 3/4	0.9999	0.21, 30
1/2 vs. LD	0.6883	1.85, 30
3/4 vs. LD	0.5973	2.06, 30
Correct v. shuffled trials	1.0E-15	F (1, 40) = 353.2
**Uncorrected Fisher's LSD**	**P value**	**t, df**
ED	1.0E-15	9.74, 200
1/4	1.0E-15	12.42, 200
1/2	1.0E-15	11.74, 200
3/4	1.0E-15	12.09, 200
LD	1.0E-15	9.182, 200
**Panel 4g: OWM**	**P value**	**F**
Correct v. incorrect trials	0.0077	F (1, 6) = 15.48
Interaction delays x trial type	0.7210	F (1.051, 6.308) = 0.152
	**Adjusted P Value**	**95.00% CI of diff.**
5s: correct v. incorrect trials	0.0019	-7.215 to 42.17
15s: correct v. incorrect trials	0.017	3.596 to 23.16
30s: correct v. incorrect trials	0.059	6.650 to 29.73
**Panel 4g: TSWM**	**P value**	**F**
Correct v. incorrect trials	0.0019	F (1, 6) = 27.71
Interaction delays x trial type	0.0244	F (1.56, 9.36) = 6.147
	**Adjusted P Value**	**95.00% CI of diff.**
5s: correct v. incorrect trials	0.0019	21.68 to 59.61
15s: correct v. incorrect trials	0.017	4.587 to 31.76
30s: correct v. incorrect trials	0.059	-0.7620 to 29.78
**correct trials**		
5s vs. 15s	0.0038	7.046 to 25.18
5s vs. 30s	0.0061	3.170 to 13.43
15s vs. 30s	0.0910	-17.06 to 1.442
**Kolmogorov-Smirnov test**		
**Panel 4h: Comparison**	**P value**	**D value**
5s v. 15s	3.00876E-4	0.45238
5s v. 30s	0.00895	0.35714
15s v. 30s	0.29236	0.21429
Correct v. Incorrect	1E-9	0.41436

Figure 5

RM ANOVA (post-hoc Fisher's LSD)		
**Panel 5b: Performance Accuracy**	**P value**	**F (DFn, DFd)**
OWM Accuracy (hM4Di)	1.18E-05	F (2, 36) = 15.81
**Uncorrected Fisher's LSD**	**P value**	**t, df**
5s		
Control vs. CNO	6.29E-07	5.166, 180
Control vs. Saline	2.85E-01	1.071, 180
CNO vs. Saline	6.37E-05	4.095, 180
10s		
Control vs. CNO	2.08E-07	5.401, 180
Control vs. Saline	5.79E-02	1.908, 180
CNO vs. Saline	6.03E-04	3.492, 180
15s		
Control vs. CNO	3.90E-06	4.764, 180
Control vs. Saline	1.90E-01	1.315, 180
CNO vs. Saline	7.01E-04	3.449, 180
20s		
Control vs. CNO	4.67E-04	3.564, 180
Control vs. Saline	9.68E-01	0.040, 180
CNO vs. Saline	4.05E-04	3.604, 180
30s		
Control vs. CNO	4.67E-01	0.728, 180
Control vs. Saline	9.19E-01	0.102, 180
CNO vs. Saline	5.32E-01	0.626, 180
OWM Accuracy (no hM4Di)	0.9037	F (2, 33) = 0.1016
**Uncorrected Fisher's LSD**	**P value**	**t, df**
5s		
Control vs. CNO	0.5517	5.166, 180
Control vs. Saline	0.9093	1.071, 180
CNO vs. Saline	0.4784	4.095, 180
10s		
Control vs. CNO	0.5626	5.401, 180
Control vs. Saline	0.7277	1.908, 180
CNO vs. Saline	0.3543	3.492, 180
15s		
Control vs. CNO	0.7351	4.764, 180
Control vs. Saline	0.835	1.315, 180
CNO vs. Saline	0.5848	3.449, 180
20s		
Control vs. CNO	0.7587	3.564, 180
Control vs. Saline	0.2445	0.040, 180
CNO vs. Saline	0.3908	3.604, 180

30s		
Control vs. CNO	0.6962	0.728, 180
Control vs. Saline	0.9714	0.102, 180
CNO vs. Saline	0.7229	0.626, 180
TSWM Accuracy (hM4Di)	1.52E-06	F (2, 30) = 21.64
**Uncorrected Fisher's LSD**	**P value**	**t, df**
1s		
Control vs. CNO	6.62E-07	5.155, 180
Control vs. Saline	4.51E-01	0.7562, 180
CNO vs. Saline	1.66E-08	5.912, 180
5s		
Control vs. CNO	1.38E-05	4.469, 180
Control vs. Saline	4.49E-01	0.7588, 180
CNO vs. Saline	2.75E-04	3.711, 180
10s		
Control vs. CNO	5.60E-03	2.804, 180
Control vs. Saline	8.11E-01	0.2391, 180
CNO vs. Saline	1.11E-02	2.565, 180
15s		
Control vs. CNO	2.67E-03	3.045, 180
Control vs. Saline	8.62E-01	0.1741, 180
CNO vs. Saline	1.52E-03	3.22, 180
20s		
Control vs. CNO	4.45E-03	2.88, 180
Control vs. Saline	4.42E-01	0.7705, 180
CNO vs. Saline	3.62E-02	2.11, 180
30s		
Control vs. CNO	3.89E-01	0.864, 180
Control vs. Saline	4.73E-01	0.7185, 180
CNO vs. Saline	1.15E-01	1.582, 180
TSWM Accuracy (no hM4Di)	0.7044	F (2, 33) = 0.3541
**Uncorrected Fisher's LSD**	**P value**	**t, df**
1s		
Control vs. CNO	0.2643	1.119, 198
Control vs. Saline	0.3002	1.039, 198
CNO vs. Saline	0.9358	0.08068, 198
5s		
Control vs. CNO	0.9931	0.008645, 198
Control vs. Saline	0.9501	0.06262, 198
CNO vs. Saline	0.957	0.05398, 198
10s		
Control vs. CNO	0.1726	1.369, 198
Control vs. Saline	0.4633	0.7348, 198
CNO vs. Saline	0.5268	0.6339, 198

15s		
Control vs. CNO	0.5043	0.6689, 198
Control vs. Saline	0.7041	0.3804, 198
CNO vs. Saline	0.2953	1.049, 198
20s		
Control vs. CNO	0.481	0.706, 198
Control vs. Saline	0.7472	0.3227, 198
CNO vs. Saline	0.7019	0.3832, 198
30s		
Control vs. CNO	0.9423	0.07243, 198
Control vs. Saline	0.5827	0.5504, 198
CNO vs. Saline	0.6332	0.4779, 198
**Panel 5c: AUC**	**P value**	**t, df**
OWM (hM4Di)		
Control vs. CNO	1.03E-05	t = 5.123, df = 36
Control vs. Saline	0.3078	t = 1.034, df = 36
CNO vs. Saline	0.0002	t = 4.088, df = 36
OWM (no hM4Di)		
Control vs. CNO	0.9365	t = 0.0802, df = 33
Control vs. Saline	0.6149	t = 0.5079, df = 33
CNO vs. Saline	0.6717	t = 0.4277, df = 33
TSWM (hM4Di)		
Control vs. CNO	1.03E-05	t = 5.288, df = 30
Control vs. Saline	0.785	t = 0.2753, df = 30
CNO vs. Saline	2.25E-05	t = 5.013, df = 30
TSWM (no hM4Di)		
Control vs. CNO	0.566	t = 0.5798, df = 33
Control vs. Saline	0.5107	t = 0.665, df = 33
CNO vs. Saline	0.9326	t = 0.0852, df = 33
**RM ANOVA (post-hoc Fisher's LSD)**		
**Panel 5f: Performance Accuracy**	**P value**	**F (DFn, DFd)**
OWM Accuracy (halo/Jaws)	2.60E-05	F (1, 18) = 31.31
**Uncorrected Fisher's LSD**	**P value**	**t, df**
no light vs. light		
5 s	1.08E-05	4.662, 90
10 s	8.31E-07	5.296, 90
15 s	3.57E-06	4.941, 90
20 s	6.60E-05	4.186, 90
30 s	2.28E-04	3.84, 90
OWM Accuracy (EYFP)	0.3462	F (1, 6) = 1.044
**Uncorrected Fisher's LSD**	**P value**	**t, df**
no light vs. light		
5 s	0.7018	0.3866, 30
10 s	0.3809	0.8894, 30
15 s	0.4035	0.8474, 30
20 s	0.4378	0.7864, 30

30 s	0.0709	1.873, 30
TSWM Accuracy (halo/Jaws)	2.93E-08	F (1, 16) = 99.07
**Uncorrected Fisher's LSD**	**P value**	**t, df**
no light vs. light<br>1 s<br>5 s<br>10 s<br>15 s<br>20 s<br>30 s	 1.0E-15<br>6.48E-12<br>5.38E-13<br>5.02E-07<br>0.000807<br>0.000205	 11.58, 96<br>7.831, 96<br>8.34, 96<br>5.39, 96<br>3.46, 96<br>3.861, 96
TSWM Accuracy (EYFP)	0.3080	F (1, 4) = 1.362
**Uncorrected Fisher's LSD**	**P value**	**t, df**
no light vs. light<br>1 s<br>5 s<br>10 s<br>15 s<br>20 s<br>30 s	 0.756<br>0.2139<br>0.3552<br>0.6979<br>0.6416<br>0.4863	 0.3143, 24<br>1.277, 24<br>0.9428, 24<br>0.3928, 24<br>0.4714, 24<br>0.7071, 24
**Panel 5g: Unpaired two-tailed t-test**	**P value**	**t, df**
RPI: OWM Tom vs hM4Di	1.05E-05	t = 5.604, df = 23
RPI: OWM YFP vs Halo/Jaws	0.0007	t = 4.535, df = 12
RPI: TSWM Tom vs hM4Di	0.0003	t = 4.330, df = 21
RPI: TSWM YFP vs Halo/Jaws	0.0028	t = 3.936, df = 10
**Panel 5h: RM ANOVA**	**P value**	**F (DFn, DFd)**
RPI: effect between delays (OWM)	0.3065	F (2.108, 18.97) = 1.265
RPI: effect between delays (TSWM)	0.0093	F (8, 40) = 5.033
**Tukey's posthoc test**	**P value**	**95% CI of diff.**
OWM		
5s vs. 10s	0.6187	-8.877 to 22.22
5s vs. 15s	0.3386	-4.350 to 17.09
5s vs. 20s	0.9876	-38.71 to 29.08
5s vs. 30s	0.6333	-13.92 to 34.18
10s vs. 15s	>0.9999	-16.83 to 16.23
10s vs. 20s	0.7074	-41.59 to 18.62
10s vs. 30s	0.9748	-16.59 to 23.51
15s vs. 20s	0.7562	-42.73 to 20.37
15s vs. 30s	0.9565	-14.90 to 22.42
20s vs. 30s	0.6748	-22.50 to 52.39
TSWM		
1s vs. 5s	0.7939	-57.21 to 27.73
1s vs. 10s	0.8773	-43.90 to 23.90
1s vs. 15s	0.2162	-62.63 to 11.20
1s vs. 20s	0.1276	-93.19 to 9.935
1s vs. 30s	0.1202	-85.33 to 8.461
5s vs. 10s	0.9863	-23.23 to 32.71
5s vs. 15s	0.4363	-31.47 to 9.518
5s vs. 20s	0.0157	-48.47 to -5.311
5s vs. 30s	0.3065	-61.93 to 14.54
10s vs. 15s	0.2391	-39.00 to 7.568

10s vs. 20s	0.1718	-74.05 to 10.80
10s vs. 30s	0.0254	-53.32 to -3.545
15s vs. 20s	0.4649	-46.55 to 14.73
15s vs. 30s	0.7574	-47.36 to 21.92
20s vs. 30s	0.9999	-49.65 to 56.04
**Panel 5i: RM ANOVA**	**P value**	**F (DFn, DFd)**
RPI: Different Epochs OWM	0.0787	F (3, 26) = 2.536
RPI: Different Epochs TSWM	0.239	F (3, 24) = 1.504
**Panel 5j: Paired two-tailed t-test**	**P value**	**t, df**
ODT EYFP	0.6238	t = 0.5447, df = 3
ODT Halo/Jaws	1.00E+00	t = <0.0001, df = 9
TSDT EYFP	0.4778	t = 0.8659, df = 2
TSDT Halo/Jaws	0.0171	t = 3.000, df = 8
**Panel 5k: Paired two-tailed t-test**	**P value**	**t, df**
ODT vs. OWM	0.0002	t = 5.977, df = 9
TSDT vs. TSWM	8.07E-06	t = 10.07, df = 8

Figure 6

**Panel 6c-f: RM ANOVA**	**P value**	**F (DFn, DFd)**
Hit - hM4Di - OWM	0.0169	F (8, 144) = 2.434
**Uncorrected Fisher's LSD**	**P value**	**t, df**
5s<br>Control vs. CNO<br>Control vs. Saline<br>CNO vs. Saline<br><br>10s<br>Control vs. CNO<br>Control vs. Saline<br>CNO vs. Saline<br><br>15s<br>Control vs. CNO<br>Control vs. Saline<br>CNO vs. Saline<br><br>20s<br>Control vs. CNO<br>Control vs. Saline<br>CNO vs. Saline<br><br>30s<br>Control vs. CNO<br>Control vs. Saline<br>CNO vs. Saline	 0.0182<br>0.4482<br>0.1065<br><br><br>0.0178<br>0.6043<br>0.0627<br><br><br>0.0268<br>0.4748<br>0.1311<br><br><br>0.3045<br>0.356<br>0.0521<br><br><br>0.8993<br>0.4342<br>0.3638	 2.382, 180<br>0.7602, 180<br>1.622, 180<br><br><br>2.392, 180<br>0.5192, 180<br>1.873, 180<br><br><br>2.233, 180<br>0.7162, 180<br>1.517, 180<br><br><br>1.030, 180<br>0.9254, 180<br>1.955, 180<br><br><br>0.1267, 180<br>0.7838, 180<br>0.9105, 180
FA - hM4Di - OWM	0.3062	F (8, 144) = 1.195
**Uncorrected Fisher's LSD**	**P value**	**t, df**
5s<br>Control vs. CNO	 0.1636	 1.399, 180

Control vs. Saline	0.7486	0.3210, 180
CNO vs. Saline	0.2825	1.078, 180
10s		
Control vs. CNO	0.1212	1.557, 180
Control vs. Saline	0.4216	0.8055, 180
CNO vs. Saline	0.4533	0.7516, 180
15s		
Control vs. CNO	0.211	1.255, 180
Control vs. Saline	0.7342	0.3401, 180
CNO vs. Saline	0.3613	0.9152, 180
20s		
Control vs. CNO	0.1212	1.557, 180
Control vs. Saline	0.3292	0.9784, 180
CNO vs. Saline	0.5636	0.5786, 180
30s		
Control vs. CNO	0.6905	0.3988, 180
Control vs. Saline	0.4565	0.7462, 180
CNO vs. Saline	0.7287	0.3473, 180
Hit - no hM4Di - OWM	0.7397	F (8, 132) = 0.6438
**Uncorrected Fisher's LSD**	**P value**	**t, df**
5s		
Control vs. CNO	0.6717	0.4245, 165
Control vs. Saline	0.3864	0.8685, 165
CNO vs. Saline	0.6577	0.4439, 165
10s		
Control vs. CNO	0.9845	0.01944, 165
Control vs. Saline	0.797	0.2576, 165
CNO vs. Saline	0.812	0.2382, 165
15s		
Control vs. CNO	0.4007	0.8425, 165
Control vs. Saline	0.9175	0.1037, 165
CNO vs. Saline	0.4611	0.7388, 165
20s		
Control vs. CNO	0.9752	0.03111, 165
Control vs. Saline	0.2268	1.213, 165
CNO vs. Saline	0.2151	1.244, 165
30s		
Control vs. CNO	0.7561	0.3111, 165
Control vs. Saline	0.6883	0.4018, 165
CNO vs. Saline	0.9278	0.09073, 165
FA - no hM4Di - OWM	0.6701	F (8, 132) = 0.7240
**Uncorrected Fisher's LSD**	**P value**	**t, df**
5s		

Control vs. CNO	0.2763	1.092, 165
Control vs. Saline	0.8868	0.1426, 165
CNO vs. Saline	0.3436	0.9497, 165
10s		
Control vs. CNO	0.523	0.6401, 165
Control vs. Saline	0.9363	0.08001, 165
CNO vs. Saline	0.4725	0.7201, 165
15s		
Control vs. CNO	0.5881	0.5427, 165
Control vs. Saline	0.8676	0.1670, 165
CNO vs. Saline	0.7076	0.3757, 165
20s		
Control vs. CNO	0.718	0.3618, 165
Control vs. Saline	0.8895	0.1392, 165
CNO vs. Saline	0.6171	0.5010, 165
30s		
Control vs. CNO	0.4535	0.7514, 165
Control vs. Saline	0.6392	0.4696, 165
CNO vs. Saline	0.7785	0.2818, 165
Hit - hM4Di - TSWM	0.0833	F (10, 150) = 1.710
**Uncorrected Fisher's LSD**	**P value**	**t, df**
1s		
Control vs. CNO	2.64E-08	5.820, 180
Control vs. Saline	4.39E-01	0.7763, 180
CNO vs. Saline	1.11E-06	5.044, 180
5s		
Control vs. CNO	4.52E-06	4.730, 180
Control vs. Saline	3.09E-01	1.020, 180
CNO vs. Saline	2.76E-04	3.710, 180
10s		
Control vs. CNO	1.38E-03	3.249, 180
Control vs. Saline	8.11E-01	0.2394, 180
CNO vs. Saline	2.99E-03	3.010, 180
15s		
Control vs. CNO	3.16E-04	3.673, 180
Control vs. Saline	4.43E-01	0.7694, 180
CNO vs. Saline	1.55E-05	4.442, 180
20s		
Control vs. CNO	3.16E-02	2.166, 180
Control vs. Saline	7.31E-01	0.3440, 180
CNO vs. Saline	7.01E-02	1.822, 180
30s		
Control vs. CNO	2.60E-02	2.245, 180

Control vs. Saline	6.15E-01	0.5044, 180
CNO vs. Saline	8.34E-02	1.741, 180
FA - hM4Di - TSWM	0.4890	F (10, 150) = 0.9507
**Uncorrected Fisher's LSD**	**P value**	**t, df**
1s		
Control vs. CNO	0.1742	1.364, 180
Control vs. Saline	0.1028	1.640, 180
CNO vs. Saline	0.7833	0.2754, 180
5s		
Control vs. CNO	0.4097	0.8263, 180
Control vs. Saline	0.7002	0.3856, 180
CNO vs. Saline	0.6599	0.4407, 180
10s		
Control vs. CNO	0.4038	0.8368, 180
Control vs. Saline	0.977	0.02886, 180
CNO vs. Saline	0.4202	0.8080, 180
15s		
Control vs. CNO	0.2859	1.070, 180
Control vs. Saline	0.4895	0.6926, 180
CNO vs. Saline	0.0796	1.763, 180
20s		
Control vs. CNO	0.6439	0.4630, 180
Control vs. Saline	0.6973	0.3896, 180
CNO vs. Saline	0.9415	0.07345, 180
30s		
Control vs. CNO	0.0983	1.662, 180
Control vs. Saline	0.1971	1.295, 180
CNO vs. Saline	0.7139	0.3673, 180
Hit - no hM4Di - TSWM	0.6721	F (10, 165) = 0.7546
**Uncorrected Fisher's LSD**	**P value**	**t, df**
1s		
Control vs. CNO	0.0703	1.820, 198
Control vs. Saline	0.3481	0.9405, 198
CNO vs. Saline	0.3805	0.8790, 198
5s		
Control vs. CNO	0.5289	0.6308, 198
Control vs. Saline	0.5718	0.5664, 198
CNO vs. Saline	0.2327	1.197, 198
10s		
Control vs. CNO	0.4429	0.7688, 198
Control vs. Saline	0.2718	1.102, 198
CNO vs. Saline	0.0628	1.871, 198
15s		

Control vs. CNO	0.5768	0.5590, 198
Control vs. Saline	0.5048	0.6682, 198
CNO vs. Saline	0.9131	0.1092, 198
20s		
Control vs. CNO	0.5011	0.6740, 198
Control vs. Saline	0.9633	0.04613, 198
CNO vs. Saline	0.5308	0.6279, 198
30s		
Control vs. CNO	0.9775	0.02819, 198
Control vs. Saline	0.7862	0.2717, 198
CNO vs. Saline	0.7646	0.2998, 198
FA - no hM4Di - TSWM	0.9492	F (10, 165) = 0.3912
**Uncorrected Fisher's LSD**	**P value**	**t, df**
1s		
Control vs. CNO	0.1667	1.388, 198
Control vs. Saline	0.6112	0.5092, 198
CNO vs. Saline	0.3806	0.8788, 198
5s		
Control vs. CNO	0.4671	0.7285, 198
Control vs. Saline	0.8898	0.1388, 198
CNO vs. Saline	0.3868	0.8673, 198
10s		
Control vs. CNO	0.9334	0.0837, 198
Control vs. Saline	0.1821	1.339, 198
CNO vs. Saline	0.1563	1.423, 198
15s		
Control vs. CNO	0.3625	0.9127, 198
Control vs. Saline	0.9057	0.1186, 198
CNO vs. Saline	0.3037	1.031, 198
20s		
Control vs. CNO	0.7955	0.2595, 198
Control vs. Saline	0.7089	0.3739, 198
CNO vs. Saline	0.5273	0.6333, 198
30s		
Control vs. CNO	0.9309	0.0868, 198
Control vs. Saline	0.2967	1.046, 198
CNO vs. Saline	0.3385	0.9594, 198
Hit - Halo/Jaws - OWM	0.0804	F (4, 72) = 2.175
**Uncorrected Fisher's LSD**	**P value**	**t, df**
no light vs. light		
5 s	3.05E-04	3.757, 90
10 s	1.19E-06	5.211, 90
15 s	1.79E-07	5.659, 90
20 s	1.10E-04	4.045, 90

30 s	1.72E-04	3.921, 90
FA - Halo/Jaws - OWM	0.0701	F (4, 72) = 2.269
**Uncorrected Fisher's LSD**	**P value**	**t, df**
no light vs. light		
5 s	0.0411	2.072, 90
10 s	0.4477	0.7627, 90
15 s	0.5266	0.6357, 90
20 s	0.4689	0.7273, 90
30 s	0.7557	0.3120, 90
Hit - EYFP - OWM	0.0585	F (4, 24) = 2.643
**Uncorrected Fisher's LSD**	**P value**	**t, df**
no light vs. light		
5 s	0.9939	0.007665, 30
10 s	0.4747	0.7239, 30
15 s	0.8159	0.2348, 30
20 s	0.5656	0.5810, 30
30 s	0.2172	1.261, 30
FA - EYFP - OWM	0.9221	F (4, 24) = 0.2243
**Uncorrected Fisher's LSD**	**P value**	**t, df**
no light vs. light		
5 s	0.8007	0.2547, 30
10 s	0.8489	0.1921, 30
15 s	0.7706	0.2942, 30
20 s	0.9148	0.1079, 30
30 s	0.8993	0.1276, 30
Hit - Halo/Jaws - TSWM	5.36E-09	F (5, 80) = 12.54
**Uncorrected Fisher's LSD**	**P value**	**t, df**
no light vs. light		
1 s	1.0E-15	9.888, 96
5 s	7.19E-09	6.349, 96
10 s	7.48E-11	7.323, 96
15 s	1.38E-05	4.584, 96
20 s	1.30E-03	3.313, 96
30 s	5.42E-03	2.846, 96
FA - Halo/Jaws - TSWM	0.9492	F (5, 80) = 0.2283
**Uncorrected Fisher's LSD**	**P value**	**t, df**
no light vs. light		
1 s	0.5875	0.5443, 96
5 s	0.7542	0.3140, 96
10 s	0.6278	0.4863, 96
15 s	0.939	0.07679, 96
20 s	0.5609	0.5836, 96
30 s	0.8662	0.1689, 96
Hit - EYFP - TSWM	0.5810	F (5, 20) = 0.7721
**Uncorrected Fisher's LSD**	**P value**	**t, df**
no light vs. light		
1 s	0.514	0.6625, 24
5 s	0.1042	1.689, 24
10 s	0.2471	1.186, 24
15 s	0.7376	0.3390, 24
20 s	0.8549	0.1849, 24

30 s	0.4309	0.8012, 24
FA - EYFP - TSWM	0.4235	F (5, 20) = 1.036
**Uncorrected Fisher's LSD**	**P value**	**t, df**
no light vs. light		
1 s	0.5838	0.5553, 24
5 s	0.547	0.6109, 24
10 s	0.5554	0.5980, 24
15 s	>0.9999	6.15E-10, 24
20 s	0.425	0.8116, 24
30 s	0.704	0.3845, 24
Decision Bias - hM4Di - OWM	0.8005	F (2, 36) = 0.2240
**Uncorrected Fisher's LSD**	**P value**	**t, df**
5s		
Control vs. CNO	0.4169	0.8136, 180
Control vs. Saline	0.9914	0.01078, 180
CNO vs. Saline	0.4108	0.8244, 180
10s		
Control vs. CNO	0.8299	0.2151, 180
Control vs. Saline	0.8582	0.1790, 180
CNO vs. Saline	0.694	0.3941, 180
15s		
Control vs. CNO	0.702	0.3833, 180
Control vs. Saline	0.9765	0.02951, 180
CNO vs. Saline	0.7239	0.3538, 180
20s		
Control vs. CNO	0.6883	0.4019, 180
Control vs. Saline	0.2464	1.163, 180
CNO vs. Saline	0.4476	0.7611, 180
30s		
Control vs. CNO	0.8277	0.2180, 180
Control vs. Saline	0.3828	0.8749, 180
CNO vs. Saline	0.5121	0.6569, 180
Decision Bias – no hM4Di - OWM	0.9603	F (2, 33) = 0.04054
**Uncorrected Fisher's LSD**	**P value**	**t, df**
5s		
Control vs. CNO	0.3902	0.8615, 165
Control vs. Saline	0.7504	0.3186, 165
CNO vs. Saline	0.5879	0.5429, 165
10s		
Control vs. CNO	0.7762	0.2847, 165
Control vs. Saline	0.932	0.08544, 165
CNO vs. Saline	0.8423	0.1992, 165
15s		
Control vs. CNO	0.4637	0.7345, 165
Control vs. Saline	0.9346	0.08215, 165

CNO vs. Saline	0.5151	0.6523, 165
20s		
Control vs. CNO	0.6932	0.3952, 165
Control vs. Saline	0.8367	0.2065, 165
CNO vs. Saline	0.5482	0.6017, 165
30s		
Control vs. CNO	0.6742	0.4212, 165
Control vs. Saline	0.7665	0.2975, 165
CNO vs. Saline	0.9017	0.1237, 165
Decision Bias - hM4Di - TSWM	0.0050	F (2, 30) = 6.366
**Uncorrected Fisher's LSD**	**P value**	**t, df**
1s		
Control vs. CNO	5.92E-05	4.113, 180
Control vs. Saline	1.74E-01	1.366, 180
CNO vs. Saline	6.61E-03	2.748, 180
5s		
Control vs. CNO	2.18E-02	2.314, 180
Control vs. Saline	6.36E-01	0.4746, 180
CNO vs. Saline	6.75E-02	1.839, 180
10s		
Control vs. CNO	5.94E-02	1.897, 180
Control vs. Saline	8.47E-01	0.1938, 180
CNO vs. Saline	9.03E-02	1.703, 180
15s		
Control vs. CNO	1.94E-02	2.359, 180
Control vs. Saline	7.06E-01	0.3785, 180
CNO vs. Saline	6.81E-03	2.738, 180
20s		
Control vs. CNO	5.64E-01	0.5778, 180
Control vs. Saline	7.49E-01	0.3203, 180
CNO vs. Saline	3.70E-01	0.898, 180
30s		
Control vs. CNO	1.10E-01	1.608, 180
Control vs. Saline	5.19E-01	0.6464, 180
CNO vs. Saline	3.38E-01	0.9616, 180
Decision Bias – no hM4Di - TSWM	0.2120	F (2, 33) = 1.626
**Uncorrected Fisher's LSD**	**P value**	**t, df**
1s		
Control vs. CNO	0.0537	1.579, 198
Control vs. Saline	0.6539	0.4490, 198
CNO vs. Saline	0.0687	1.830, 198
5s		
Control vs. CNO	0.252	1.149, 198

Control vs. Saline	0.8968	0.1299, 198
CNO vs. Saline	0.2024	1.279, 198
10s		
Control vs. CNO	0.4627	0.7358, 198
Control vs. Saline	0.3951	0.8522, 198
CNO vs. Saline	0.1139	1.588, 198
15s		
Control vs. CNO	0.3096	1.019, 198
Control vs. Saline	0.6572	0.4444, 198
CNO vs. Saline	0.5665	0.5742, 198
20s		
Control vs. CNO	0.8557	0.1821, 198
Control vs. Saline	0.5665	0.5742, 198
CNO vs. Saline	0.4504	0.7563, 198
30s		
Control vs. CNO	0.9437	0.07067, 198
Control vs. Saline	0.6744	0.4208, 198
CNO vs. Saline	0.6236	0.4915, 198
Decision Bias – Halo/Jaws - OWM	0.0068	F (1, 18) = 9.331
**Uncorrected Fisher's LSD**	**P value**	**t, df**
no light vs. light		
5 s	1.68E-01	1.388, 90
10 s	3.25E-03	3.024, 90
15 s	8.83E-05	4.106, 90
20 s	2.75E-02	2.240, 90
30 s	2.84E-02	2.227, 90
Decision Bias – EYFP - OWM	0.9941	F (1, 6) = <0.0001
**Uncorrected Fisher's LSD**	**P value**	**t, df**
no light vs. light		
5 s	0.897	0.1305, 30
10 s	0.97	0.03786, 30
15 s	0.7602	0.3080, 30
20 s	0.7295	0.3490, 30
30 s	0.6545	0.4521, 30
Decision Bias – Halo/Jaws - TSWM	0.0003	F (1, 16) = 21.67
**Uncorrected Fisher's LSD**	**P value**	**t, df**
no light vs. light		
1 s	1.75E-08	6.153, 96
5 s	2.65E-03	3.087, 96
10 s	3.59E-05	4.336, 96
15 s	1.93E-02	2.380, 96
20 s	5.51E-02	1.942, 96
30 s	1.22E-01	1.560, 96
Decision Bias – EYFP - TSWM	0.5690	F (1, 4) = 0.3840
**Uncorrected Fisher's LSD**	**P value**	**t, df**
no light vs. light		
1 s	0.5061	0.6751, 24

5 s	0.1772	1.390, 24
10 s	0.3731	0.9077, 24
15 s	0.6113	0.5149, 24
20 s	0.6298	0.4883, 24
30 s	0.7543	0.3166, 24
**Panel 6h-i: Simple linear Regression**	**P value**	**R square**
OWM (hM4Di)	0.4093	0.002286
TSWM (hM4Di)	0.3617	0.004247
OWM (Halo/Jaws)	0.0008	0.01096
TSWM (Halo/Jaws)	1.98E-12	0.03755

Supplementary Figure 1

Panel S1f: ANOVA	P value	F (DFn, DFd)
OWM: Stability across 3 sessions	0.5261	F (2, 82) = 0.6474
**Tukey's multiple comparisons test**	**P value**	**Q, dF**
Session 1 vs. Session 2	0.7433	1.04, 82
Session 1 vs. Session 3	0.5041	1.585, 82
Session 2 vs. Session 3	0.9176	0.559, 82
TSWM: Stability across 3 sessions	0.1245	F (2, 61) = 2.157
**Tukey's multiple comparisons test**	**P value**	**Q, dF**
Session 1 vs. Session 2	0.8499	0.7694, 61
Session 1 vs. Session 3	0.306	2.098, 61
Session 2 vs. Session 3	0.1198	2.834, 61
**Panel S1g: 2WRM ANOVA**	**P value**	**F (DFn, DFd)**
CIEU vs. AAEB Learning Acc.	0.8638	F (1, 22) = 0.03014
**Uncorrected Fisher's LSD**	**P value**	**t, df**
CIEU - AAEB		
Block 1	0.4119	0.8237, 110
Block 2	0.6388	0.4707, 110
Block 3	0.7247	0.353, 110
Block 4	0.1608	1.412, 110
Block 5	0.8144	0.2353, 110
CIEU vs. AAEB WM Acc.	0.7954	F (1, 22) = 0.06889
**Uncorrected Fisher's LSD**	**P value**	**t, df**
CIEU - AAEB		
5s	0.4136	0.8197, 154
10s	0.8736	0.1594, 154
15s	0.9963	0.0047, 154
20s	0.9562	0.0549, 154
30s	0.3462	0.9448, 154
40s	0.641	0.4672, 154
60s	0.5271	0.6339, 154
**Panel S1h-i: 2-sided Paired t-test**	**P value**	**t, df**
CIEU vs. AAEB: T_0 Accuracy	0.3074	t = 1.070, df = 11
CIEU vs. AAEB: Halflife Accuracy	0.2507	t = 1.219, df = 10
OWM vs. TSWM: T_0 Accuracy	0.0118	t = 2.744, df = 22

OWM vs. TSWM: Halflife Accuracy	0.7177	t = 0.3662, df = 22

Supplementary Figure 2

**RM ANOVA (post-hoc Tukey's multiple comparison test)**		
**Panel S2i: OWM**	**P value**	**F**
Correct v. incorrect trials	0.0036	F (1, 6) = 21.33
Interaction delays x trial type	0.2744	F (1.452, 8.711) = 1.46
	**Adjusted P Value**	**95.00% CI of diff.**
5s: correct v. incorrect trials	0.0161	6.109 to 40.55
15s: correct v. incorrect trials	0.061	-0.9950 to 32.29
30s: correct v. incorrect trials	0.0032	13.99 to 43.90
**Kolmogorov-Smirnov test**		
**Panel S2j: Comparison**	**P value**	**D value**
Correct v. Incorrect	1.2497E-6	0.47619

Supplementary Figure 3

**RM ANOVA (post-hoc Fisher's LSD)**		
**Panel S3a: Performance Sensitivity Index**	**P value**	**F (DFn, DFd)**
OWM Sensitivity Index (hM4Di)	1.29E-05	F (2, 36) = 15.64
**Uncorrected Fisher's LSD**	**P value**	**t, df**
5s		
Control vs. CNO	6.44E-06	4.649, 180
Control vs. Saline	3.18E-01	1.001, 180
CNO vs. Saline	3.46E-04	3.648, 180
10s		
Control vs. CNO	1.57E-06	4.967, 180
Control vs. Saline	1.10E-01	1.608, 180
CNO vs. Saline	9.56E-04	3.359, 180
15s		
Control vs. CNO	1.15E-04	3.944, 180
Control vs. Saline	2.56E-01	1.139, 180
CNO vs. Saline	5.59E-03	2.805, 180
20s		
Control vs. CNO	6.02E-03	2.78, 180
Control vs. Saline	6.91E-01	0.3984, 180
CNO vs. Saline	1.75E-03	3.178, 180
30s		
Control vs. CNO	3.81E-01	0.8787, 180

Control vs. Saline	8.76E-01	0.1569, 180
CNO vs. Saline	3.02E-01	1.036, 180
OWM Sensitivity Index (no hM4Di)	0.9591	F (2, 33) = 0.04186
**Uncorrected Fisher's LSD**	**P value**	**t, df**
5s		
Control vs. CNO	0.4412	0.7719, 165
Control vs. Saline	0.8529	0.1857, 165
CNO vs. Saline	0.5585	0.5862, 165
10s		
Control vs. CNO	0.6448	0.4619, 165
Control vs. Saline	0.7072	0.3763, 165
CNO vs. Saline	0.4032	0.8382, 165
15s		
Control vs. CNO	0.7129	0.3686, 165
Control vs. Saline	0.7684	0.2949, 165
CNO vs. Saline	0.5079	0.6635, 165
20s		
Control vs. CNO	0.6388	0.4702, 165
Control vs. Saline	0.4361	0.7807, 165
CNO vs. Saline	0.7566	0.3104, 165
30s		
Control vs. CNO	0.7643	0.3003, 165
Control vs. Saline	0.8344	0.2094, 165
CNO vs. Saline	0.9277	0.09094, 165
TSWM Sensitivity Index (hM4Di)	0.0023	F (2, 30) = 7.500
**Uncorrected Fisher's LSD**	**P value**	**t, df**
1s		
Control vs. CNO	4.32E-02	2.036, 180
Control vs. Saline	2.76E-02	2.221, 180
CNO vs. Saline	3.32E-05	4.258, 180
5s		
Control vs. CNO	3.40E-03	2.969, 180
Control vs. Saline	6.24E-01	0.4907, 180
CNO vs. Saline	6.76E-04	3.459, 180
10s		
Control vs. CNO	1.31E-01	1.516, 180
Control vs. Saline	6.62E-01	0.4372, 180
CNO vs. Saline	5.24E-02	1.953, 180

15s		
Control vs. CNO	1.57E-01	1.42, 180
Control vs. Saline	6.73E-01	0.423, 180
CNO vs. Saline	6.70E-02	1.843, 180
20s		
Control vs. CNO	4.40E-02	2.028, 180
Control vs. Saline	8.52E-01	0.1873, 180
CNO vs. Saline	2.80E-02	2.215, 180
30s		
Control vs. CNO	5.15E-01	0.6516, 180
Control vs. Saline	7.34E-01	0.34, 180
CNO vs. Saline	3.23E-01	0.9916, 180
TSWM Sensitivity Index (no hM4Di)	0.7356	F (2, 33) = 0.3099
**Uncorrected Fisher's LSD**	**P value**	**t, df**
1s		
Control vs. CNO	0.3588	0.9197, 198
Control vs. Saline	0.1113	1.6, 198
CNO vs. Saline	0.4973	0.6799, 198
5s		
Control vs. CNO	0.587	0.5441, 198
Control vs. Saline	0.4654	0.7314, 198
CNO vs. Saline	0.8516	0.1873, 198
10s		
Control vs. CNO	0.3661	0.9058, 198
Control vs. Saline	0.0665	1.845, 198
CNO vs. Saline	0.3487	0.9393, 198
15s		
Control vs. CNO	0.5917	0.5373, 198
Control vs. Saline	0.7115	0.3704, 198
CNO vs. Saline	0.8676	0.1669, 198
20s		
Control vs. CNO	0.4067	0.8316, 198
Control vs. Saline	0.214	1.247, 198
CNO vs. Saline	0.6785	0.4151, 198
30s		

Control vs. CNO<br>Control vs. Saline<br>CNO vs. Saline	0.8923<br>0.7926<br>0.6904	0.1356, 198<br>0.2633, 198<br>0.3989, 198
**Panel S3b: RM ANOVA**	**P value**	**t, df**
Vglut2-cre + hM4Di<br>Control vs. CNO<br>Control vs. Saline<br>CNO vs. Saline	 5.88E-06<br>0.5693<br>6.02E-07	 t = 7.655, df = 12<br>t = 0.5852, df = 12<br>t = 9.528, df = 12
Smim32-cre + hM4Di<br>Control vs. CNO<br>Control vs. Saline<br>CNO vs. Saline	 9.20E-06<br>0.1143<br>0.0029	 t = 8.227, df = 10<br>t = 1.73, df = 10<br>t = 3.904, df = 10
**Panel S3c: RM ANOVA**	**P value**	**F (DFn, DFd)**
OWM Halo/Jaws	9.01E-05	F (1, 18) = 25.14
**Uncorrected Fisher's LSD**	**P value**	**t, df**
no light vs. light<br>5 s<br>10 s<br>15 s<br>20 s<br>30 s	 1.55E-05<br>5.95E-06<br>3.91E-05<br>1.76E-03<br>3.10E-03	 4.57, 90<br>4.813, 90<br>4.327, 90<br>3.224, 90<br>3.04, 90
OWM EYFP	0.4659	F (1, 6) = 0.6060
**Uncorrected Fisher's LSD**	**P value**	**t, df**
no light vs. light<br>5 s<br>10 s<br>15 s<br>20 s<br>30 s	 0.8626<br>0.2234<br>0.2817<br>0.874<br>0.3976	 0.1746, 30<br>1.243, 30<br>1.096, 30<br>0.16, 30<br>0.8582, 30
TSWM Halo/Jaws	6.95E-07	F (1, 16) = 61.80
**Uncorrected Fisher's LSD**	**P value**	**t, df**
no light vs. light<br>1 s<br>5 s<br>10 s<br>15 s<br>20 s<br>30 s	 1.0E-15<br>5.79E-09<br>1.20E-08<br>6.55E-05<br>9.74E-03<br>1.18E-03	 10.51, 96<br>6.396, 96<br>6.237, 96<br>4.175, 96<br>2.637, 96<br>3.344, 96
TSWM EYFP	0.5973	F (1, 4) = 0.3284
**Uncorrected Fisher's LSD**	**P value**	**t, df**
no light vs. light<br>1 s<br>5 s<br>10 s<br>15 s<br>20 s	 0.7762<br>0.5038<br>0.6598<br>0.8262<br>0.4539	 0.2875, 24<br>0.6788, 24<br>0.4457, 24<br>0.222, 24<br>0.7613, 24

30 s	0.6449	0.4668, 24
**Panel S3d: Paired two-tailed t-test**	**P value**	**t, df**
OWM no hM4Di: Saline vs. CNO	0.5633	t = 0.5958, df = 11
OWM hM4Di: Saline vs. CNO	0.0003	t = 5.097, df = 12
TSWM no hM4Di: Saline vs. CNO	0.8489	t = 0.1951, df = 11
TSWM hM4Di: Saline vs. CNO	6.53E-06	t = 8.552, df = 10
**Unpaired two-tailed t-test**	**P value**	**t, df**
OWM: Halo/Jaws vs. EYFP	0.0007	t = 4.535, df = 12
TSWM: Halo/Jaws vs. EYFP	0.0028	t = 3.936, df = 10
**Panel S3e: RM ANOVA**	**P value**	**F (DFn, DFd)**
OWM: During Sample	0.0007	F (1, 18) = 16.43
**Uncorrected Fisher's LSD**	**P value**	**t, df**
no light vs. light		
5 s	0.0203	2.363, 90
10 s	0.041	2.073, 90
15 s	0.0003	3.798, 90
20 s	0.0013	3.321, 90
30 s	0.0131	2.532, 90
OWM: 1s After Sample	0.0009	F (1, 18) = 15.91
**Uncorrected Fisher's LSD**	**P value**	**t, df**
no light vs. light		
5 s	3.24E-04	3.739, 90
10 s	1.58E-06	5.142, 90
15 s	1.52E-03	3.272, 90
20 s	1.89E-01	1.324, 90
30 s	6.48E-02	1.87, 90
OWM: 1s Before Target	2.33E-05	F (1, 18) = 31.90
**Uncorrected Fisher's LSD**	**P value**	**t, df**
no light vs. light		
5 s	2.16E-04	3.856, 90
10 s	5.81E-06	4.82, 90
15 s	1.24E-03	3.335, 90
20 s	2.16E-04	3.856, 90
30 s	5.12E-04	3.605, 90
OWM: During Target	5.78E-05	F (1, 16) = 29.27
**Uncorrected Fisher's LSD**	**P value**	**t, df**
no light vs. light		
5 s	0.0004	3.713, 80
10 s	0.0003	3.757, 80
15 s	0.0001	4.018, 80
20 s	0.0003	3.737, 80
30 s	0.002	3.195, 80
TSWM: During Sample	1.11E-07	F (1, 16) = 81.57
**Uncorrected Fisher's LSD**	**P value**	**t, df**
no light vs. light		
1 s	3.42E-07	5.48, 96
5 s	4.64E-05	4.267, 96
10 s	1.26E-03	3.324, 96
15 s	1.06E-02	2.605, 96
20 s	4.98E-03	2.875, 96
30 s	1.26E-03	3.324, 96

TSWM: 1s After Sample	1.49E-06	F (1, 16) = 54.85
**Uncorrected Fisher's LSD**	**P value**	**t, df**
no light vs. light		
1 s	1.28E-12	8.164, 96
5 s	2.83E-09	6.551, 96
10 s	4.42E-08	5.947, 96
15 s	2.29E-04	3.83, 96
20 s	4.66E-02	2.016, 96
30 s	6.88E-01	0.4032, 96
TSWM: 1s Before Target	2.86E-06	F (1, 16) = 49.36
**Uncorrected Fisher's LSD**	**P value**	**t, df**
no light vs. light		
1 s	1.27E-09	6.724, 96
5 s	7.26E-06	4.745, 96
10 s	3.94E-07	5.447, 96
15 s	5.51E-04	3.575, 96
20 s	2.05E-01	1.277, 96
30 s	1.53E-02	2.468, 96
TSWM: During Target	2.03E-09	F (1, 16) = 144.2
**Uncorrected Fisher's LSD**	**P value**	**t, df**
no light vs. light		
1 s	1.0E-15	10.67, 96
5 s	1.44E-06	5.141, 96
10 s	2.09E-11	7.588, 96
15 s	4.28E-05	4.289, 96
20 s	2.71E-03	3.079, 96
30 s	1.44E-04	3.959, 96
**Panel S3f: 2WRM ANOVA**	**P value**	**F (DFn, DFd)**
OWM Halo/Jaws: no light vs. light	1.0E-15	F (1, 80) = 262.3
TSWM - Halo/Jaws: no light vs. light	1.0E-15	F (1, 90) = 217.3
**Panel S3g: RM ANOVA**	**P value**	**F (DFn, DFd)**
OWM - EYFP light	0.9203	F (3, 11) = 0.1611
OWM - Halo/Jaws light	0.1122	F (3, 35) = 2.145
TSWM - EYFP light	0.6641	F (3, 8) = 0.5467
TSWM - Halo/Jaws light	0.5576	F (3, 32) = 0.7024

Supplementary Figure 4

**Panel S4c: RM ANOVA**	**P value**	**F (DFn, DFd)**
Smim32-Cre: Light vs no light	1.17E-13	F (1, 80) = 79.86
**Uncorrected Fisher's LSD**	**P value**	**t, df**
no light vs. light		
5 s	3.34E-04	3.749, 80
10 s	2.39E-06	5.083, 80
15 s	6.73E-03	2.782, 80
20 s	1.57E-09	6.82, 80
30 s	1.26E-01	1.548, 80

Supplementary Figure 5

**Panel S5a: Brown-Forsythe ANOVA**	**P value**	**F (DFn, DFd)**
Smim32-Cre: Light vs no light	0.001651	F (7, 27.96) = 4.57
**Dunnett's T3 multiple comparisons test**	**Adjusted P Value**	**95.00% CI**
Vglut2- GCamP vs. Vglut2 ctrl	0.711061360	-453.8 to 183.7
Vglut2- GCamP vs. Vglut2 ctrl hM4Di	0.999999945	-149.9 to 202.7
Vglut2- GCamP vs. Tg61;Rosa-Dreadd	0.999175024	-260.2 to 161.2
Vglut2- GCamP vs. Tg61 Opsin Halo/jaws	0.003766030	-283.5 to -40.29
Vglut2- GCamP vs. Tg61 EYFP	0.952484370	-193.8 to 88.09
Vglut2- GCamP vs. Tg61 GCamP	0.142094118	-339.0 to 30.41
Vglut2- GCamP vs. Smim32-iCre Opsin Halo	0.378634090	-202.9 to 36.13
Vglut2 ctrl vs. Vglut2 ctrl hM4Di	0.582898174	-159.6 to 482.5
Vglut2 ctrl vs. Tg61;Rosa-Dreadd	0.992750228	-251.2 to 422.3
Vglut2 ctrl vs. Tg61 Opsin Halo/jaws	0.999999997	-359.6 to 305.9
Vglut2 ctrl vs. Tg61 EYFP	0.976009884	-255.7 to 420.0
Vglut2 ctrl vs. Tg61 GCamP	>0.999999999	-346.5 to 308.0
Vglut2 ctrl vs. Smim32-iCre Opsin Halo	0.999727848	-278.9 to 382.2
Vglut2 ctrl hM4Di vs. Tg61;Rosa-Dreadd	0.977456423	-304.1 to 152.4
Vglut2 ctrl hM4Di vs. Tg61 Opsin Halo/jaws	0.025680013	-357.5 to -19.09
Vglut2 ctrl hM4Di vs. Tg61 EYFP	0.811816373	-259.4 to 100.9
Vglut2 ctrl hM4Di vs. Tg61 GCamP	0.116141758	-388.9 to 27.53
Vglut2 ctrl hM4Di vs. Smim32-iCre Opsin Halo	0.364243753	-280.8 to 61.25
Tg61;Rosa-Dreadd vs. Tg61 Opsin Halo/jaws	0.526905710	-325.5 to 100.7
Tg61;Rosa-Dreadd vs. Tg61 EYFP	>0.999999999	-335.1 to 125.5
Tg61;Rosa-Dreadd vs. Tg61 GCamP	0.831794405	-244.5 to 176.7
Tg61;Rosa-Dreadd vs. Smim32-iCre Opsin Halo	0.999978779	-24.01 to 242.1
Tg61 Opsin Halo/jaws vs. Tg61 EYFP	0.132610123	-23.37 to 180.4
Tg61 Opsin Halo/jaws vs. Tg61 GCamP	>0.999999999	-290.9 to 88.09
Tg61 Opsin Halo/jaws vs. Smim32-iCre Opsin Halo	0.241673929	-165.3 to 104.3
Tg61 EYFP vs. Tg61 GCamP	0.591612637	-110.0 to 251.8
Tg61 EYFP vs. Smim32-iCre Opsin Halo	0.997845933	
Tg61 GCamP vs. Smim32-iCre Opsin Halo	0.900315389	
**Panel S5b: Brown-Forsythe ANOVA**	**P value**	**F (DFn, DFd)**
Treatment effect	<0.0000000001	F (1, 28) = 154.3
Cohort x treatment effect	0.03129	F (3, 28) = 3.403
**Bonferrroni's Post-hoc test:** Ctrl v. treatment	**Adjusted P value**	**T value**
Vglut2 DREADD	0.0007185360	4.315
Tg61 DREADD	0.0003751366	4.554
Tg61 Opto	0.0000001545	7.473
Smim32-iCre Opto	0.0000000018	9.325
**Panel S5c: Brown-Forsythe ANOVA**	**P value**	**F (DFn, DFd)**
Difference between experimental cohorts	0.027507	F (3, 25.22) = 3.59
**Dunnett's T3 multiple comparisons test**	**Adjusted P Value**	**95.00% CI**
Vglut2 ctrl hM4Di vs. Tg61;Rosa-Dreadd	0.995931	-218.6 to 162.2
Vglut2 ctrl hM4Di vs. Tg61 Opsin Halo/jaws	0.417488	-242.7 to 61.76
Vglut2 ctrl hM4Di vs. Smim32-iCre Opsin Halo	0.046322	-362.6 to -2.434
Tg61;Rosa-Dreadd vs. Tg61 Opsin Halo/jaws	0.886158	-257.9 to 133.3

Tg61;Rosa-Dreadd vs. Smim32-iCre Opsin Halo	0.217817	-368.0 to 59.30
Tg61 Opsin Halo/jaws vs. Smim32-iCre Opsin Halo	0.618244	-279.0 to 94.94
**Panel S5d: Brown-Forsythe ANOVA**	**P value**	**F (DFn, DFd)**
Difference between experimental cohorts	0.0912	F (3, 22.46) = 2.436
