# Supplementary material for: The claustrum contributes to working memory maintenance in mice
Source: Nat Commun. 2026 Oct 14;17:11620. doi: 10.1038/s41467-026-78363-6 (PMC13755406; doi:10.1038/s41467-026-78363-6)
Supplement: Supplementary file 2 [file 41467_2026_78363_MOESM2_ESM.pdf]

nature portfolio

Corresponding author(s): Alan CARLETON

Last updated by author(s): 23/08/2026

Reporting Summary

Nature Portfolio wishes to improve the reproducibility of the work that we publish. This form provides structure for consistency and transparency in reporting. For further information on Nature Portfolio policies, see our Editorial Policies and the Editorial Policy Checklist.

Statistics

For all statistical analyses, confirm that the following items are present in the figure legend, table legend, main text, or Methods section.

n/a	Confirmed	
☐	☒	The exact sample size (n) for each experimental group/condition, given as a discrete number and unit of measurement
☐	☒	A statement on whether measurements were taken from distinct samples or whether the same sample was measured repeatedly
☐	☒	The statistical test(s) used AND whether they are one- or two-sided<br>*Only common tests should be described solely by name; describe more complex techniques in the Methods section.*
☐	☒	A description of all covariates tested
☐	☒	A description of any assumptions or corrections, such as tests of normality and adjustment for multiple comparisons
☐	☒	A full description of the statistical parameters including central tendency (e.g. means) or other basic estimates (e.g. regression coefficient) AND variation (e.g. standard deviation) or associated estimates of uncertainty (e.g. confidence intervals)
☐	☒	For null hypothesis testing, the test statistic (e.g. F, t, r) with confidence intervals, effect sizes, degrees of freedom and P value noted<br>*Give P values as exact values whenever suitable.*
☒	☐	For Bayesian analysis, information on the choice of priors and Markov chain Monte Carlo settings
☒	☐	For hierarchical and complex designs, identification of the appropriate level for tests and full reporting of outcomes
☒	☐	Estimates of effect sizes (e.g. Cohen's d, Pearson's r), indicating how they were calculated

Our web collection on statistics for biologists contains articles on many of the points above.

Software and code

Policy information about availability of computer code

Data collection	Calcium imaging was acquired using Doric Neuroscience Studio (Doric Lenses Inc., Canada).<br>Mouse behaviour was recorded using Labview software (National instruments).
Data analysis	The analyses of the behavior and the calcium imaging were performed using custom-made Matlab (MathWorks Co) -based scripts that will be made available on repositories or upon reasonable request.

For manuscripts utilizing custom algorithms or software that are central to the research but not yet described in published literature, software must be made available to editors and reviewers. We strongly encourage code deposition in a community repository (e.g. GitHub). See the Nature Portfolio guidelines for submitting code & software for further information.

Data

Policy information about availability of data

All manuscripts must include a data availability statement. This statement should provide the following information, where applicable:

- Accession codes, unique identifiers, or web links for publicly available datasets
- A description of any restrictions on data availability
- For clinical datasets or third party data, please ensure that the statement adheres to our policy

Data availability. The data that support the findings of this study will be made public on a repository.

Research involving human participants, their data, or biological material

Policy information about studies with human participants or human data. See also policy information about sex, gender (identity/presentation), and sexual orientation and race, ethnicity and racism.

Reporting on sex and gender	N/A
Reporting on race, ethnicity, or other socially relevant groupings	N/A
Population characteristics	N/A
Recruitment	N/A
Ethics oversight	N/A

Note that full information on the approval of the study protocol must also be provided in the manuscript.

Field-specific reporting

Please select the one below that is the best fit for your research. If you are not sure, read the appropriate sections before making your selection.

[x] Life sciences   [ ] Behavioural & social sciences   [ ] Ecological, evolutionary & environmental sciences

For a reference copy of the document with all sections, see nature.com/documents/nr-reporting-summary-flat.pdf

Life sciences study design

All studies must disclose on these points even when the disclosure is negative.

Sample size	Comparable sample size as in previously published studies was used.
Data exclusions	No specific exclusion criterion was applied except classical histological verification of viral infection.
Replication	The replication of our results are presented in the study and consisted in independent experiments using similar manipulations and yielding comparable phenotypes. Different kind of internal controls are provided.
Randomization	Animal assignment to the various experimental groups was randomized between the littermates within each cage. Thus, each cage contained all the treatments.
Blinding	All genotypes were identical between groups. The virus expressed in each mouse varied, and in most experiments, the experimenter was unaware of which virus was expressed. However, for the optogenetic experiments, the experimenter was not blinded to this information because lasers with different wavelengths had to be used to stimulate the various opsin types.

Reporting for specific materials, systems and methods

We require information from authors about some types of materials, experimental systems and methods used in many studies. Here, indicate whether each material, system or method listed is relevant to your study. If you are not sure if a list item applies to your research, read the appropriate section before selecting a response.

Materials & experimental systems

n/a	Involved in the study	
[x]	[ ]	Antibodies
[x]	[ ]	Eukaryotic cell lines
[x]	[ ]	Palaeontology and archaeology
[ ]	[x]	Animals and other organisms
[x]	[ ]	Clinical data
[x]	[ ]	Dual use research of concern
[x]	[ ]	Plants

Methods

n/a	Involved in the study	
[x]	[ ]	ChIP-seq
[x]	[ ]	Flow cytometry
[x]	[ ]	MRI-based neuroimaging

Animals and other research organisms

Policy information about studies involving animals; ARRIVE guidelines recommended for reporting animal research, and Sex and Gender in Research

Laboratory animals	A total of 61 mice (31 males and 30 females), aged between 3 and 11 months (at the end of some experiments), were included in the study. No sex differences were detected, and data from both sexes were pooled for all analyses. All experiments were conducted using either Slc17a6tm2(cre)Lowl/J heterozygote mice (referred to as Vglut2-cre in the text; the Jackson Laboratory, strain number 016963), Tg(Smim32-cre)61Irod or Smim32-Cre knockin heterozygote mice (14 males and 17 females), or Tg(Smim32-cre)61Irod;Gt (ROSA)26Sortm1(CAG-CHRM4*,-mCitrine)Ute/J double heterozygote mice, which were obtained by crossing Gt(ROSA)26Sortm1 (CAG-CHRM4*,-mCitrine)Ute/J homozygote mice71 (the Jackson Laboratory, strain number 026219) with Tg(Smim32-cre)61Irod. In Vglut2-cre mice, the cre coding sequence does not disrupt the Slc17a6 gene coding sequence since it was placed after the stop codon72. The Smim32-Cre knock-in mouse line was generated by inserting an IRES-Cre recombinase cassette immediately downstream of the endogenous Smim32 stop codon, thereby preserving endogenous Smim32 expression. The Tg(Smim32-Cre) 61Irod transgenic line was generated by introducing a Cre-expressing transgene under the control of the Smim32 regulatory elements using a bacterial artificial chromosome (BAC). A detailed characterization of these mouse lines is available at [49].
Wild animals	The present study did not involve wild animals.
Reporting on sex	Information about the sex is provided in the methods.
Field-collected samples	This study did not involve any samples collected from the field.
Ethics oversight	All experiments adhered to the ethical guidelines outlined in the Swiss Federal Act on Animal Protection and the Swiss Animal Protection Ordinance. The experimental protocols received approval from the University of Geneva and the Geneva state ethics committees (Licenses GE/94/20 and GE368).

Note that full information on the approval of the study protocol must also be provided in the manuscript.

Plants

Seed stocks	N/A
Novel plant genotypes	N/A
Authentication	N/A
