# Supplementary material for: The claustrum contributes to working memory maintenance in mice
Source: Nat Commun. 2026 Oct 14;17:11620. doi: 10.1038/s41467-026-78363-6 (PMC13755406; doi:10.1038/s41467-026-78363-6)
Supplement: Supplementary file 3 [file 41467_2026_78363_MOESM3_ESM.pdf]

nature portfolio

Peer Review File

The claustrum contributes to working memory maintenance in mice

Corresponding Author: Professor Alan Carleton

This file contains all reviewer reports in order by version, followed by all author rebuttals in order by version.

Version 0:

Reviewer comments:

Reviewer #1

(Remarks to the Author)
The authors present two working memory (WM) tasks. In one task a head fixed mouse is presented with one of two odor cues for a 1 second sample period (paired with a 0.2 second tone to indicate this is a sample not target cue), then there's a delay, then there's a target odor cue presented (for 1 second) which is one of the two odors. If the target odor is the other odor (nonmatch), the mouse can lick or increase lick rate in the 2.5 second response window that starts 0.5 seconds after the target cue onset to get a reward. If the target and sample odors match, no reward or punishment is given. The accuracy measures the frequency of correct licking or increased licking to nonmatch combined with correct rejection (nonlicking) to match conditions. For each trial there is a 10 second intertrial interval between trials, and there's a 2 second baseline before the sample cue is presented. In a second task, instead of two odors there are two air puff cues, one being applied to the left whisker pad and the other to the right whisker pad. Animals learn the odor version first, then the tactile version. The authors show that in both tasks the population activity of claustrum (CLA) neurons recorded with miniscope calcium imaging exhibits cue-specific activity in delays up to 30 seconds, and they show that tonic inhibition of CLA neurons via DREADDS or 1 second optogenetic suppression of CLA neurons during any one of four periods (the sample cue, beginning of delay, end of delay, target cue) produces impaired performance. They also perform a control no-delay discrimination experiment in which the animal must discriminate one of two odor or two tactile cues by licking or nonlicking (one of the two cues is rewarded and the other not). There is no drop in accuracy for the discrimination task during optogenetic suppression of CLA, hence ruling out sensory perception deficits as the cause of impaired performance in the WM tasks. The authors conclude that the CLA contains cue-specific persistent activity and is necessary for their WM tasks.

The finding of 20-30 seconds worth of cue-specific delay activity in CLA is nice, as is the finding that inhibiting CLA tonically or transiently (for 1 second periods) impairs WM performance. There are however several concerns that need to be addressed.

1. The authors frame the abstract, introduction and discussion by saying the field believes working memory is distributed and hence their finding of impaired working memory task performance with CLA inactivation conversely shows there is a single key area for working memory. Though they qualify this angle at some point in the discussion, they should revise the angle throughout because this: the view of distributed memory where no single area is necessary is not what the field has been saying, and the CLA is not the first key area for working memory that has been found. They state in the abstract "Rather than residing in a single store, information is believed to be maintained in a distributed network" and "These findings challenge the notion that no single brain region is indispensable for WM storage". They state in the discussion "Our data challenges the idea that no single area contributes specifically to WM storage or that WM content is stored mainly in short-term plasticity states of synapses. If a dedicated storage area exists, then disrupting activity either during cue presentation (the encoding phase) or during any part of the delay (maintenance phase) should lead to significant information loss and behavioral impairment—precisely what we observed with optogenetic silencing of the CLA." On top of distributed working memory not being the current view, I do not see how their result challenges the idea that working memory information exists in multiple areas where each of multiple areas is individually necessary for working memory- numerous previous studies show targeted manipulations of a single area or pathway can impair working memory task performance. Hence the framing is off and I do not follow the logic of the interpretation. What is novel in the present study is the addition of CLA to the list of areas with delay activity and areas where manipulations can impair working memory performance (assuming point 3 is addressed).

2. The authors mention most delay periods are only a few seconds long (lines 240-244: "Most studies concentrate on short

WM delays, typically ranging from 1.5 to 5 seconds, during which sensory cortex activity often remains active and may influence information transfer. In contrast, we systematically examined the effects of chemogenetic and optogenetic manipulations across delays ranging from 1 to 30 seconds, thereby enhancing our understanding of WM maintenance over longer durations."). That is true for most studies but the authors should explicitly mention in the main text there that cue-selective persistent working memory delay activity lasting 15 seconds or more has been shown before- for example going as far back as in Fuster and Jervey 1982. It is nice to see long lasting persistent delay activity in CLA but it has been seen in other areas.

3. In order to understand and interpret their results, the authors need to show key behavioral data, namely the licking data and hit-correct rejection data. This can help clarify the precise nature of the impairment with CLA inactivation (DREADD and optogenetic). By their measure of accuracy, accuracy can decrease if the animal licks less to nonmatch trials (lower hit rate), licks more to match trials (lower correct rejection rate), both or other possible outcomes. Do the inactivation animals start responding more to match as if they cannot withhold a response after the delay? Do the inactivation animals lick constantly during the delay? Hence the authors should show in the main figures the lick rate averaged over all match and non-match trials as a function of time across the whole trial for the original non-inactivation and inactivation experiments. Then the authors should interpret what the lick results mean for understanding what the CLA inactivation is doing, especially so in light of previous work suggesting manipulations affecting CLA increase distractibility. To add to those raw licking numbers, they should show the hit and correct rejection rate for all of the non-inactivation and inactivation experiments. The question would be whether the CLA inactivation results are better explained as disrupting working memory or nonspecifically impairing ability to keep focus on any task during a delay. The control discrimination experiments do not have a delay so may not address the point.

4. The authors' measure of decrease in performance during inactivation ("% change in accuracy", line 676) is nonstandard and confusing. If I understand it correctly, if the no light accuracy was 100% (perfect), and they drop to chance (50%), their measure of drop in performance is -100%, not -50% (which is the standard way of reporting deficits). If the no light accuracy was 70% and drops to 60%, their measure reports this as a -50% change in accuracy instead of -10% (the standard way). I understand part of the rationale for defining % change in accuracy this way (floor effects), but because it is nonstandard it needs to be explained clearly in the main text. For example the authors make a point of highlighting the large performance drop numbers without explicitly explaining their measure (lines 192, 206, 244-248) is different than standard. In lines 244-248 they write "reducing CLA excitability resulted in a significant decline in WM performance, averaging approximately 40-50%. In some cases, performance dropped by as much as 70% for specific delays, with a complete shutdown observed in some individual mice. The impact of these manipulations was notably strong." More, the name for the measure in the text and axis labels should also be changed from "% change in accuracy" (something I would interpret as meaning the standard way) to something like "accuracy change index" to alert readers to the difference.

Reviewer #2

(Remarks to the Author)
Bhattacharjee and colleagues investigate the claustrum's role in working memory using a classic delayed non-match to sample task. They show that chemogenetic and optogenetic silencing of the claustrum during various task epochs impairs performance, supporting a role for the claustrum in the task. Through calcium imaging of glutamatergic claustrocortical projection neurons, they report that claustrum neurons exhibit cue-specific and delay-period activity, which persists at the population level during long delay periods.

There are several limitations of the study. First, there is a lack of statistical justification for classifying neural responses into three types (Fig. 2). Neurons are classified into one of three types based on an arbitrary activity threshold. Furthermore, from a conceptual perspective, why "Stimulus neurons", many of which also show increased activity in the inter-cue interval (Fig. 2c), are not also considered "Delay neurons" is not clear. Second, analyses do not attempt to account for variables that may modulate claustrum neuron activity such as movements (including licking), motor preparation, engagement and so on, shown in published studies to affect claustrum neuron activity. Third, a central conclusion of the study is that "Delay neurons" exhibit a sequential representation of working memory (Fig. 2c), with activity tiling the delay period once the neurons are sorted by response latency. The Mrsic-Flogel group recently showed that similar patterns of sequential activity are generated in a simple discrimination task as well as a delayed non-match to sample task (doi: 10.1038/s41586-022-05014-3), indicating that these signals may not uniquely reflect working memory (see also Fig. 3b). Averaging across these "Delay neurons" will inherently produce sustained changes in population activity. Although whether the claustrum contributes to working memory is an important question, the analyses as presented do not provide convincing evidence for sustained delay-period activity specific to working memory.

More detailed comments are found below.

Figure 1.

As previous studies have shown that some claustrum neurons respond robustly to movement, an analysis of licks over time for sessions corresponding to the recording data (split by different delays) would address some potential behavioral confounds. An analysis of lick rates and responses based on outcome would also be helpful (hit, FA, CR, miss). An analysis of videos of the mice to assess movement (including potentially tongue movements that do not contact the lick port) as well as pupil size as they relate to claustrum activity would strengthen the paper.

How exactly are ‘hit’ trials identified based on the change in lick rate?

Why do mice perform at greater than 60% accuracy on average the first day of training on the full task structure (Fig. 1b and c) rather than chance accuracy expected in untrained mice? And similarly, why does performance plateau at 60% accuracy at long delays instead of dropping to chance? These questions also relate to the justification for the fits (line 499-506) as performance and d’ are not at 50% or 0 even in the first behavioral session.

Related to lines 474-476 - What criteria are used to determine that mice “learned to perform the task” within 200 to 300 trials (Fig. 1b)? What criteria are used to determine that mice were “proficiently trained” to then expose them to the DNMS task with variable delay periods within a single session? The same questions relate to the tactile version of the task (lines 494-496). What criteria were used to demonstrate “mastery” (line 214)?

How did performance vary across the three sessions for each mouse? Did performance vary initially when the odorants were switched in n = 12 mice? Were mice that performed “better” than average at the OWM task also “better” at the TSWM task (Fig. 1e)?

Figure 2.

The paper rests in part on identifying three response types but the statistical justifications for this classification are unclear. Why choose an arbitrary threshold of one to classify neurons rather than principled approaches for identifying clusters (lines 539-545)? It is also unclear what proportion of neurons are not significantly modulated during the task. As neurons that do not cross the threshold are considered inhibited, it seems that unmodulated neurons and statistically significantly inhibited neurons are grouped together.

Controls demonstrating that claustrum neurons are indeed responding to the sensory stimuli rather than to other observable variables are needed. For example, claustrum neurons are modulated during motor responses like licking (doi: 10.1016/j.neuron.2021.11.013; doi: 10.1101/2021.03.23.436687). These prior studies also showed that anterior claustrum neurons respond rarely to sensory stimuli, prompting the question of why the results differ in these experiments. Do animals lick during the first cue presentations? Linear models, for example, assessing the correlation of claustrum activity with multiple variables and their interactions would strengthen the paper.

As noted above, the Mrsic-Flogel group showed that similar patterns of sequential activity are generated in a simple discrimination task as in a delayed non-match-to-sample task (doi: 10.1038/s41586-022-05014-3), indicating these signals may not uniquely reflect working memory. It is not clear from the analyses included that the claustrum has sustained delay-period activity related to working memory.

The experiments included do not demonstrate that “Delay neurons” resemble hippocampal or entorhinal “time cells.” Whether, for example, they encode absolute or relative time is not characterized (doi: 10.1016/j.neuron.2011.07.012).

High ensemble correlation after sample onset may reflect stereotyped behaviors during the delay period rather than working memory per se, particularly as any correlations must be interpreted in light of how these neurons were initially classified. Further, whether the correlations reflect any aspect of the behavior - for example, does ensemble activity differ across correct and incorrect trials of the same delay and does it predict performance - is not clearly addressed.

Does sustained activity at the end of the delay period, which best decodes stimulus identity, predict correct behavioral responses? Conversely, does decayed activity correspond to incorrect trials, suggesting a failure in maintaining working memory-related information?

Figure 3.

What is the relationship between the population of neurons classified as “cue” selective in Fig. 1 and the neurons classified here using stimulus-specificity measured by Wilcoxon rank sum test? Do all three “types” of neurons in Fig. 1 contribute to the cue-selective stimulus neurons in Fig. 3? And in what proportion?

The analysis based on a Wilcoxon rank-sum test assumes differences in activity are due to stimulus specificity, thus it is not clear that the neurons are “tuned” to the stimuli. If the neurons are stimulus-responsive, one might predict that the sensory response would be similar in correct and incorrect trials. Are the results for each cell the same if incorrect trials are analyzed separately? Why were regression-based methods that could account for multiple variables – such as lick rate – not applied?

What is the distribution of neurons with different response types across mice?
It is not clear what Fig. 3e is demonstrating as the template-matching classifier may assign classifications based on the highest correlation, even when correlations are very weak. The analysis also lacks statistical validation (e.g., permutation tests, cross-validation).

Figure 4.

Although the authors do show that stimulus discrimination itself is not disrupted by silencing, alternative interpretations should be considered as silencing of the claustrum may have effects (i.e., on engagement, motivation, pain as shown in recent studies) that may contribute to or account for degraded behavioral performance without affect working memory

encoding specifically.

Which aspects of behavior (hits, FA, CR, misses) are most influenced by CLA silencing? Affecting different aspects of behavior (i.e., hit rate, FA rate) could similarly alter performance measures plotted here.

Discussion.

Alternative interpretations of the data should be more strongly considered, and some claims (i.e., lines 227-228) should be more qualified. For example, whether CLA ensembles represent “working memory engrams” (line 239) or whether it functions as “RAM” (i.e., lines 277-287) has not been shown by the analyses presented.

Methods.

Lines 310-316 –How were any differences among genotypes assessed?

Lines 334-335 – “dexamethasone (4 mg/mL)” – What dose was delivered to the mice? The methods state a concentration. Same for carprofen (1 mg/mL) in line 341.

Line 376 – Were the same coordinates used for GRIN lens implantation as for viral injection but for the DV position? Please specify.

Line 400 – “a 0.2s tone, with a frequency ranging between 3 and 9 kHz depending on the animal”. What was the rationale for presenting different tone frequencies to different animals?

Lines 403-407 and lines 450-451 – What criteria were used to determine the “higher count of licks” required to qualify as a 'Hit' (correct) trial? Was only one lick event during the response window required to classify the trial as the ‘False Alarm’ trial or was some other criterion used? Showing the motor behavior of different mice in different trial types in the supplement would help clarify this point.

Lines 441-443 – Do mice withhold licks for 5 seconds after the tone following only 20 trials of training? Plots provided in the supplement showing the licking behavior of several example mice at different training stages would clarify the methods.

Lines 477-478 – The description of how the trial types were organized (i.e., delay times, match and non-match trials) in each behavioral session is not clear. Similarly, for lines 662-665.

Line 604 – “DREADD in approximately 50% of the entire CLA population”. Please provide quantification data to support this conclusion.

Line 630 – 11 Vglut2-Cre mice? The total does not add up here.

Reviewer #3

(Remarks to the Author)
I co-reviewed this manuscript with one of the reviewers who provided the listed reports. This is part of the Nature Communications initiative to facilitate training in peer review and to provide appropriate recognition for Early Career Researchers who co-review manuscripts.

Version 1:

Reviewer comments:

Reviewer #1

(Remarks to the Author)
The authors have addressed my comments on the original manuscript to my satisfaction.

However, in one place, they have added new analysis which they claim shows that cue identity was maintained throughout the delay:

"Classifier performance was evaluated during two epochs: the early delay (ED; defined as the first second after the sample period) and the late delay (LD; the final second preceding target presentation). Classifier performance during both ED and LD periods was comparable and consistently exceeded chance levels (i.e., relative to shuffled data), indicating that cue identity was retained throughout the delay (Fig. 4f,g)."

This points out that they only tested the classifier on the first and last second of the delay. I believe this alone would not necessarily be enough to conclude that the cue representations were separable throughout the delay, as suggested by the

PCA trajectories in Fig. 4e. Please also test the classifier on (held out) trials for arbitrary moments throughout the delay (including the middle, 1/4, and 3/4 of the way through the delay) and report the results alongside the early and late delay results.

Reviewer #2

(Remarks to the Author)
The authors were very responsive to the reviewer's comments, presenting additional experiments, new analyses and a substantive revision of the text and figures, thus addressing the major concerns raised in the prior reviews. However, the clarifications in the methods related to how behavioral trials are classified raises an additional point.

The authors now clarify that trial outcomes are defined based on the number of licks in a fixed response window (2.5s) relative to the number of licks in a variable pre-response period which can extend from ~5s to ~60s (p. 25). The period over which to accumulate licks to compare with the licks detected in the fixed response window varies across the delay lengths (short to long), raising the possibility that trials are classified differently for shorter and longer delays. For example, in Supplementary Figure 1a, it seems that fewer licks during the fixed response window are allowed to call a trial a CR trial for shorter delays than for longer delays (compare amplitudes of top pink and bottom pink traces in OWM and TSWM plots). Similarly, more licks may be required to call a long delay trial a FA trial relative to the number for short delay trials, and trials may fail to be classified as hits not due to impaired memory per se, but because mice are required to generate a larger number of licks within the 2.5s response window for long trial types (with longer pre-response periods) than for short trial types. Supplementary Figure 1b also suggests such effects: at short trial durations, hits and CRs are about the same percentage as are misses and FAs; however, as trial duration gets longer, the proportions change with fewer hits and more CRs as well as more misses and fewer FAs. Determining whether and to what extent these differences in pre-response period lengths relative to the fixed response window contribute to the classification of trial outcomes is central to many points in the paper. Does using a fixed 5 s pre-response period (like the period just prior to the response window) affect the trial classification (hit, FA, CA, miss)?

Further, in addition to plotting normalized average lick traces, giving the absolute values and ranges (i.e. a measure of central tendency - median lick count - and variability - IQR) for the number of licks in the response and pre-response windows across delay conditions and trial-outcome types further clarify the results.

Minor comments:

Lines 435-436, 856-858: What is the difference between Tg(Smim32-Cre)61lrod heterozygotes and Smim32-Cre knock-in mice? Are the latter homozygous and the former hemizygous for the transgene?

Some minor typos: Line 462: "0.9jM NaCl" Line 599-600: sensitivity Index Line 848: one Rosa-ISL-hM4Di mouse

Open Access This Peer Review File is licensed under a Creative Commons Attribution 4.0 International License, which

permits use, sharing, adaptation, distribution and reproduction in any medium or format, as long as you give appropriate credit to the original author(s) and the source, provide a link to the Creative Commons license, and indicate if changes were made.
In cases where reviewers are anonymous, credit should be given to 'Anonymous Referee' and the source.
The images or other third party material in this Peer Review File are included in the article's Creative Commons license, unless indicated otherwise in a credit line to the material. If material is not included in the article's Creative Commons license and your intended use is not permitted by statutory regulation or exceeds the permitted use, you will need to obtain permission directly from the copyright holder.
To view a copy of this license, visit https://creativecommons.org/licenses/by/4.0/

Detailed response to reviewers' comments

We thank the reviewers for their time and are grateful for their insightful comments, which greatly helped improve the quality and clarity of our manuscript. In response, we performed additional experiments and analyses to address all concerns in detail, as outlined below. These new data further strengthen and support our conclusions, and the manuscript has been extensively revised accordingly.

For clarity, reviewers' comments are shown in black text and our responses in blue. In the revised manuscript, sections that have been substantially modified are highlighted in blue.

REVIEWER COMMENTS

Reviewer #1 (Remarks to the Author):

The authors present two working memory (WM) tasks. In one task a head fixed mouse is presented with one of two odor cues for a 1 second sample period (paired with a 0.2 second tone to indicate this is a sample not target cue), then there's a delay, then there's a target odor cue presented (for 1 second) which is one of the two odors. If the target odor is the other odor (nonmatch), the mouse can lick or increase lick rate in the 2.5 second response window that starts 0.5 seconds after the target cue onset to get a reward. If the target and sample odors match, no reward or punishment is given. The accuracy measures the frequency of correct licking or increased licking to nonmatch combined with correct rejection (nonlicking) to match conditions. For each trial there is a 10 second intertrial interval between trials, and there's a 2 second baseline before the sample cue is presented. In a second task, instead of two odors there are two air puff cues, one being applied to the left whisker pad and the other to the right whisker pad. Animals learn the odor version first, then the tactile version. The authors show that in both tasks the population activity of claustrum (CLA) neurons recorded with miniscope calcium imaging exhibits cue-specific activity in delays up to 30 seconds, and they show that tonic inhibition of CLA neurons via DREADDS or 1 second optogenetic suppression of CLA neurons during any one of four periods (the sample cue, beginning of delay, end of delay, target cue) produces impaired performance. They also perform a control no-delay discrimination experiment in which the animal must discriminate one of two odor or two tactile cues by licking or nonlicking (one of the two cues is rewarded and the other not). There is no drop in accuracy for the discrimination task during optogenetic suppression of CLA, hence ruling out sensory perception deficits as the cause of impaired performance in the WM tasks. The authors conclude that the CLA contains cue-specific persistent activity and is necessary for their WM tasks.

The finding of 20-30 seconds worth of cue-specific delay activity in CLA is nice, as is the finding that inhibiting CLA tonically or transiently (for 1 second periods) impairs WM performance. There are however several concerns that need to be addressed.

1. The authors frame the abstract, introduction and discussion by saying the field believes working memory is distributed and hence their finding of impaired working memory task performance with CLA inactivation conversely shows there is a single key area for working memory. Though they qualify this angle at some point in the discussion, they should revise the angle throughout because this: the view of distributed memory where no single area is

necessary is not what the field has been saying, and the CLA is not the first key area for working memory that has been found. They state in the abstract "Rather than residing in a single store, information is believed to be maintained in a distributed network" and "These findings challenge the notion that no single brain region is indispensable for WM storage". They state in the discussion "Our data challenges the idea that no single area contributes specifically to WM storage or that WM content is stored mainly in short-term plasticity states of synapses. If a dedicated storage area exists, then disrupting activity either during cue presentation (the encoding phase) or during any part of the delay (maintenance phase) should lead to significant information loss and behavioral impairment—precisely what we observed with optogenetic silencing of the CLA." On top of distributed working memory not being the current view, I do not see how their result challenges the idea that working memory information exists in multiple areas where each of multiple areas is individually necessary for working memory- numerous previous studies show targeted manipulations of a single area or pathway can impair working memory task performance. Hence the framing is off and I do not follow the logic of the interpretation. What is novel in the present study is the addition of CLA to the list of areas with delay activity and areas where manipulations can impair working memory performance (assuming point 3 is addressed).

We thank the reviewer for this thoughtful and constructive comment. We fully agree that our original framing did not accurately reflect the current consensus in the field and may have overstated the conceptual advance of our findings. We appreciate the opportunity to clarify this point and have substantially revised the abstract, introduction, and discussion throughout the manuscript to align with a more accurate and balanced view. Specifically, we have removed statements suggesting that our results demonstrate the existence of a single indispensable storage site. We explicitly acknowledge in the introduction and discussion sections that WM is supported by distributed cortical and subcortical networks, and that multiple regions can be individually necessary for WM performance, as demonstrated in numerous prior studies. Our revised framing no longer contrasts our findings against a "no single area is necessary" view, but instead positions them within the established framework of distributed and functionally interdependent WM circuits.

Accordingly, we have reformulated our central interpretation. Rather than proposing the claustrum (CLA) as a unique or central storage site, we now present it as one component of the distributed WM network whose activity is required for optimal performance. The novelty of our study lies in identifying the CLA as a previously underappreciated node that (i) exhibits sustained, cue-specific population dynamics, (ii) supports WM across multiple sensory modalities, and (iii) is required across multiple task phases, including encoding, maintenance, and readout.

Importantly, our results extend prior work not by challenging the distributed nature of WM, but by showing that CLA perturbation produces robust impairments across modalities and epochs, suggesting a broad contribution to WM processing. We also emphasize that the magnitude of impairment depends on both the genetic strategy and the strength of inhibition. At the same time, residual performance under all conditions indicates that other regions continue to contribute, consistent with a distributed architecture.

We have also revised our mechanistic interpretation. Rather than invoking a dedicated "storage buffer," we now describe the CLA more cautiously as an integrative node that contributes to the maintenance and stability of task-relevant population activity within distributed networks. While we retain a brief reference to buffer-like concepts in the context of classical models, we avoid equating the CLA with a centralized storage system.

Finally, we have clarified that our findings are fully compatible with previous studies showing that perturbations of individual regions (e.g., prefrontal, sensory, or motor areas) can impair WM. In this context, our contribution is to add the CLA to the set of regions that exhibit delay activity and are causally involved in WM performance, while highlighting its multimodal engagement and its requirement across task phases.

We are grateful to the reviewer for pointing out this important issue. These revisions have led to a more precise and balanced presentation of our work and have strengthened the conceptual clarity of the manuscript.

2. The authors mention most delay periods are only a few seconds long (lines 240-244: "Most studies concentrate on short WM delays, typically ranging from 1.5 to 5 seconds, during which sensory cortex activity often remains active and may influence information transfer. In contrast, we systematically examined the effects of chemogenetic and optogenetic manipulations across delays ranging from 1 to 30 seconds, thereby enhancing our understanding of WM maintenance over longer durations."). That is true for most studies but the authors should explicitly mention in the main text there that cue-selective persistent working memory delay activity lasting 15 seconds or more has been shown before- for example going as far back as in Fuster and Jervey 1982. It is nice to see long lasting persistent delay activity in CLA but it has been seen in other areas.

We thank the reviewer for this important clarification. We fully agree that long-lasting, cue-selective delay activity extending 15 seconds or more has indeed been reported in earlier seminal studies, such as the work by Fuster and Jervey (1982), and others that followed. In our manuscript, our intent was not to suggest that long delay-period activity is unprecedented. Rather, our goal was to emphasize that the majority of more recent WM studies, particularly those in rodent models, often employ relatively short delays (typically 1.5–5 seconds), where residual sensory activity can overlap with memory maintenance processes and thus confound interpretation. We now recognize that our initial framing may have underemphasized the long-standing evidence for extended delay activity, and we have revised the main text accordingly.

3. In order to understand and interpret their results, the authors need to show key behavioral data, namely the licking data and hit-correct rejection data. This can help clarify the precise nature of the impairment with CLA inactivation (DREADD and optogenetic). By their measure of accuracy, accuracy can decrease if the animal licks less to nonmatch trials (lower hit rate), licks more to match trials (lower correct rejection rate), both or other possible outcomes. Do the inactivation animals start responding more to match as if they cannot withhold a response after the delay? Do the inactivation animals lick constantly during the delay? Hence the authors should show in the main figures the lick rate averaged over all match and non-match trials as a function of time across the whole trial for the original non-inactivation and inactivation experiments. Then the authors should interpret what the lick results mean for understanding what the CLA inactivation is doing, especially so in light of previous work suggesting manipulations affecting CLA increase distractibility. To add to those raw licking numbers, they should show the hit and correct rejection rate for all of the non-inactivation and inactivation experiments. The question would be whether the CLA inactivation results are better explained as disrupting working memory or nonspecifically impairing ability to keep focus on any task during a delay. The control discrimination experiments do not have a delay so may not address the point.

We thank the reviewer for this important and constructive suggestion. To clarify the behavioral mechanisms underlying the WM impairments induced by CLA inactivation, we conducted a

comprehensive analysis of licking behavior across control sessions as well as during chemogenetic and optogenetic manipulations. In addition, we performed further experiments to determine whether the observed accuracy deficits reflect memory-specific disruption or nonspecific changes in motor output, engagement/arousal, or distractibility. The resulting data and analyses are now presented in the revised manuscript (**Fig. 1**, **Fig. 6**, **Supplementary fig. 1** and **Supplementary fig. 4**).

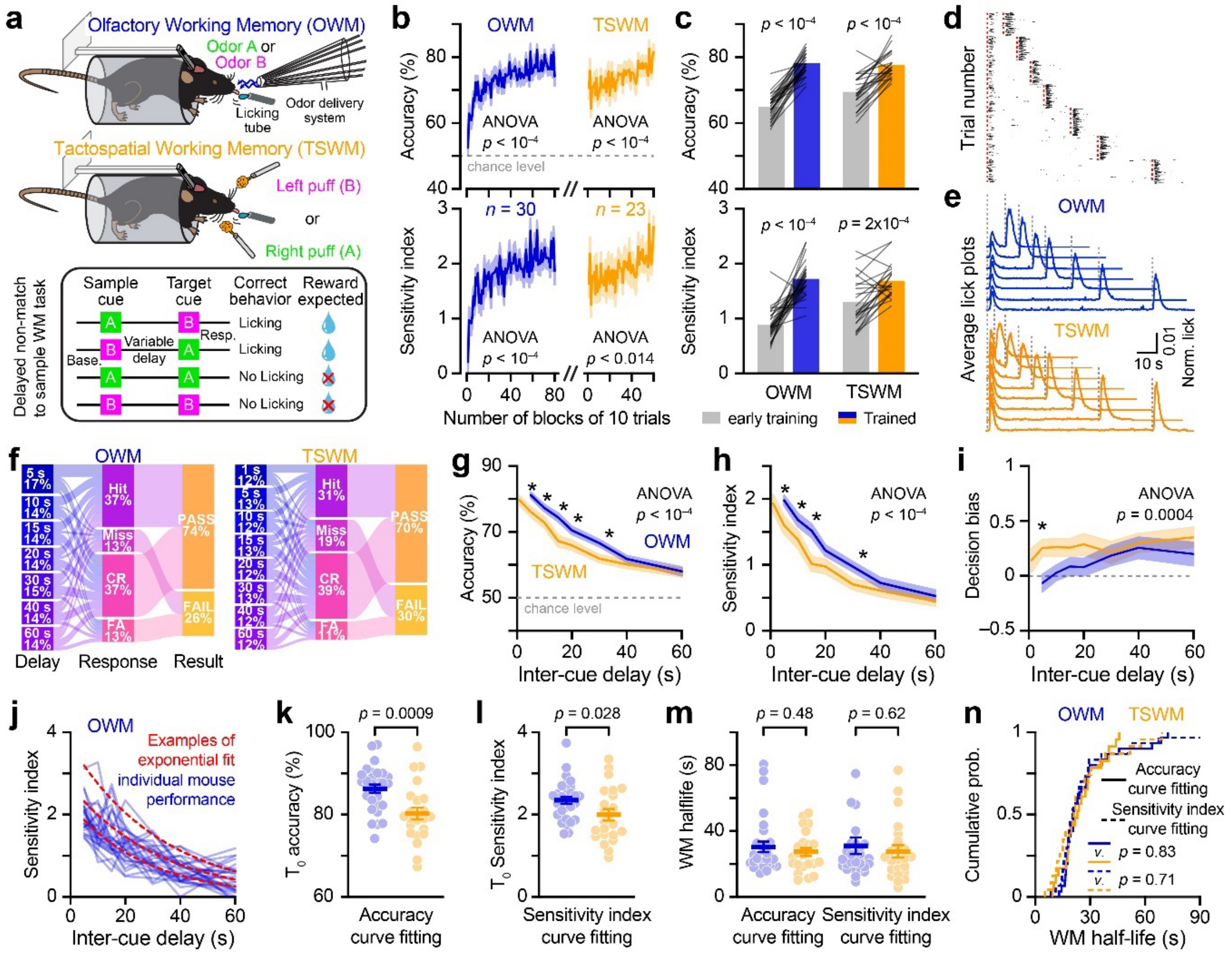

Figure 1. Working memory performance across distinct sensory modalities in mice.
a, Schematic representation of the behavioral paradigm designed to evaluate olfactory and tactospatial WM (OWM and TSWM, respectively) in head-restrained mice. Both sample and target cues were presented for one second. **b**, Learning curves for OWM and TSWM tasks (fixed intercue delay of 5 and 1s, respectively; the number of mice is indicated; one-way repeated measures ANOVA). **c**, Change in performance between the first and the last 100 trials of the training phase shown in **b** for each mouse (black lines; paired t-test) and the mean population performance (colored bars). **d**, Example lick raster from a single mouse performing the OWM task (trials sorted by delay duration). Dotted lines indicate cue onset. **e**, Peri-stimulus time histogram showing normalized lick initiation counts averaged across all mice during OWM and TSWM sessions. **f**, Sankey plots summarizing the distribution of trial outcomes across different delay durations for both OWM and TSWM tasks. CR: correct rejections, FA: false alarms. **g-i**, WM performances plotted as a function of the intercue delay duration (2WRM ANOVA, post-hoc Fisher's test *: P < 0.05). **j**, Variability of WM performance across individual animals shown for sensitivity index computed during the OWM task. Examples of exponential fit (used to calculate the amplitude at 0 delay and the half-life) of a few individual performance curves are illustrated (red dashed lines). **k-m**, Comparison of WM performance amplitude at 0 delay and half-life for OWM and TSWM tasks (unpaired t-test). **n**, Cumulative probability distribution of half-lives for OWM and TSWM tasks (Kolmogorov-Smirnov test). Data in **b**, **e**, **g-i**, **k-m** are presented as mean ± SEM. See Supplementary Table 1 for detailed statistics.

Across experiments, mice consistently initiated licking during the sample cue presentation. This behavior, which arose from the pretraining requirement to obtain a small water reward following cue presentation, was neither discouraged nor punished during later sessions, as it could provide a useful measure of arousal and engagement. While some licking occurred during cue presentation, mice rapidly learned to suppress licking during the inter-cue delay period before responding to the target cue (**Fig. 1d,e**). This behavior was consistent across all delays, indicating sustained task engagement and intact motor control, and demonstrating that mice could withhold licking for up to a minute before responding to the target cue. Notably, lick raster plots revealed distinct temporal licking patterns across trial types, particularly during the response window (**Supplementary fig. 1a**). Hit trials were characterized by elevated licking specifically during the response period, whereas Correct Rejection trials showed suppressed licking. False Alarms and Misses occurred when this suppression failed, or when licking during the response window did not reach the threshold required for a Hit (See also our response to the comment regarding threshold definition in another reviewer's report).

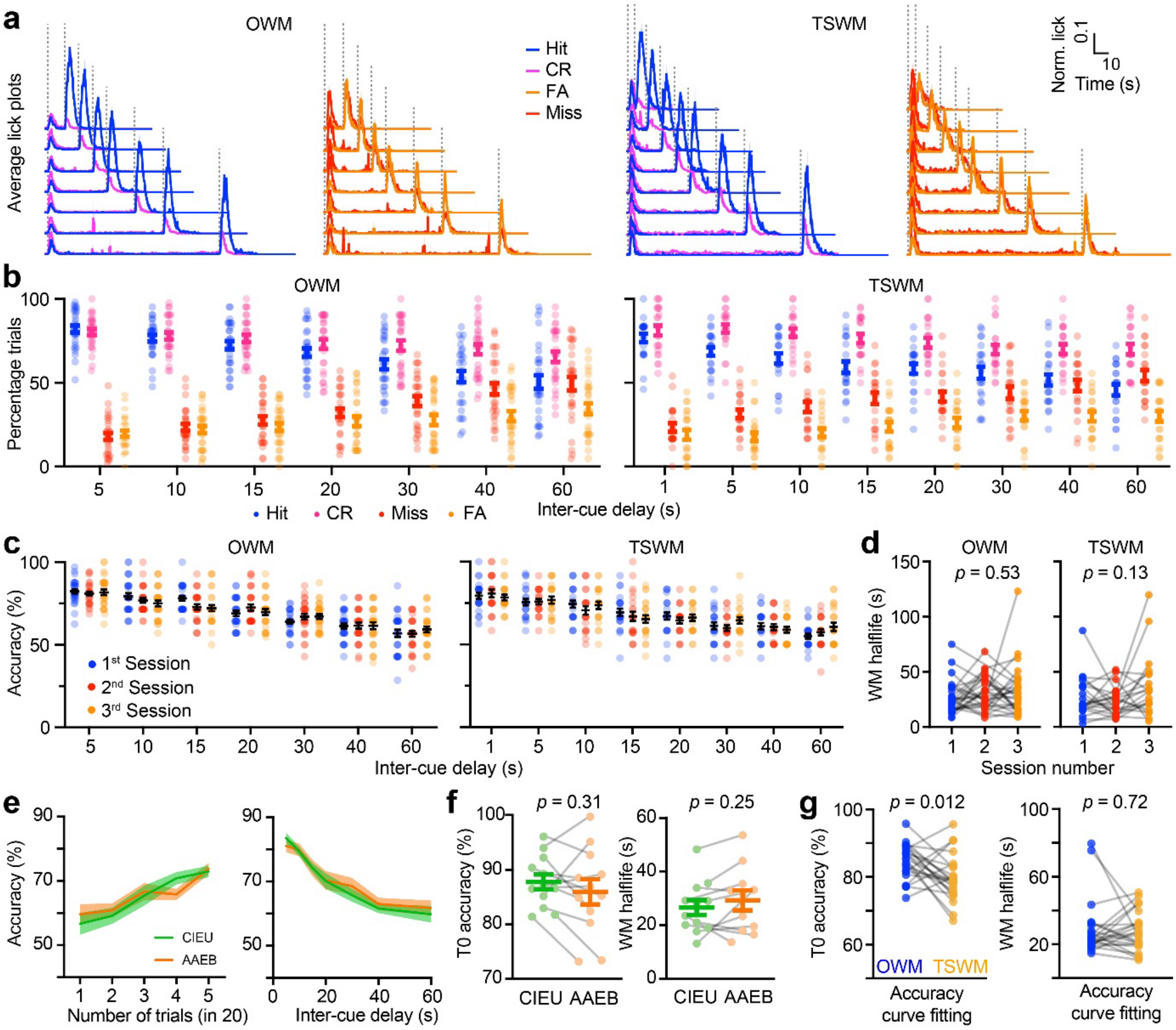

Supplementary Figure 1. Stability of WM task performance across conditions.

a, PSTHs of normalized lick onset events for each behavioral outcome (Hits, Misses, Correct Rejections, and False Alarms) across inter-cue delays in OWM and TSWM tasks. Grey dotted lines mark the onset of sample and target cues. **b,** Proportions of each behavioral response type (hits, misses, correct rejections, and false alarms) across varying inter-cue delays for OWM and TSWM tasks. **c,** Session-wise accuracy comparison across delays in OWM and TSWM tasks. **d,** Stability of WM performance assessed via session-wise half-life values (RM one-

way ANOVA: OWM, $P = 0.53$; TSWM, $P = 0.13$). **e,** *Left*: Early learning curves for two odor pairs (CIEU and AAEB) using a fixed 5s inter-cue delay (2WRM ANOVA: $F_{(1,22)} = 0.03$, $P = 0.86$; $n = 12$). *Right*: WM performance across inter-cue delays for the same odor pairs (2WRM ANOVA: $F_{(1,22)} = 0.07$, $P = 0.80$). **f,** Comparison of WM performance amplitude at 0 delay and half-life for the two odor pairs (Cineol and Eugenol or Amyl acetate and Ethyl butyrate; paired t-test). **g,** Comparison of WM accuracy at 0s delay and the half-life value (parameters derived from exponential curve fitting) for OWM and TSWM tasks (paired *t*-test). Data are presented as mean ± SEM. See **Supplementary Table 1** for detailed statistics.

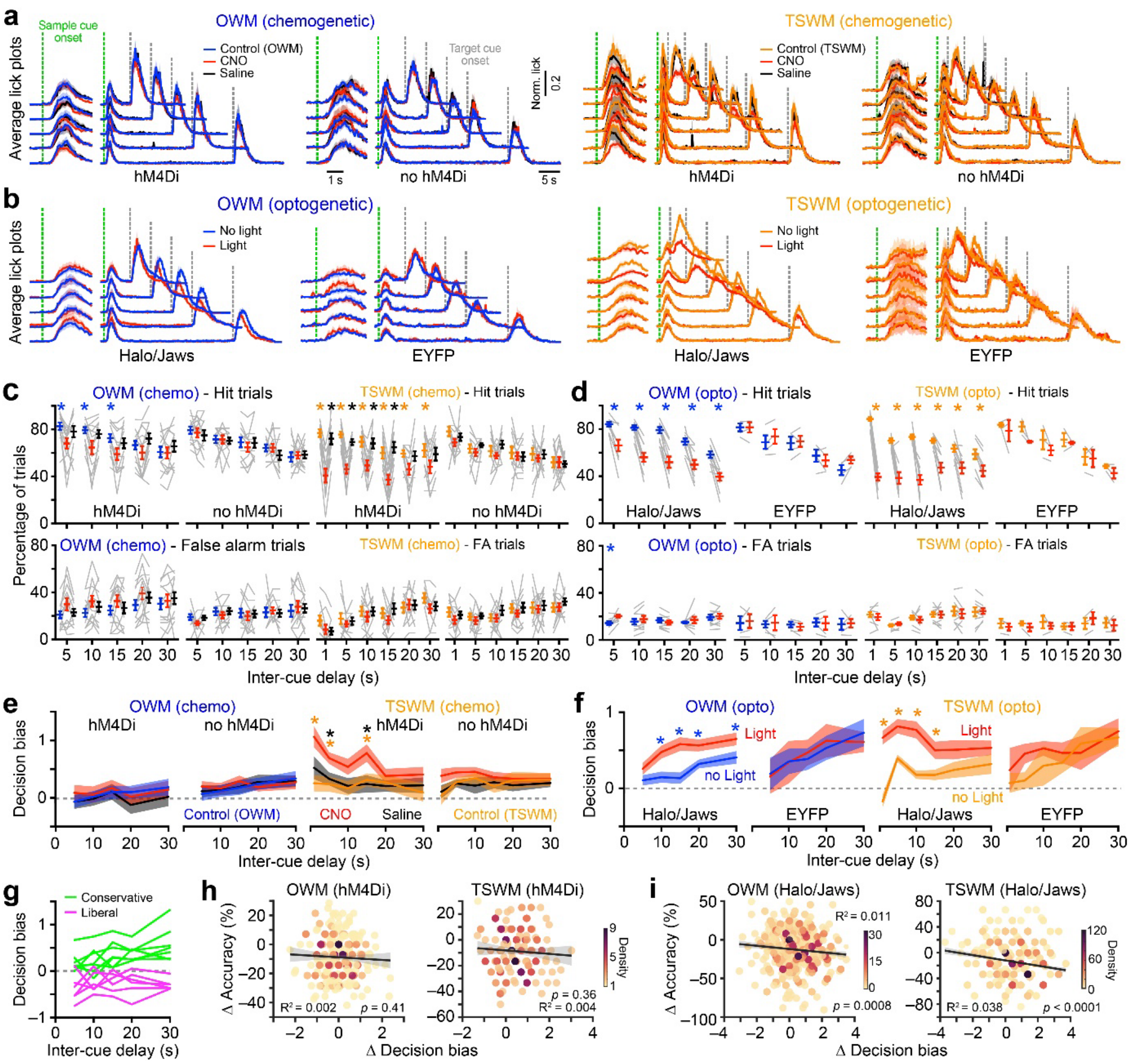

Figure 6. CLA manipulations influence behavioral responses and decision bias.
a-b, PSTH of normalized lick onset events across conditions. Licking after sample cue (*left*) and during full trial (*right*) are shown separately. Green and grey dotted lines mark sample and target onset, respectively. **c,** Percentage of Hit and FA trials during control, CNO, and saline sessions for OWM and TSWM tasks (RM ANOVA; post-hoc Fisher's test comparisons *: $P < 0.05$, significant comparisons for Control vs. CNO and Saline vs. CNO are color-coded in blue/orange and black, respectively). **d,** Percentage of Hit and FA trials during light and no-light trials for both WM tasks (RM ANOVA; post-hoc Fisher's test *$P < 0.05$). **e,** Decision bias vs. inter-cue delay for mice expressing hM4Di (OWM: n = 13, TSWM: n = 11) and controls (n = 12 for both). Sessions included control (no injection), CNO, and saline in order (RM ANOVA; post-hoc Fisher's test *$P < 0.05$; color coding as in **c**). **f,** Decision bias vs. delay duration for mice expressing Halo/Jaws or EYFP. Light vs. no-light trials compared within groups (Halo/Jaws: OWM n = 10, TSWM n = 9; EYFP: OWM n = 4, TSWM n = 3; RM ANOVA; post-hoc Fisher's test *$P < 0.05$). **g,** Individual variability in decision bias (from e) as a function of delay: hM4Di-expressing mice in control OWM sessions showed divergent strategies: conservative (green) vs. liberal (magenta) licking. **h,** Accuracy difference (CNO – control) vs. change in decision bias across delay periods (color indicates point

density). **i**, Accuracy difference (light – no-light) vs. decision bias change across delays during optogenetic stimulation epochs (**Fig. 5e**); color shows point density. Data shown as mean ± SEM. See **Supplementary Table 1** for full statistics.

First, to assess whether CLA inactivation altered general licking behavior or impulse control, we examined trial-averaged licking profiles across the full task duration for both match and non-match trials. Across all conditions, we observed no systematic differences in overall licking rates between inactivation and control sessions (**Fig. 6a,b**), indicating preserved motor execution. Importantly, licking traces showed no changes in responses to the sample cue and no increase in premature or sustained licking during the delay period (including at long delays) under CLA inactivation, arguing against heightened impulsivity, impaired response withholding, or disengagement.

Second, to determine how behavioral accuracy was specifically affected, we separately quantified Hit rates and False Alarm (FA) rates. CLA inactivation consistently reduced Hit rates across both chemogenetic and optogenetic manipulations (**Fig. 6c,d**), indicating impaired correct responding on non-match trials. In contrast, FA rates varied across animals, with some individuals exhibiting increases and others decreases, suggesting heterogeneous shifts in response strategy rather than a uniform failure to withhold responses. To further interpret these effects, we quantified decision bias using signal detection theory metrics (**Fig. 6e–g**). Mice displayed diverse baseline strategies ranging from conservative to liberal responding (**Fig. 6g**), and CLA inactivation induced variable shifts in bias across individuals and conditions. While some animals became more conservative under inactivation, others showed no consistent trend or became more liberal. This heterogeneity argues against a generalized increase in distractibility or impulsive responding as the primary driver of performance deficits. Critically, we tested whether changes in decision bias explained the decline in WM accuracy by correlating session-by-session changes in accuracy with shifts in bias. Across both tasks and both manipulation strategies, no strong correlations were observed (**Fig. 6h,i**). This dissociation indicates that WM impairments cannot be accounted for by altered response criteria, motivation, or general task engagement.

Third, given the widespread reciprocal connectivity of the CLA with cortical and subcortical regions, optogenetic or chemogenetic perturbations may affect distributed networks beyond WM-specific functions. For example, prior work has shown that the claustro-cingulate pathway can exert complex, and in some cases opposing, effects on pain modulation[1, 2, 3]. To address this possibility, we examined whether optogenetic inhibition of CLA neurons induced acute pain, stress-related responses, or gross motor disturbances. In a new cohort of *Smim32*-iCre mice (n = 5 males and 5 females) injected with AAV-EF1a-DIO-eNpHR3.0-EYFP, we quantified body movements and pupil size during light-on and light-off trials, with light delivered for 1 s following the sample cue (**Supplementary Fig. 4**). Although CLA inhibition significantly reduced task accuracy, movement metrics and pupil measurements showed no significant differences between conditions (**Supplementary Fig. 4d**), suggesting that the manipulation did not produce overt distress or motor impairments.

Together, these analyses demonstrate that CLA inactivation selectively disrupts WM-guided decision-making. The disruption was not linked to excessive licking, impaired response withholding, or global attentional deficits. While prior work has suggested that claustral perturbations may influence distractibility in other behavioral contexts, our data show that in the present WM paradigm, behavioral impairments arise from disruption of memory maintenance rather than nonspecific behavioral dysregulation.

We have incorporated these results into the figures and revised the text accordingly to explicitly interpret the nature of the impairment as reflecting working memory disruption rather than motor or attentional confounds.

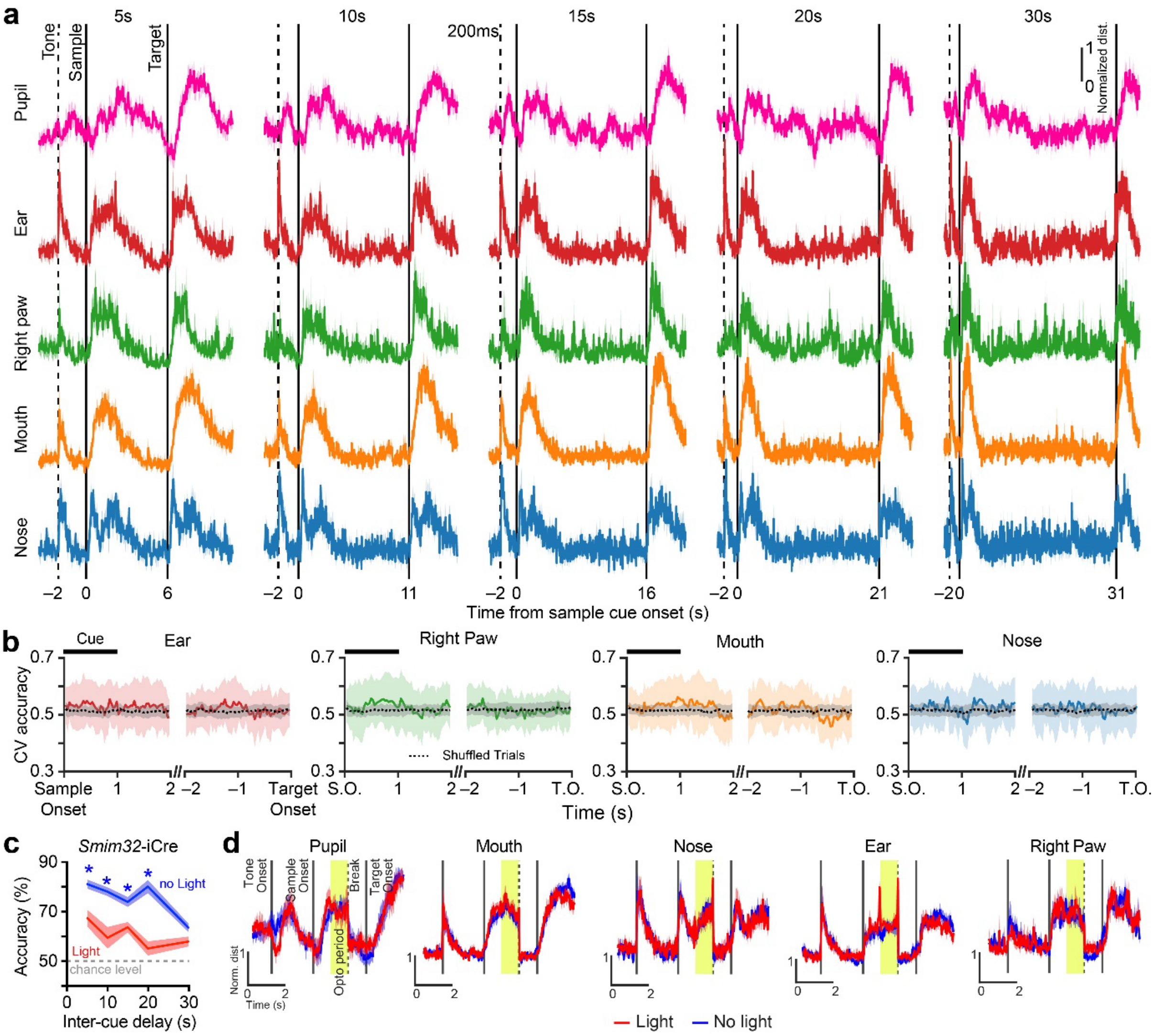

Supplementary Figure 4. Tracking various body movements during WM sessions with optogenetic inhibition of CLA neurons.
a, Normalized pupil area and body-movement traces averaged across mice for all trials with different inter-cue delays (mean ± SEM). Cue onsets (tone, sample, target) are indicated by dashed or solid black lines. For pupil tracking, n = 8 mice (2 mice were excluded due to partially closed eyelids); for other body parts, n = 10 mice. **b,** Classification accuracy for decoding sample cue identity during the OWM task based on movements of different body parts during cue presentation, and early and late delay epochs (All correct trial durations included; shuffled data in black; shaded areas indicate classification S.D.). **c,** WM performance as a function of inter-cue delay in *Smim32*-iCre mice expressing halorhodopsin in the CLA. Light and no-light trials are compared (n = 10; RM ANOVA; post-hoc Fisher's test $P < 0.05$). **d,** Comparison of normalized movement traces (mean ± SEM) across body parts (pupil, mouth, nose, ear, right paw) between light and no-light trials. To pool data across trials with different inter-cue delays, traces were truncated from 1 s after sample offset to 1 s before target onset. The yellow shaded area indicates the optogenetic stimulation window during light trials. See **Supplementary Table 1** for detailed statistics.

4. The authors' measure of decrease in performance during inactivation ("% change in accuracy", line 676) is nonstandard and confusing. If I understand it correctly, if the no light

accuracy was 100% (perfect), and they drop to chance (50%), their measure of drop in performance is -100%, not -50% (which is the standard way of reporting deficits). If the no light accuracy was 70% and drops to 60%, their measure reports this as a -50% change in accuracy instead of -10% (the standard way). I understand part of the rationale for defining % change in accuracy this way (floor effects), but because it is nonstandard it needs to be explained clearly in the main text. For example the authors make a point of highlighting the large performance drop numbers without explicitly explaining their measure (lines 192, 206, 244-248) is different than standard. In lines 244-248 they write "reducing CLA excitability resulted in a significant decline in WM performance, averaging approximately 40-50%. In some cases, performance dropped by as much as 70% for specific delays, with a complete shutdown observed in some individual mice. The impact of these manipulations was notably strong." More, the name for the measure in the text and axis labels should also be changed from "% change in accuracy" (something I would interpret as meaning the standard way) to something like "accuracy change index" to alert readers to the difference.

We thank the reviewer for this helpful and important observation. We agree that our original use of the term "% change in accuracy" was nonstandard and potentially misleading, as it does not correspond to a simple percentage-point decrease in performance. As the reviewer correctly noted, our intent in using a normalized metric was to account for floor effects and to quantify proportional performance loss relative to each animal's baseline accuracy. This approach more accurately captures the impact of CLA inactivation across cohorts with differing baseline performance (**Supplementary fig. 3e** and **Supplementary fig. 5a,b**) and enables meaningful comparisons across inter-cue delays.

To this end, we defined a **Relative Performance Index**, which quantifies, for each subject and delay, performance changes relative to the corresponding control condition. To address the reviewer's concern and improve clarity, we have implemented the following changes throughout the revised manuscript:

1. **Terminology revision.** We have replaced "% change in accuracy" with "Relative Performance Index" in the main text and all figure axis labels. This makes clear that the metric reflects a normalized change rather than a conventional difference in accuracy.
2. **Methodological clarification.** We now provide a detailed description of the calculation and its rationale in the Methods section, explicitly stating how performance during manipulation is normalized to each subject's control accuracy and how this approach mitigates floor effects.
3. **Clarification of interpretation.** We have revised the Results text to clearly indicate that reported values represent proportional changes relative to baseline performance, avoiding language that could be interpreted as standard percentage-point differences.

We appreciate the reviewer's suggestion, which has improved the transparency and interpretability of our behavioral analyses. These revisions ensure that the magnitude and meaning of the reported effects are clearly and accurately conveyed.

Reviewer #2 (Remarks to the Author):

Bhattacharjee and colleagues investigate the claustrum's role in working memory using a classic delayed non-match to sample task. They show that chemogenetic and optogenetic silencing of the claustrum during various task epochs impairs performance, supporting a role for the claustrum in the task. Through calcium imaging of glutamatergic claustrocortical projection neurons, they report that claustrum neurons exhibit cue-specific and delay-period activity, which persists at the population level during long delay periods.

There are several limitations of the study. First, there is a lack of statistical justification for classifying neural responses into three types (Fig. 2). Neurons are classified into one of three types based on an arbitrary activity threshold. Furthermore, from a conceptual perspective, why "Stimulus neurons", many of which also show increased activity in the inter-cue interval (Fig. 2c), are not also considered "Delay neurons" is not clear. Second, analyses do not attempt to account for variables that may modulate claustrum neuron activity such as movements (including licking), motor preparation, engagement and so on, shown in published studies to affect claustrum neuron activity. Third, a central conclusion of the study is that "Delay neurons" exhibit a sequential representation of working memory (Fig. 2c), with activity tiling the delay period once the neurons are sorted by response latency. The Mrsic-Flogel group recently showed that similar patterns of sequential activity are generated in a simple discrimination task as well as a delayed non-match to sample task (doi: 10.1038/s41586-022-05014-3), indicating that these signals may not uniquely reflect working memory (see also Fig. 3b). Averaging across these "Delay neurons" will inherently produce sustained changes in population activity. Although whether the claustrum contributes to working memory is an important question, the analyses as presented do not provide convincing evidence for sustained delay-period activity specific to working memory.

We thank the reviewer for their detailed and thoughtful critique of our manuscript. We appreciate the opportunity to clarify and refine our analyses in response to the concerns raised. Below, we address both the general and specific comments, and we believe that the additional experiments and analyses included in the revised manuscript substantially strengthen the central claims of the study.

First, regarding the classification of neurons into inhibited, stimulus-, and delay-responsive categories: our initial rationale was to provide an intuitive framework for understanding the temporal response profiles of neurons in relation to the task structure. Furthermore, similar classification schemes have been widely used in previous studies employing comparable tasks[4, 5, 6], and we chose to maintain this approach for consistency. However, we acknowledge that this method relies on threshold-based criteria, which can appear arbitrary. To address this, we have now additionally implemented an unsupervised k-means clustering analysis, which allows for a grouping of neuronal responses on their temporal profiles. These analyses, presented in revised **Figure 2f–h** and new **Supplementary Figure 2d–f**, demonstrate the emergence of stable clusters with distinct temporal dynamics. Irrespective of the classification method used, the average population responses showed sustained, though temporally fluctuating, activity until the target cue was presented for all three inter-cue delays. It is also important to clarify that, although we categorized neurons into different classes, all population-level analyses were conducted using the full set of recorded neurons and were not restricted to only Stim- or Delay-selective neurons.

Second, we recognize the importance of controlling for movement-related variables such as licking, and preparatory motor actions that may confound interpretations of delay-

related activity. In the revised manuscript, we present a comprehensive movement analysis based on video-tracked body parts (nose, mouth, paw and ear, **Supplementary Fig. 4a**) and lick rates (new **Fig. 3d-f** and **Supplementary Fig. 2h**). These analyses show that only a small proportion of neurons (<10%) are correlated with movement, and importantly, that most delay neurons do not exhibit movement-related activity. Furthermore, we found no significant differences in motor behavior (e.g., increased licking or premature responses, change in body movements…) between control and DREADD/Optogenetic manipulation trials, supporting the interpretation that the observed delay activity reflects cognitive maintenance rather than sensorimotor output (see new **Fig. 6a,b** and **Supplementary Fig. 4d**).

Regarding the point about sequential activity and its interpretation: we appreciate the reference to the study by the Mrsic-Flogel group (doi: 10.1038/s41586-022-05014-3), which we also cited and which highlights an important caution regarding low-dimensional representations. However, their sequential activity analysis was limited to short delays (3.2 seconds), during which residual sensory responses may still persist in visual cortical areas such as AM following stimulus presentation. In contrast, our task involved delays of up to 30 seconds, well beyond the duration of typical sensory-evoked responses, and was conducted in a brain region <u>not considered part of a sensory pathway</u>. To further distinguish working memory-related activity from short-lived stimulus-driven transients, we performed additional high-dimensional analyses (new **Fig. 4**), including delay-period decoding (see answers to specific comments below). These analyses demonstrate that cue identity is robustly embedded in CLA population activity (see details below).

Taken together, these results strongly suggest that the CLA supports WM not through generalized task engagement or residual sensory activity, but by maintaining cue-specific information across long temporal delays. While we agree that sustained activity alone is not sufficient to demonstrate mnemonic content, the addition of new decoding analyses, movement controls, and clustering-based validation provides a more robust demonstration that the CLA contributes to active memory maintenance. Finally, these findings are consistent with the effects observed in our optogenetic and chemogenetic manipulations.

We hope these revisions address the reviewer's concerns and clarify the distinction between sustained activity for working memory versus sensory-driven or motor-related activity. See also, the responses to the detailed comments below.

More detailed comments are found below.

Figure 1.

As previous studies have shown that some claustrum neurons respond robustly to movement, an analysis of licks over time for sessions corresponding to the recording data (split by different delays) would address some potential behavioral confounds. An analysis of lick rates and responses based on outcome would also be helpful (hit, FA, CR, miss). An analysis of videos of the mice to assess movement (including potentially tongue movements that do not contact the lick port) as well as pupil size as they relate to claustrum activity would strengthen the paper.

We thank the reviewer for this insightful comment and constructive suggestions. In response, we systematically analyzed and presented the full temporal dynamics of licking behavior across all trial types and inter-cue delays, now shown in **Fig. 1d,e** of the revised manuscript. We further analyzed licking behavior by trial outcome (hits, misses, false alarms, and correct

rejections) and included this detailed breakdown in **Supplementary Fig. 1a**. We also visualized the distribution of behavioral outcomes using Sankey diagrams in **Fig. 1f** and report inter-animal variability in **Supplementary Fig. 1b**. To rule out motor confounds and assess arousal and task engagement, we also trained an additional cohorts of mice and tracked multiple body parts (nose, paw, mouth, and ear) along with pupil dynamics, presented in **Supplementary Fig. 4**.

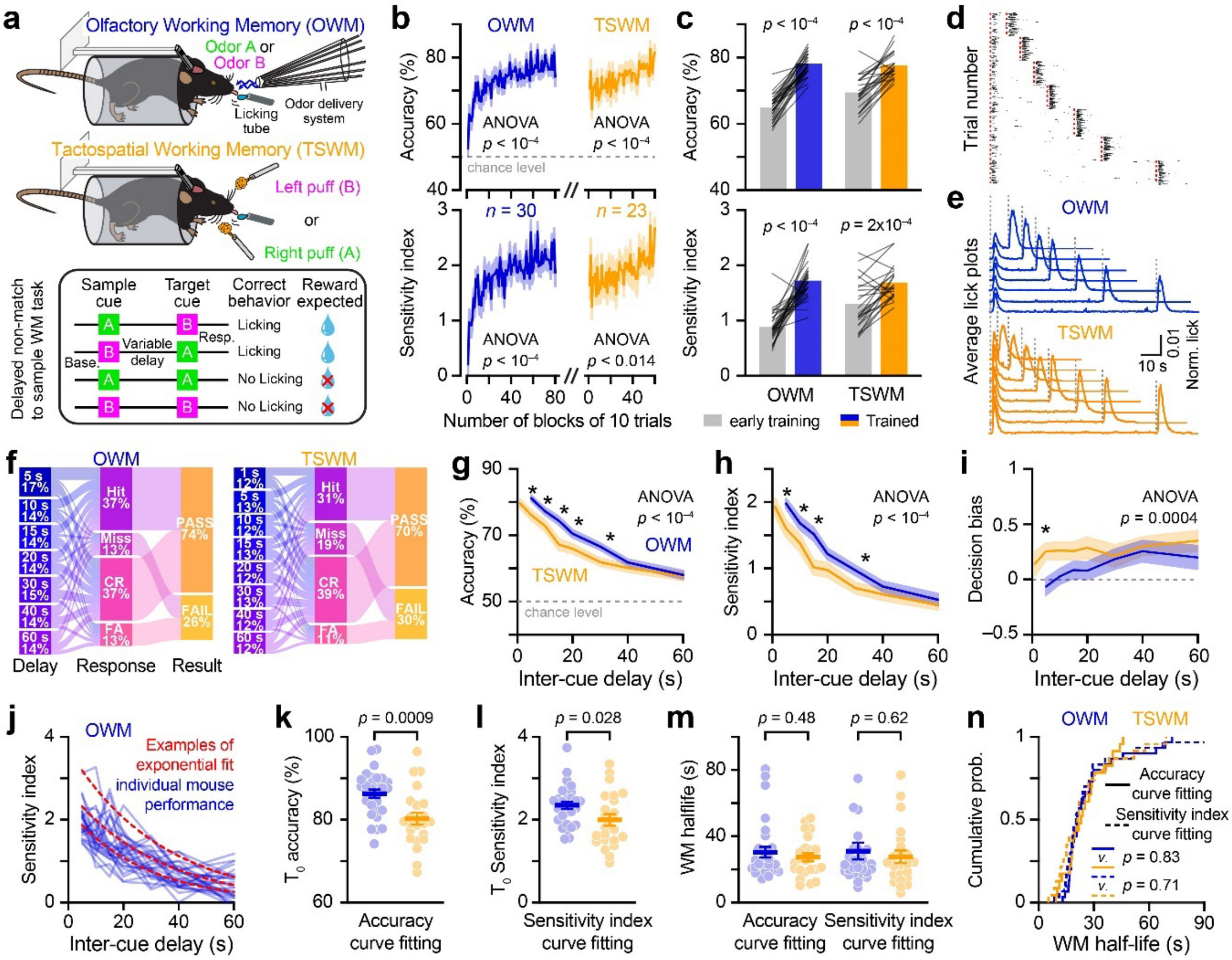

Figure 1. Working memory performance across distinct sensory modalities in mice.
a, Schematic representation of the behavioral paradigm designed to evaluate olfactory and tactospatial WM (OWM and TSWM, respectively) in head-restrained mice. Both sample and target cues were presented for one second. **b**, Learning curves for OWM and TSWM tasks (fixed intercue delay of 5 and 1s, respectively; the number of mice is indicated; one-way repeated measures ANOVA). **c**, Change in performance between the first and the last 100 trials of the training phase shown in **b** for each mouse (black lines; paired t-test) and the mean population performance (colored bars). **d**, Example lick raster from a single mouse performing the OWM task (trials sorted by delay duration). Dotted lines indicate cue onset. **e**, Peri-stimulus time histogram showing normalized lick initiation counts averaged across all mice during OWM and TSWM sessions. **f**, Sankey plots summarizing the distribution of trial outcomes across different delay durations for both OWM and TSWM tasks. CR: correct rejections, FA: false alarms. **g-i**, WM performances plotted as a function of the intercue delay duration (2WRM ANOVA, post-hoc Fisher's test *: $P < 0.05$). **j**, Variability of WM performance across individual animals shown for sensitivity index computed during the OWM task. Examples of exponential fit (used to calculate the amplitude at 0 delay and the half-life) of a few individual performance curves are illustrated (red dashed lines). **k-m**, Comparison of WM performance amplitude at 0 delay and half-life for OWM and TSWM tasks (unpaired t-test). **n**, Cumulative probability distribution of half-lives for OWM and TSWM tasks (Kolmogorov-Smirnov test). Data in **b**, **e**, **g-i**, **k-m** are presented as mean ± SEM. See Supplementary Table 1 for detailed statistics.

Across experiments, mice consistently initiated licking during the sample cue presentation. This behavior, which arose from the pretraining requirement to obtain a small

water reward following cue presentation, was neither discouraged nor punished during later sessions, as it could provide a useful measure of arousal and engagement. While some licking occurred during cue presentation, mice rapidly learned to suppress licking during the inter-cue delay period before responding to the target cue (**Fig. 1d,e**). This behavior was consistent across all delays, indicating sustained task engagement and intact motor control, and demonstrating that mice could withhold licking for up to a minute before responding to the target cue. Notably, lick raster plots revealed distinct temporal licking patterns across trial types, particularly during the response window (**Supplementary Fig. 1a**). Hit trials were characterized by elevated licking specifically during the response period, whereas Correct Rejection trials showed suppressed licking. False Alarms and Misses occurred when this suppression failed, or when licking during the response window did not reach the threshold required for a Hit (see also the response to the comment regarding threshold definition below).

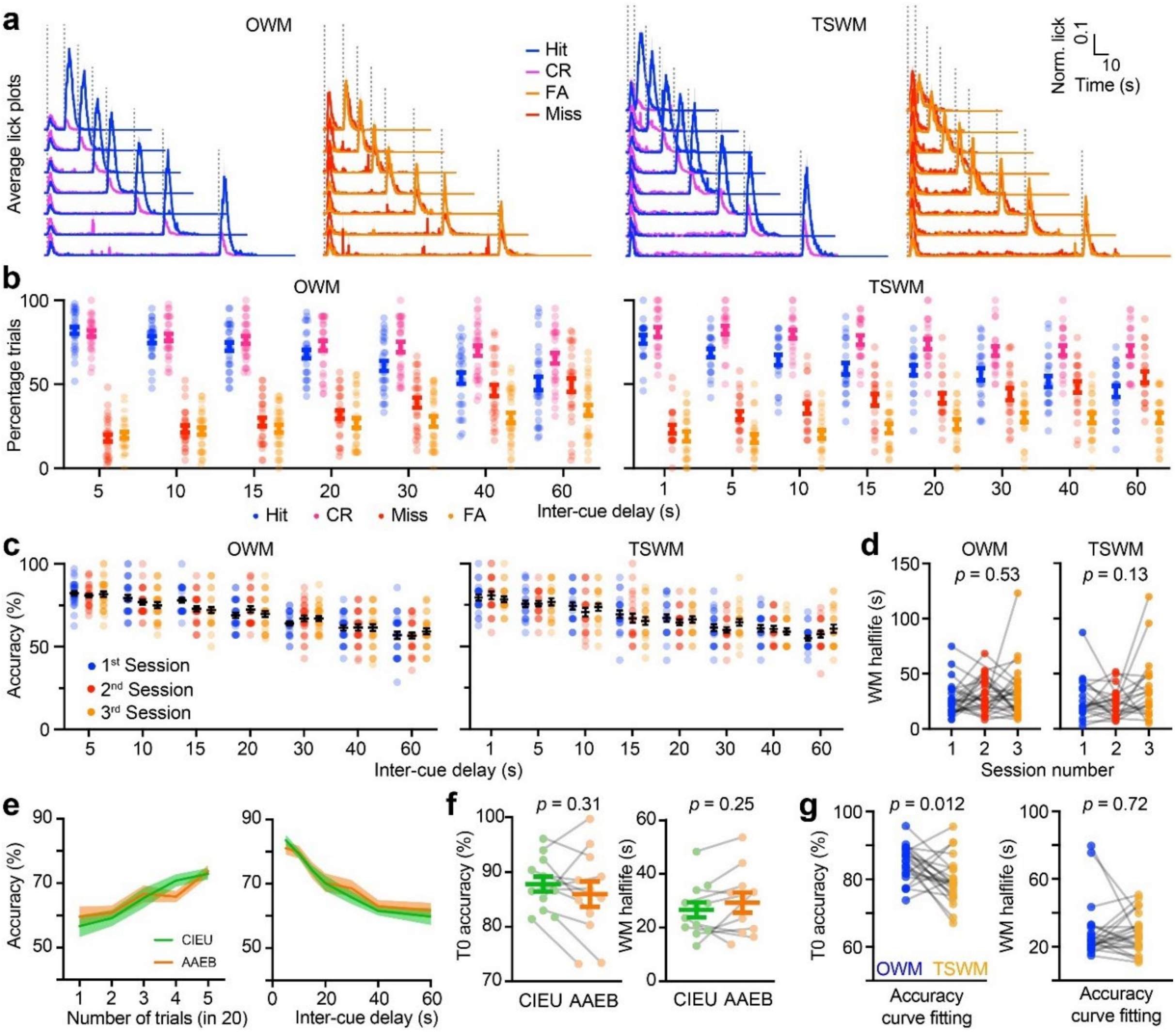

Supplementary Figure 1. Stability of WM task performance across conditions.
a, PSTHs of normalized lick onset events for each behavioral outcome (Hits, Misses, Correct Rejections, and False Alarms) across inter-cue delays in OWM and TSWM tasks. Grey dotted lines mark the onset of sample and target cues. **b,** Proportions of each behavioral response type (hits, misses, correct rejections, and false alarms) across varying inter-cue delays for OWM and TSWM tasks. **c,** Session-wise accuracy comparison across delays in OWM and TSWM tasks. **d,** Stability of WM performance assessed via session-wise half-life values (RM one-way ANOVA: OWM, $P = 0.53$; TSWM, $P = 0.13$). **e,** *Left*: Early learning curves for two odor pairs (CIEU and AAEB) using a fixed 5s inter-cue delay (2WRM ANOVA: $F_{(1,22)} = 0.03$, $P = 0.86$; $n = 12$). *Right*: WM performance across inter-cue delays for the same odor pairs (2WRM ANOVA: $F_{(1,22)} = 0.07$, $P = 0.80$). **f,**

Comparison of WM performance amplitude at 0 delay and half-life for the two odor pairs (Cineol and Eugenol or Amyl acetate and Ethyl butyrate; paired t-test). **g,** Comparison of WM accuracy at 0s delay and the half-life value (parameters derived from exponential curve fitting) for OWM and TSWM tasks (paired *t*-test). Data are presented as mean ± SEM. See **Supplementary Table 1** for detailed statistics.

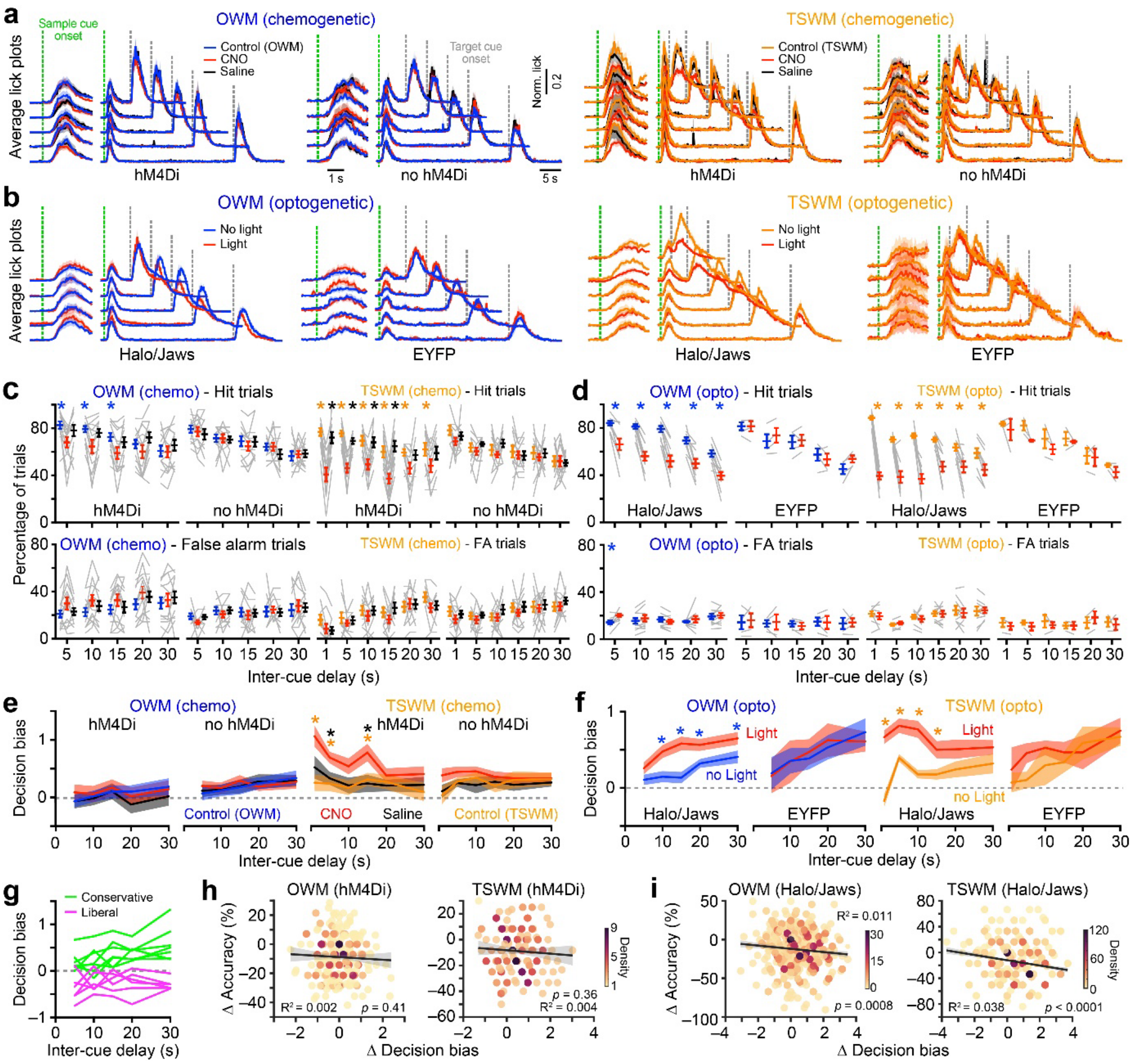

Figure 6. CLA manipulations influence behavioral responses and decision bias.
a-b, PSTH of normalized lick onset events across conditions. Licking after sample cue (*left*) and during full trial (*right*) are shown separately. Green and grey dotted lines mark sample and target onset, respectively. **c,** Percentage of Hit and FA trials during control, CNO, and saline sessions for OWM and TSWM tasks (RM ANOVA; post-hoc Fisher's test comparisons *: P < 0.05, significant comparisons for Control vs. CNO and Saline vs. CNO are color-coded in blue/orange and black, respectively). **d,** Percentage of Hit and FA trials during light and no-light trials for both WM tasks (RM ANOVA; post-hoc Fisher's test *P < 0.05). **e,** Decision bias vs. inter-cue delay for mice expressing hM4Di (OWM: n = 13, TSWM: n = 11) and controls (n = 12 for both). Sessions included control (no injection), CNO, and saline in order (RM ANOVA; post-hoc Fisher's test *P < 0.05; color coding as in **c**). **f,** Decision bias *vs.* delay duration for mice expressing Halo/Jaws or EYFP. Light vs. no-light trials compared within groups (Halo/Jaws: OWM n = 10, TSWM n = 9; EYFP: OWM n = 4, TSWM n = 3; RM ANOVA; post-hoc Fisher's test *P < 0.05). **g,** Individual variability in decision bias (from e) as a function of delay: hM4Di-expressing mice in control OWM sessions showed divergent strategies: conservative (green) vs. liberal (magenta) licking. **h,** Accuracy difference (CNO – control) *vs.* change in decision bias across delay periods (color indicates point density). **i,** Accuracy difference (light – no-light) vs. decision bias change across delays during optogenetic

stimulation epochs (**Fig. 5e**); color shows point density. Data shown as mean ± SEM. See **Supplementary Table 1** for full statistics.

Furthermore, behavioral deficits during CLA inhibition could not be attributed to nonspecific licking or increased licking activity during the delay period. (**Fig. 6a,b**). We found a selective and reproducible reduction in Hit rates (**Fig. 6c,d**), whereas False Alarm rates showed greater inter-individual variability: some animals exhibited increased FA rates, while others showed a reduction. This variability in response strategy was quantified using the classical signal-detection theory metric, *decision bias*. A positive bias reflects more conservative behavior, indicating that mice tend to respond only when more confident in their decision. Although individual strategies varied across animals (**Fig. 6g**) and could fluctuate over time, we observed a general tendency toward more conservative responding as task difficulty increased, for instance, with longer delay durations or when tactile cues were used, even during control sessions or control trials in opto- and chemogenetic experiments (**Fig. 1i** and **Fig. 6e,f**). Overall, mice shifted toward more conservative responding during CLA inhibition, although this effect was not consistent across all conditions (e.g., chemogenetic inhibition during OWM tasks; **Fig. 6e**). Importantly, changes in decision bias were not strongly correlated with performance decline between manipulated and control conditions (**Fig. 6h,i**), suggesting that memory impairment was not driven by altered response criterion or motor suppression.

The tracking of various body parts was performed as suggested (see also the correlation with neural responses below). Across trials, gross body movements were minimal and primarily associated with salient periods, such as the tone signaling trial onset and the sample and target cues (**Supplementary Fig. 4a**). Notably, even during long delays, mice remained behaviorally engaged without excessive movements that could inadvertently trigger lick sensors or cause spurious errors. Interestingly, movement information from body parts that can be consciously controlled by the animals could not be used to predict which sample cue was presented either during cue presentation or during early and late delay periods across all correct trials (**Supplementary Fig. 4b**). This suggests that mice did not rely on specific body postures associated with particular cues to solve the task without engaging working memory. Pupil size fluctuated throughout the task and delay periods, largely in parallel with movements of other body parts. Finally, optogenetic activation of halorhodopsin did not significantly alter any body movements compared to interleaved control trials (**Supplementary Fig. 4d**).

Collectively, these results argue against nonspecific motor effects or distraction as explanations for the performance deficits, and instead indicate that CLA inactivation disrupts the internal maintenance of task-relevant information essential for WM–guided decisions. The manuscript text and figures have been updated accordingly to incorporate these results.

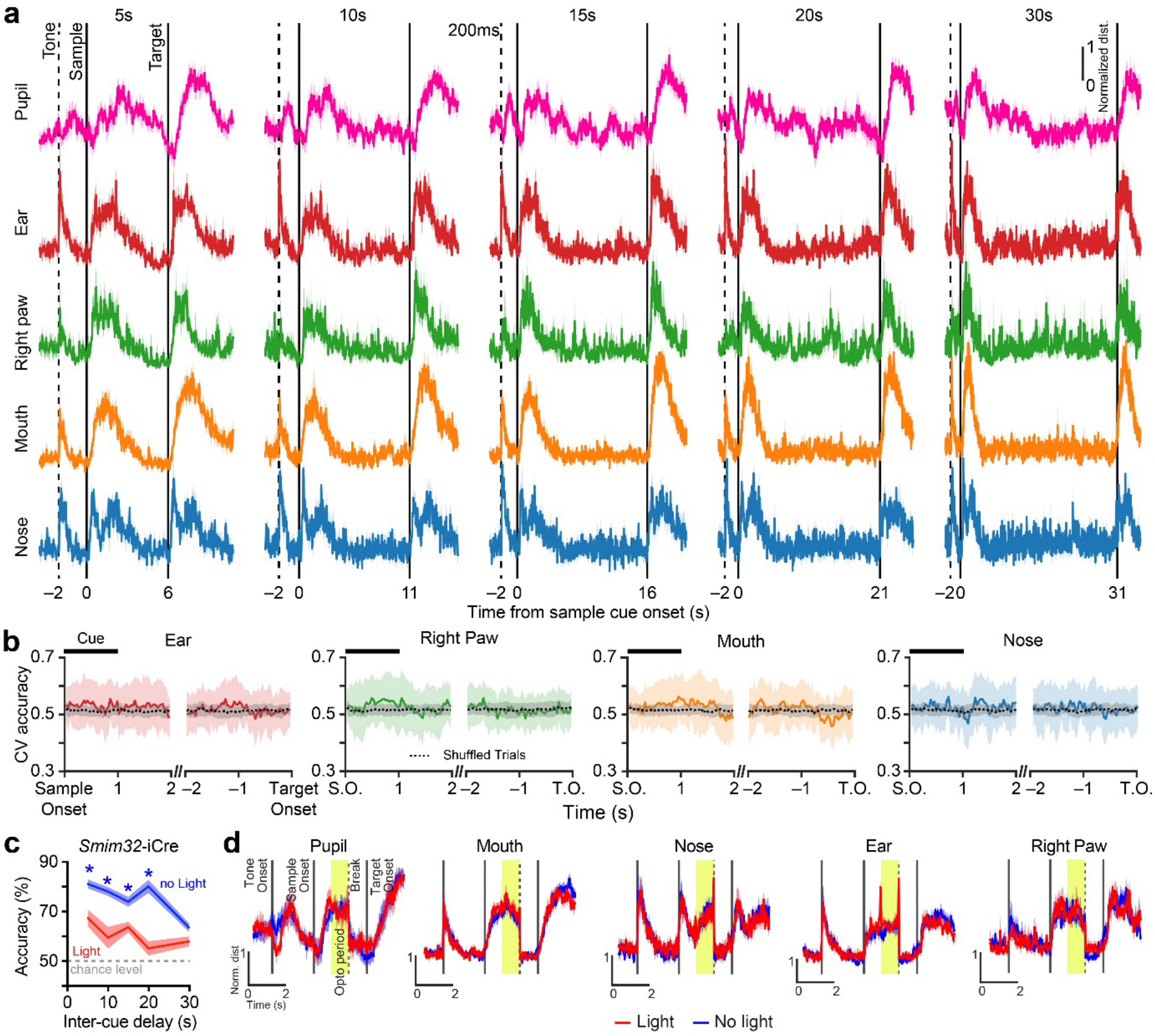

Supplementary Figure 4. Tracking various body movements during WM sessions with optogenetic inhibition of CLA neurons.

a, Normalized pupil area and body-movement traces averaged across mice for all trials with different inter-cue delays (mean ± SEM). Cue onsets (tone, sample, target) are indicated by dashed or solid black lines. For pupil tracking, *n* = 8 mice (2 mice were excluded due to partially closed eyelids); for other body parts, *n* = 10 mice. **b,** Classification accuracy for decoding sample cue identity during the OWM task based on movements of different body parts during cue presentation, and early and late delay epochs (All correct trial durations included; shuffled data in black; shaded areas indicate classification S.D.). **c,** WM performance as a function of inter-cue delay in *Smim32*-iCre mice expressing halorhodopsin in the CLA. Light and no-light trials are compared (*n* = 10; RM ANOVA; post-hoc Fisher's test $P < 0.05$). **d,** Comparison of normalized movement traces (mean ± SEM) across body parts (pupil, mouth, nose, ear, right paw) between light and no-light trials. To pool data across trials with different inter-cue delays, traces were truncated from 1 s after sample offset to 1 s before target onset. The yellow shaded area indicates the optogenetic stimulation window during light trials. See **Supplementary Table 1** for detailed statistics.

How exactly are 'hit' trials identified based on the change in lick rate?

During training, mice learned to associate non-match trials with the need to lick within a designated response window to obtain a reward. However, as mentioned above, we observed that mice frequently licked during the sample cue presentation and occasionally engaged in anticipatory licking during the delay period (**Fig. 1d,e** and **Supplementary Fig. 1a**). To account for these behaviors and ensure accurate trial classification, a non-match trial was scored as a 'Hit' only if the number of licks during the response window exceeded the number

of licks recorded during the pre-response period (defined as the interval from cue onset to the start of the response window). Trials that did not meet this criterion were classified as 'Misses'. Because all licks occurring during the sample cue and throughout the entire delay period (up to 60 seconds for the longest delay) were integrated, this approach likely discouraged mice from engaging in spontaneous or excessive licking, as such behavior would be difficult to compensate for during the much shorter response window. This scoring rule is now explicitly described in the Methods section to enhance clarity and reproducibility.

Why do mice perform at greater than 60% accuracy on average the first day of training on the full task structure (Fig. 1b and c) rather than chance accuracy expected in untrained mice? And similarly, why does performance plateau at 60% accuracy at long delays instead of dropping to chance? These questions also relate to the justification for the fits (line 499-506) as performance and d' are not at 50% or 0 even in the first behavioral session.

We thank the reviewer for bringing up this point. In the initial manuscript, we plotted training accuracy as averages over 100 trials, which may have inadvertently suggested that mice performed above 60% accuracy from the very first session. To clarify this, we have now replotted the learning curves using 10-trial bins (see new **Fig. 1b**). These updated plots show that performance starts at chance levels (or a sensitivity index of ~0) in the earliest trials and progressively increases, surpassing 60% accuracy within the first 100 trials. This rapid improvement likely reflects the effects of the structured pre-training phase that preceded the main training. During pre-training, mice were taught to reliably respond to non-match trials by licking within a defined response window. As a result, when they transitioned to the full task, which includes both match and non-match trials, they already had an established behavioral strategy for non-match detection. Initially, mice also tend to lick during match trials, but they quickly learned to suppress this behavior in the absence of a reward, demonstrating successful task acquisition. We have clarified these points in the Methods section.

In our experimental design, match and non-match trials were presented in equal proportion. Thus, if a mouse licked indiscriminately (or refrained from licking altogether) on all trials, it would achieve a theoretical accuracy of 50% and a sensitivity index of zero. The same values would be expected in the case of complete WM loss, assuming no bias in the task design. Behavioral data showed a clear decrease in performance with increasing inter-cue delay (**Fig. 1g,h**). Consistent with prior WM literature, the group-averaged performance across mice was well fit by a single exponential decay function (see **Figure Rebuttal 1a** for an example fit of the OWM data from **Fig. 1g**; $R^2 = 0.998$, ANOVA $p < 10^{-4}$). Since no parameters were fixed, the fit yielded three free parameters, including the WM lifetime (t1) and the asymptotic performance at infinite delay (y_0). Importantly, y_0 converged to the theoretical chance level (51.7 ± 1.8%), suggesting no overt bias in the task design. Notably, behavioral performance at the longest delay (~58%) closely matched the empirical fit. Furthermore, when plotting theoretical exponential decay functions with the asymptotic performance (y_0) fixed at 50% and using the same amplitude (A_1) as the empirical fit, along with two plausible half-lives (20 and 30 seconds, approximating the median and mean from individual fits), the predicted performance at 60 seconds closely aligned with the observed data (*left panel* of **Figure Rebuttal 1b**). The same conclusion held when assuming perfect initial performance (100% at time zero, *right panel* of **Figure Rebuttal 1b**). In summary, while subtle experimental biases cannot be completely excluded, the close match between behavior and the theoretical exponential decay function indicates that memory degradation follows expected WM decay dynamics.

Finally, based on these observations, we fixed the y_0 parameter at 50% accuracy (or d′ = 0 for sensitivity index fits) when modeling individual behavioral performance with an exponential function. This reflects the theoretical baseline expected at the chance level. We chose to constrain this parameter because doing so significantly improved the quality and stability of the fits, particularly given the noisier nature of individual datasets, especially when analyzing performance within a single session (~100 trials in total).

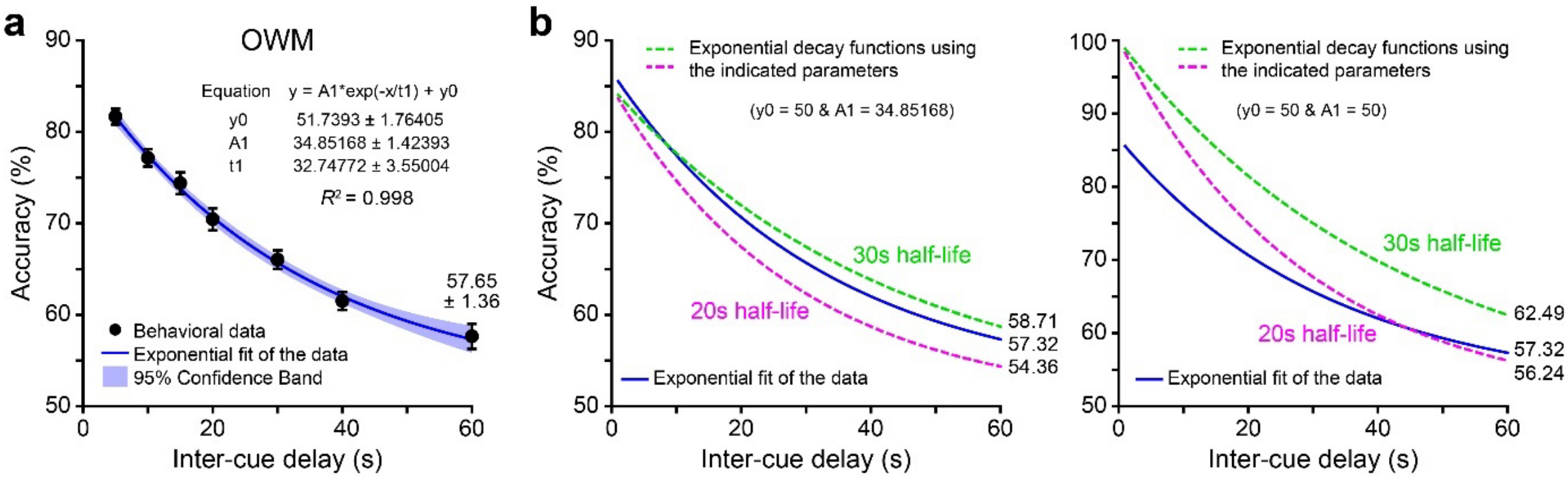

Figure Rebuttal 1. Behavioral performance across mice follows a single exponential decay function. **a,** Group-averaged performance across mice for the OWM data shown in **Fig. 1g** was well fit by a single exponential decay function (ANOVA, $p < 10^{-4}$). The fit returned three free parameters: the amplitude constant (A_1), the working memory lifetime (t_1), and the asymptotic performance at infinite delay (y_0). Actual behavioral performance at the 60s delay is indicated numerically. **b,** *Left panel:* Comparison between the empirical fit (blue line) and theoretical exponential decay functions with the asymptotic performance (y_0) fixed at 50% and using the same amplitude (A_1) as the empirical fit and two plausible half-lives (20 s and 30 s, magenta and green dashed lines, respectively, corresponding approximately to the median and mean values from individual fits). Expected performance at the 60 s delay is reported numerically. *Right panel:* Comparison between the empirical fit (blue line) and theoretical decay functions with the asymptotic performance (y_0) fixed at 50% and assuming perfect initial performance (100% at time zero), using the same two plausible half-lives (20 s and 30 s, magenta and green dashed lines, respectively). Expected performance at the 60 s delay is also indicated.

Related to lines 474-476 - What criteria are used to determine that mice "learned to perform the task" within 200 to 300 trials (Fig. 1b)? What criteria are used to determine that mice were "proficiently trained" to then expose them to the DNMS task with variable delay periods within a single session? The same questions relate to the tactile version of the task (lines 494-496). What criteria were used to demonstrate "mastery" (line 214)?

We apologize for the lack of clarity regarding this point. In our experiments, mice were considered to have "learned the task" once they consistently performed with an accuracy exceeding 70%. This threshold was selected because it reflects robust task engagement and reliable discrimination between match and non-match trials. Specifically, performance was evaluated in blocks of 10 consecutive trials, and mice were considered trained once they maintained ≥70% accuracy across several such blocks. As shown in the learning curves (**Fig. 1b**), although early performance could fluctuate, mice typically reached this benchmark within 200–300 trials. Once consistent performance above the threshold was achieved, animals were transitioned to the delayed non-match to sample (DNMS) task, which incorporated variable delay durations within a single session.

For the tactile version of the task, the same performance criterion was applied. Mice were deemed to have achieved "mastery" once they reached and maintained ≥70% accuracy in 10-trial blocks during the initial training phase with short (1-second) delays. Only then were they introduced to sessions with longer, variable delays.

We have now explicitly added this training criterion to the revised main text and Methods section to ensure clarity and transparency in our behavioral assessment strategy.

How did performance vary across the three sessions for each mouse? Did performance vary initially when the odorants were switched in n = 12 mice? Were mice that performed "better" than average at the OWM task also "better" at the TSWM task (Fig. 1e)?

We thank the reviewer for these important questions. In our experiments, we observed some session-to-session variability in performance within individual mice across the three sessions and delay durations. This variability can be due to the limited number of trials per session (approximately 100), which can introduce noise into performance estimates at each delay. Additionally, behavioral fluctuations may reflect varying levels of attention or engagement across sessions. To mitigate these effects, we typically report performance averaged across at least three sessions, providing a more reliable estimate of behavioral accuracy. Despite the individual variability, population-level performance across delays remained relatively stable across sessions for both the OWM and TSWM tasks (**Supplementary Fig. 1c**). To further assess WM dynamics at the individual level, we estimated the WM half-life for each session. These analyses revealed that WM decay rates were, on average, consistent across sessions, indicating that individual performance instability did not substantially confound the population-level results (**Supplementary Fig. 1d**).

Regarding the odor switch in 12 mice, we briefly investigated whether task performance would generalize to a new odor pair or remain odor-specific. After training the mice with a novel odor pair (Amyl Acetate/Ethyl Butyrate), we observed a marked drop in performance during the initial sessions. However, with continued training, performance gradually improved, following a trajectory similar to that observed with the original odor pair (see early portion of the learning curve in **Supplementary Fig. 1e**, left panel). Interestingly, WM performance across delay durations was comparable between the two odor sets, as indicated by the stability of the fitted free parameters (**Supplementary Fig. 1f**, right panel). This suggests that the underlying WM process remains stable when stimuli are easily discriminable. However, we do not claim that WM performance is entirely invariant, as it could be influenced by the perceptual similarity between cues. A more detailed investigation of this question lies beyond the scope of the current study and would require a dedicated follow-up.

Finally, in response to the question regarding cross-modality performance, we examined whether mice that performed well in the OWM task also exhibited higher performance in the TSWM task. We found no significant correlation between the two measures: some mice that performed well in the olfactory task showed reduced WM capacity in the tactile task, and vice versa (**Supplementary Fig. 1g**). It remains unclear whether this reflects biological day-to-day behavioral variability (as discussed above), the limited sample size, or a genuine dissociation of WM performance across sensory modalities. A more comprehensive investigation will be required in future studies to resolve this question.

Figure 2.

The paper rests in part on identifying three response types but the statistical justifications for this classification are unclear. Why choose an arbitrary threshold of one to classify neurons rather than principled approaches for identifying clusters (lines 539-545)? It is also unclear what proportion of neurons are not significantly modulated during the task. As neurons that do

not cross the threshold are considered inhibited, it seems that unmodulated neurons and statistically significantly inhibited neurons are grouped together.

We thank the reviewer for this valuable and thoughtful comment. We agree that our previous description of the classification method lacked statistical clarity, and we have revised and expanded the Methods section to provide a more transparent and rigorous explanation. As detailed in the revised manuscript, we classified neurons into three functional categories (Stimulus neurons, Delay neurons, and Inhibited neurons) based on their temporal response profiles during the WM tasks. This classification relied on the latency of the first inferred spike obtained from calcium deconvolution, computed using a fast non-negative deconvolution algorithm [7]. Significant spike events were identified using a threshold of +1.5 z-scores. Neurons showing a significant increase in activity during the sample cue were classified as "Stimulus neurons" (note that stimulus neurons could still have significant changes during the delay), whereas those showing a significant increase during the delay period were classified as "Delay neurons." Similar classification approaches have been used in prior studies employing related behavioral paradigms. Neurons exhibiting significant decreases in activity (≤ -1 z-score) during any part of the delay were classified as "Inhibited neurons". We note that virtually all recorded neurons showed significant activity changes at some point during the delay across the different delay durations. Because non-responsive neurons cannot be reliably identified from miniscope recordings and because we applied conservative criteria for identifying true calcium events, the reported percentages reflect only the subset of claustral neurons for which detectable activity changes could be measured, rather than the entire underlying neuronal population.

To assess whether observed activity exceeded chance levels, we employed a permutation-based statistical test. Each neuron's activity was circularly shifted by a random interval (± 500 ms) 1,000 times to generate a null distribution of trial-averaged activity. A neuron was classified as significantly active if its trial-averaged peak activity exceeded the 95th percentile of this shuffled distribution. This approach ensured that classification was based on statistically meaningful deviations from chance, rather than arbitrary thresholds. Over 90% of neurons classified as Stimulus or Delay neurons passed this criterion, confirming their robust task-evoked responses.

In addition, to address the reviewer's concern regarding the use of arbitrary thresholds and to provide a more data-driven approach, we performed an unsupervised clustering analysis. Specifically, we applied *k*-means clustering to trial-averaged responses from all neurons across correct trials and across all delay durations (responses were thus truncated at 5 s into the delay), testing *k* values from 2 to 20. The optimal number of clusters ($k = 3$) was determined using the elbow method, based on the second derivative of the sum of squared errors (SSE). Clusters were visualized using t-distributed stochastic neighbor embedding (t-SNE). Subpopulation calcium activity traces were plotted and neuronal activity heatmaps were generated by sorting neurons within each cluster according to response latency (**Fig. 2f–h** and **Supplementary Fig. 2d–f**). Notably, this unsupervised approach largely reproduced the principal response motifs identified using our initial latency-based classification, although it tended to amplify apparent fluctuations in calcium activity during the delay period. Overall, the similarity between the two approaches supports the robustness of our response categorization.

Importantly, while these classifications help interpret population response dynamics, our primary WM analyses (e.g., state-space trajectories) were performed on the full neuronal population, independent of response class. Thus, our main conclusions regarding the claustrum's role in WM do not depend on this classification scheme. Furthermore, we

replicated these findings in an independent dataset recorded from the CLA using a different Cre line targeting claustral neurons (**Supplementary Fig. 2**).

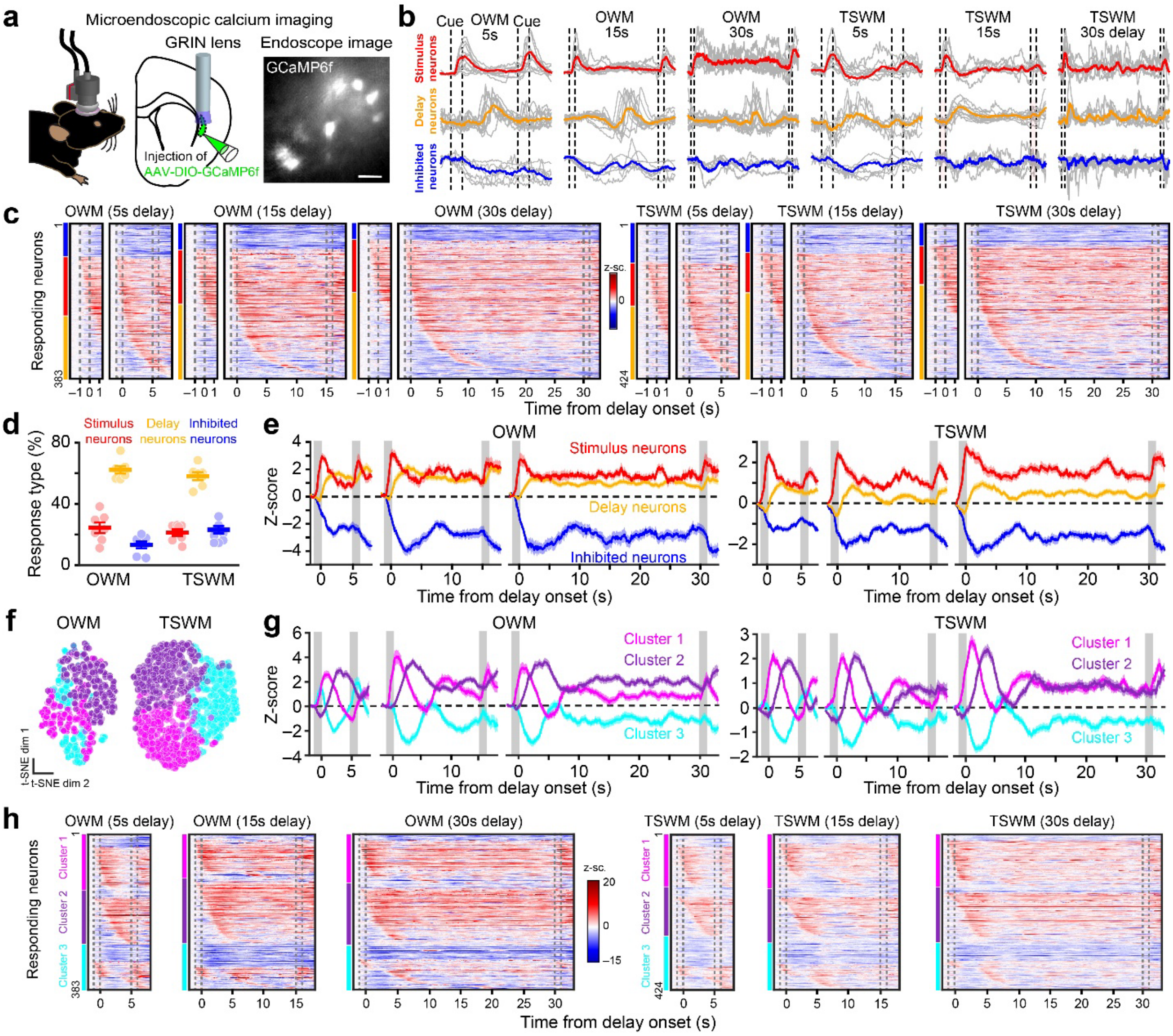

Figure 2. Persistence of claustrum neuron responses after sensory cue presentation.
a, Schema of the calcium imaging procedure. Image of CLA neurons expressing the genetically encoded calcium indicator GCaMP6f captured through the GRIN lens of the endoscope camera (scale bar: 50 µm). **b,** Examples of individual neuron responses during OWM and TSWM tasks. Grey and colored traces represent individual and averaged correct trials for different response types, respectively. Dashed black lines mark cue application periods. **c,** Pseudo-colored heat maps presenting trial-averaged responses from correct trials for all stimulus, delay, and inhibited neurons (distribution indicated by the color lines on the left, same code as in **b**). Neurons were pooled from 7 mice and sorted by response type and latency (z-score min: -15, max: 20 and 15 for OSW and TSWM maps, respectively). For each delay duration, an expanded zoom including the first second of the delay is shown. Dashed grey lines indicate cue applications. **d,** Quantification of the different response types recorded in different mice (each data point represents the mean value computed across different delays for each animal; data presented as mean ± SEM). **e,** Average population calcium activity (data presented as mean ± SD). Grey bars represent cue applications. **f**, t-SNE projection of neurons clustered using k-means clustering (all delay durations used). **g,** Average population calcium activity of neurons assigned to each cluster (mean ± SD). Grey bars indicate the timing of cue presentations. **h**, Pseudo-colored heatmaps showing trial-averaged responses from correct trials for the three clusters identified using k-means clustering (Cluster identity is indicated by the color bars on the left, matching the color scheme used in **f**). Neurons were pooled from 7 mice and sorted by response latency within each cluster. Dashed grey lines indicate cue applications.

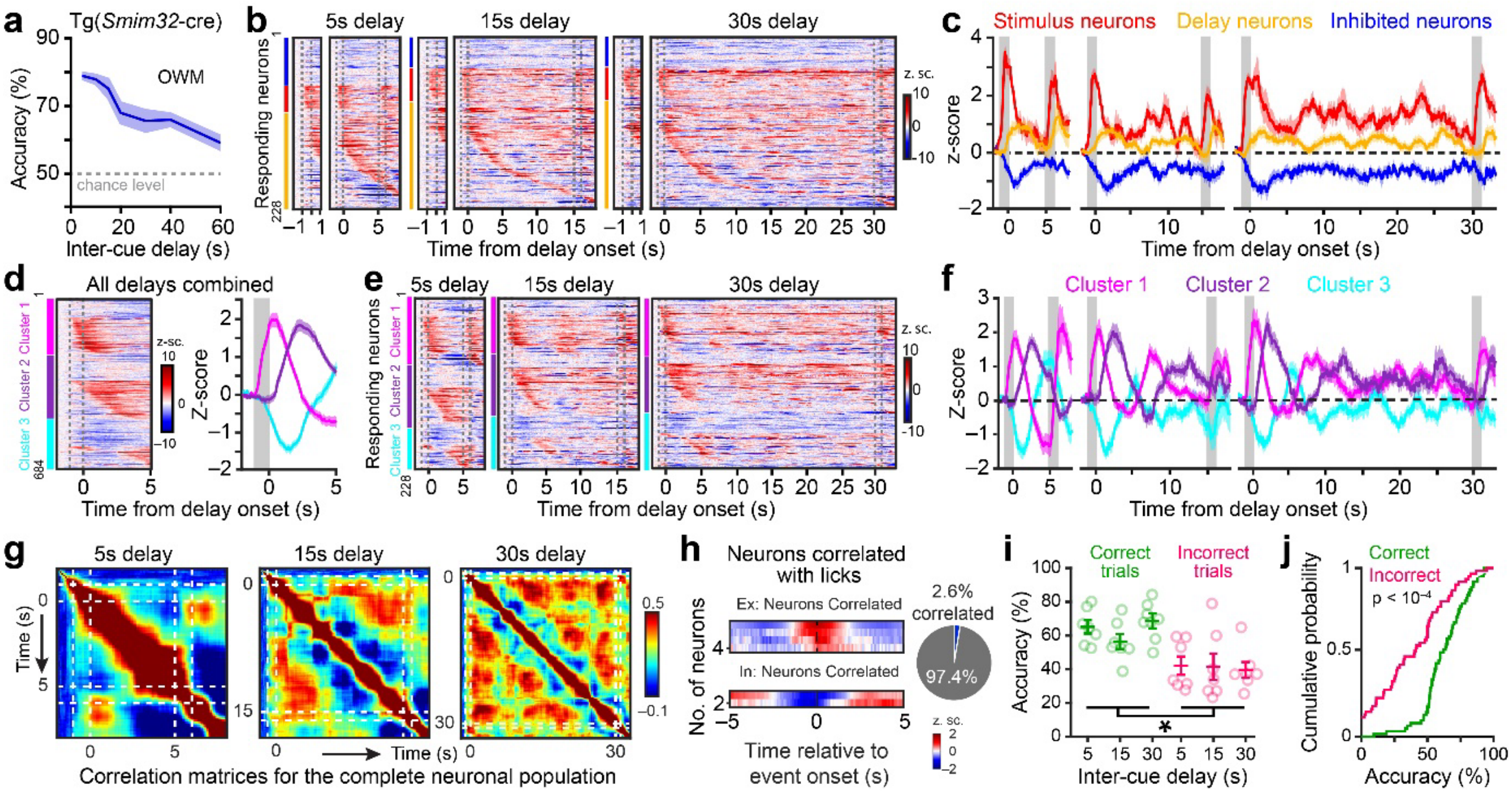

Supplementary Figure 2. Sustained responses of *Smim32*⁺ CLA neurons following cue presentation. **a,** WM performance as a function of inter-cue delay duration during imaging sessions (n = 7 mice). **b,** Pseudo-colored heatmaps showing trial-averaged responses from correct trials for all stimulus-, delay-, and inhibition-responsive neurons (distribution shown by color lines on the left). Neurons pooled from 7 Tg(*Smim32*-cre) mice and sorted by response type and latency (z-score range: –10 to +10). For each delay duration, an expanded zoom including the first second of the delay is shown. Dashed grey lines indicate cue applications. **c,** Average population calcium activity (mean ± SD). Grey bars mark cue applications. **d,** Pseudo-colored heatmaps (*Left panel*) and average population calcium activity (*right panel*, mean ± SD) for neurons in three clusters identified using k-means (all delay durations used). Neurons (n = 228) pooled from 7 mice and sorted by response latency within each cluster. Responses across all delay durations are shown. Dashed grey lines and the gray box indicate cue timing. **e-f,** Same as in **d**, but neural data are plotted separately for each delay duration. **g,** Correlation matrices showing pairwise correlations of population activity vectors over time (bin size: 100 ms). Dashed white lines mark cue onset. **h,** Heatmaps of average responses from neurons significantly correlated with lick onset, grouped by excitatory or inhibitory responses. *Right*: Pie chart showing proportion of lick-correlated neurons (blue) relative to the total recorded population. **i,** Classification accuracy for decoding sample identity during late delay periods in correct and incorrect trials (n = 7 mice, data presented as mean ± SEM; correct vs. incorrect trials: RM ANOVA $F_{(1,6)} = 21.33$, $P = 0.0036$). **j,** Cumulative probability distributions of sample cue classification accuracy for correct and incorrect trials (n = 63 animal/delay/session triplets). See **supplementary Table 1** for detailed statistics.

Controls demonstrating that claustrum neurons are indeed responding to the sensory stimuli rather than to other observable variables are needed. For example, claustrum neurons are modulated during motor responses like licking (doi: 10.1016/j.neuron.2021.11.013; doi: 10.1101/2021.03.23.436687). These prior studies also showed that anterior claustrum neurons respond rarely to sensory stimuli, prompting the question of why the results differ in these experiments. Do animals lick during the first cue presentations? Linear models, for example, assessing the correlation of claustrum activity with multiple variables and their interactions would strengthen the paper.

We thank the reviewer for this valuable and thoughtful comment. First, we examined whether CLA neurons are genuinely selective for sensory stimuli (see new **Fig. 4a-d** in the answer to the next comment). We found that approximately 50% of the stimulus neurons were selective for either Sample A or B while only 2.78–4.76% of neurons were activated by both sample cues, indicating low overlap and strong cue specificity independently of the sensory dimension used. This cue-selectivity analysis should not be confused with the proportion of Stimulus

neurons out of the entire population we observed in **Fig. 2**, as those analyses were done by averaging all correct trials irrespective of the type of sample presented.

Second, the reviewer correctly notes that previous studies have reported modulation of CLA neurons during motor-related events, including licking and limited responsiveness of anterior CLA to sensory stimuli. To address the former concern (see also the description of the licking behavior in the answer to a previous comment), we performed multiple analyses to disentangle sensory-driven activity from motor-related modulation in our dataset (see also next paragraph). We performed a temporal correlation analysis between calcium activity and lick traces. Only a small fraction of CLA neurons showed significant correlations with licking, approximately 8% in *Vglut2*-cre mice and 3% in Tg(*Smim32*-cre) mice (**Fig. 3d** and **Supplementary Fig. 2i**, respectively). Although this difference may reflect normal experimental variability, it could also indicate subtle differences in the neuronal populations recorded. Indeed, while all claustral $Vglut2^+$ neurons are $Smim32^+$, only ~50% of $Smim32^+$ neurons express Vglut2 (See Tuberosa et al. 2024, [8]). Thus, although these data suggest that $Smim32^+$ CLA neurons may be even less correlated with licking, this does not change any of the conclusions presented above (see **Supplementary Fig. 2**).

Third, as suggested by the reviewer, we extended our analysis to examine whether CLA neurons respond to body movements beyond licking. This analysis was performed exclusively in *Smim32-cre* mice, as video recordings were not available for the *Vglut2-cre* cohort. We found that approximately 12% of neurons showed significant correlations with at least one tracked body movement (**Fig. 3f**), while no neurons were correlated with ear movements or fluctuations in pupil size. Thus, although some claustral activity is related to body movements, this accounts for only a minority of neuronal responses during the working memory (WM) task. Notably, the observed neural activity preceded movement onset, suggesting that these signals are unlikely to reflect movement execution per se and may instead relate to motor planning or preparatory processes. Importantly, body movements could not be used to decode sample identity (**Supplementary fig. 4b**). Therefore, movement-related calcium activity is unlikely to account for or confound our analyses of odor identity coding.

Fourth, differences in response tuning relative to previous studies may arise from the fact that those works examined functionally or anatomically restricted claustro-insular subpopulations (e.g. S1-projecting neurons in Chevée et al., 2022 [9]). The claustro-insular region contains a highly heterogeneous mixture of neuronal subtypes, with multiple populations coexisting within small spatial scales [10]. This anatomical and functional complexity may contribute to why some authors did not observe robust sensory-evoked responses. However, it is also possible that cue-selective responses emerge through task training or are expressed only under behavioral states requiring specific cognitive demands. For example, Ollerenshaw and colleagues (10.1101/2021.03.23.436687) reported that *Gnb4*$^+$ CLA neurons do not respond to passively presented visual or auditory stimuli but show strong modulation during an active Go/No-Go sensory detection task. Our optogenetic inhibition experiments are consistent with this view. In a simple discrimination task without a working memory component, optogenetic inhibition of CLA neurons did not impair performance (**Fig. 5j**), indicating that CLA activity is not required for immediate sensory discrimination. In contrast, inhibition during WM tasks produced marked deficits in the same mice (**Fig. 5k**), suggesting that the CLA becomes essential when sensory representations must be actively maintained across a delay. Consistent with hierarchical models of cortical processing[11], these results support the idea that CLA neurons do not encode low-level sensory features, but instead represent abstracted, task-relevant information (e.g. "this was Sample A") necessary for WM–guided decisions.

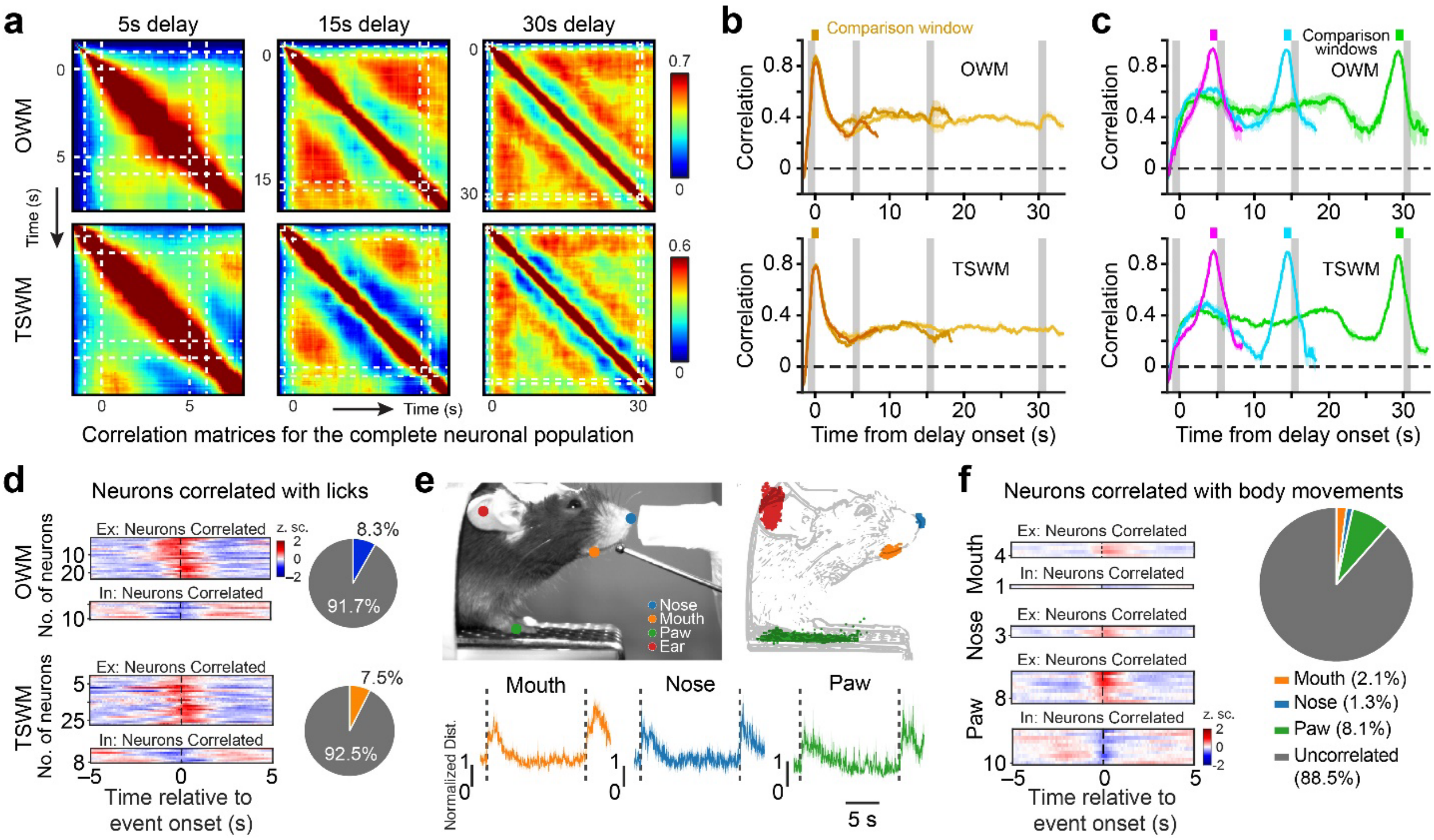

Figure 3. CLA population dynamics and its correlation with body movements.
a, Correlation matrices showing all possible correlations computed between pairs of population activity vectors at different time points (time bin = 100 ms). Dashed white lines represent cue presentation. **b-c**, Time course of ensemble correlations for different trial structures (average of the four conditions: sample A–target B, sample A–target A, sample B–target A, sample B–target B; mean ± SD), plotted for various delay durations in the two WM tasks. Comparison windows (1 s) are color-coded above the graphs; grey bars denote cue presentation. **d,** Pseudo-colored heatmaps of neurons significantly correlated with lick onset, grouped by excitation or inhibition. The pie chart indicates the fraction of lick-correlated neurons relative to the total recorded population for OWM and TSWM tasks (blue and orange, respectively). **e,** Video snapshot showing body-part tracking during CLA imaging in a WM task. Right panel: representative coordinates for mouth, nose, and right paw. Below: normalized movement traces (mean ± SEM) averaged across mice for all trials with a 15 s inter-cue delay. **f,** Pseudo-colored heatmaps of neurons correlated with body movements, aligned to movement onset, grouped by excitation or inhibition. The pie chart shows the proportion of neurons correlated with movements of each body part (mouth, nose, right paw) relative to the total population in OWM tasks. No neuronal activity was correlated with ear movement.

Taken together, our data indicate that sensory information can be maintained within CLA population activity for delays up to 30 s, even though some motor-related modulation is also present. Future work will be needed to determine the conditions under which sensory response properties change and to identify the factors that shape these dynamics across tasks and behavioral states.

As noted above, the Mrsic-Flogel group showed that similar patterns of sequential activity are generated in a simple discrimination task as in a delayed non-match-to-sample task (doi: 10.1038/s41586-022-05014-3), indicating these signals may not uniquely reflect working memory. It is not clear from the analyses included that the claustrum has sustained delay-period activity related to working memory.

Regarding the point about sequential activity and its interpretation, we appreciate the reference to the study by the Mrsic-Flogel group (doi: 10.1038/s41586-022-05014-3), which we also

cited and which highlights an important caution regarding low-dimensional representations. However, their sequential activity analysis was limited to short delays (3.2 seconds), during which residual sensory responses may persist in visual cortical areas such as AM following stimulus presentation. In contrast, our task involved delays of up to 30 seconds, well beyond the duration of typical sensory-evoked transients, and we recorded a brain region not considered part of a sensory pathway. To further distinguish long-lived working memory signals from short-lived stimulus-driven transients, we performed additional analyses (new **Fig. 4**). We assessed how reliably cue identity was maintained by the CLA population on individual trials. To this end, we applied a decoding approach to predict cue identity across animals, delays, and tasks. Specifically, we implemented a bootstrapping procedure to randomly sample correct trials and trained a classifier to determine whether CLA population activity could discriminate between sample cues during the WM task. Classifier performance was evaluated during two epochs: the early delay (ED; defined as the first second after the sample period) and the late delay (LD; the final second preceding target presentation). Classifier performance during both ED and LD periods was comparable and consistently exceeded chance levels (i.e., relative to shuffled data), indicating that cue identity was retained throughout the delay (**Fig. 4f,g**). Furthermore, decoding accuracy declined at longer delays (**Fig. 4h**). Notably, although fewer incorrect trials were available, classifiers trained on correct trials performed poorly when applied to incorrect trials, with prediction accuracy markedly reduced across delays and tasks (**Fig. 4g,h and Supplementary fig. 2i,j**). Together, these results indicate that cue identity is robustly decodable at the single-trial level and that this information is broadly represented across the CLA population during the delay period. These findings suggest that the CLA not only encodes cue-specific information during stimulus presentation but also maintains this information across the delay, with neural decoding performance paralleling behavior.

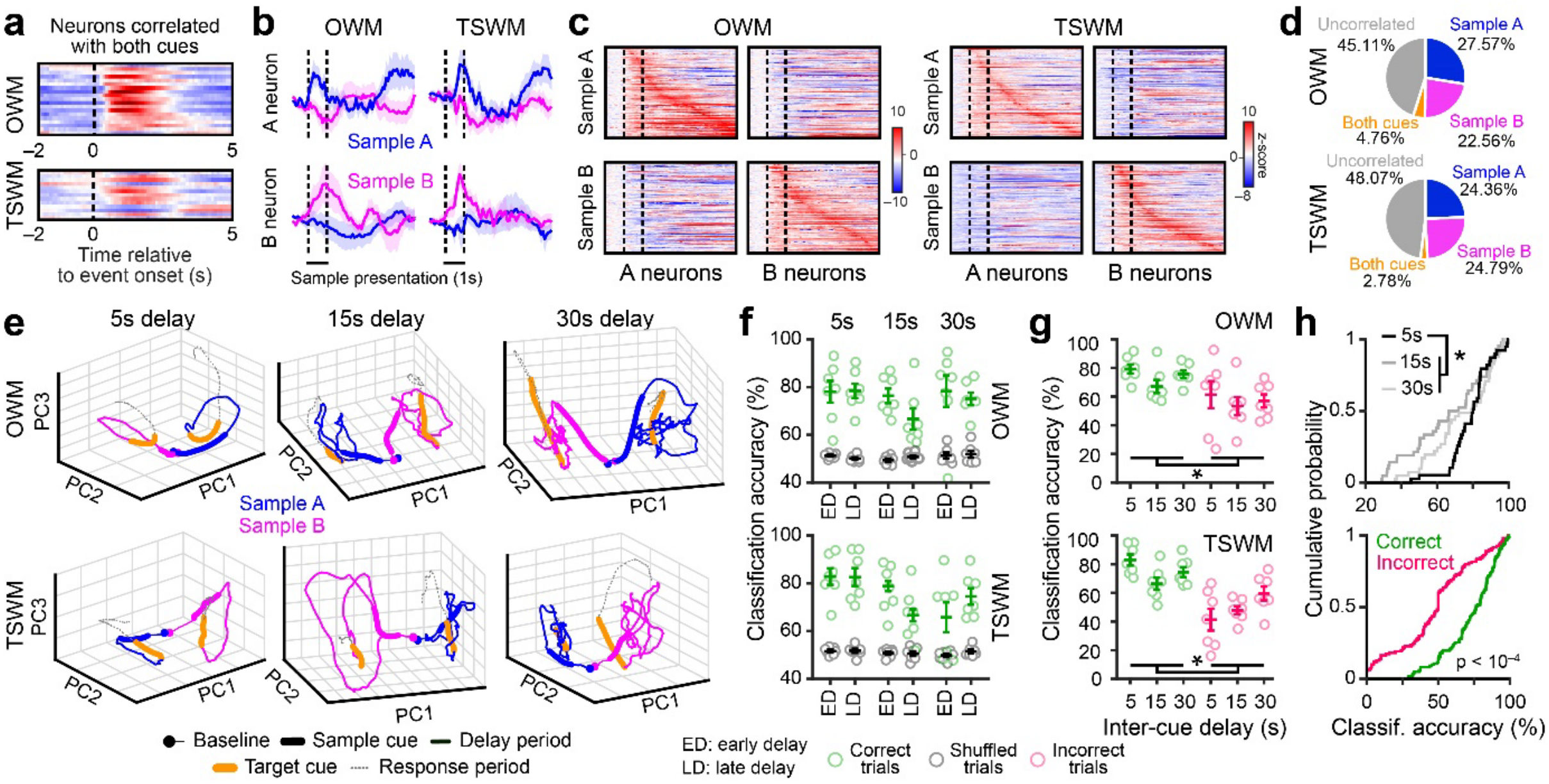

Figure 4. Claustrum ensembles maintain cue information during working memory.
a, Pseudo-colored heatmaps of neurons significantly correlated with both Sample A and B cues during OWM and TSWM tasks. **b,** Averaged calcium traces from four cue-selective stimulus neurons (mean ± SD). Each neuron responded to one cue but often showed complex delay responses for the non-preferred cue. **c,** Heatmaps of trial-averaged responses from all cue-selective stimulus neurons, sorted by peak latency for the preferred cue. Neuron order was preserved for non-preferred cue (n = 165, 150, 187, 191 neurons for samples A & B in OWM and TSWM tasks, respectively). **d,** Pie charts showing proportions of neurons correlated with both sample cues or only one, relative to the total recorded population in OWM and TSWM tasks. **e,** CLA population activity

trajectories for the averaged correct trials with Sample A or B, plotted in 3D PCA space. **f,** Classification accuracy for decoding sample identity during the early delay (ED; 1s after sample) and late delay (LD; 1s before target) periods in correct and shuffled trials for both tasks (*n* = 7 mice, 3-way RM ANOVA: shuffling effect $F_{(0.77,9.25)}$ = 125.1 $P = 3.10^{-6}$, ED vs. LD periods: $F_{(1,12)}$ = 1.36 P = 0.27 and $F_{(0.70,8.36)}$ = 130.3 $P = 5.10^{-6}$, ED vs. LD periods: $F_{(1,12)}$ = 0.004 P = 0.95, for OWM and TSWM, respectively). **g,** Classification accuracy for decoding sample identity during late delay periods in correct and incorrect trials for both tasks (*n* = 7 mice, correct vs. incorrect trials: RM ANOVA $F_{(1,6)}$ = 15.48, P = 0.0077 and $F_{(1,6)}$ = 27.71, P = 0.0019 for OWM and TSWM tasks, respectively). **h,** Cumulative probability distributions of sample cue classification accuracy. *Top graph*: correct trials for different delays (*n* = 42 animal/session/task triplets, Kolmogorov-Smirnov test $P = 3.10^{-4}$, P = 0.009 and P = 0.29 for 5s vs. 15s. 5s vs. 30s and 15s vs. 30s, respectively). *Bottom graph*: comparison between correct and incorrect trials (*n* = 126 & 124 animal/delay/session/task quadruplets for correct & incorrect trials, respectively). Data are presented as mean ± SEM. See **supplementary Table 1** for detailed statistics.

The experiments included do not demonstrate that "Delay neurons" resemble hippocampal or entorhinal "time cells." Whether, for example, they encode absolute or relative time is not characterized (doi: 10.1016/j.neuron.2011.07.012).

We thank the reviewer for raising this important point. We have modified the description in the text. Our intention is not to equate the delay neurons observed in the CLA with the "time cells" classically described in the hippocampus, entorhinal cortex, or prefrontal cortex. Time cells are defined by their consistent and temporally precise firing at specific moments within a task interval, often thought to encode either absolute or relative time within that structure. The Delay neurons that we have observed in CLA show variable activation depending on the kind of sample stimuli presented. Specifically, we observe that stimulus-selective neurons can exhibit dynamic activity patterns, being active during the stimulus period for one cue, suppressed when the alternate cue is presented, or even persistently active during the delay (**Fig. 4c**). This suggests that Stimulus and Delay neurons do not represent entirely separate subpopulations but instead reflect flexible functional states within the same neural ensemble. Thus, these Delay neurons were not individually tuned to fixed time bins in a way that would allow direct comparison to time cells. Rather, we interpret their activation as reflecting internal mnemonic representations that support cue retention during WM maintenance.

High ensemble correlation after sample onset may reflect stereotyped behaviors during the delay period rather than working memory per se, particularly as any correlations must be interpreted in light of how these neurons were initially classified. Further, whether the correlations reflect any aspect of the behavior - for example, does ensemble activity differ across correct and incorrect trials of the same delay and does it predict performance - is not clearly addressed.

We thank the reviewer for this valuable comment. In the revised manuscript, we have included comprehensive analyses of licking behavior and tracked whole-body movements throughout the trial period to directly address the concern that high ensemble correlations after sample onset might reflect stereotyped behavior rather than WM-related processes (see our answers to previous comments above). Across these analyses, we found no evidence of repetitive or stereotyped movements that could account for the persistent neural activity observed during the delay phase. Mice suppressed licking after the initial cue and exhibited minimal movement during the delay, supporting the interpretation that sustained claustral activity reflects internal cognitive processes rather than overt behavioral patterns. Consistent with this, movement information from tracked body parts could not predict sample identity during cue presentation or during early and late delay periods across all correct trials (**Supplementary Fig. 4b**).

Importantly, our ensemble correlation analysis was performed across the full population of recorded neurons rather than being restricted to predefined functional classes

(e.g., Delay neurons). This inclusive approach enabled us to capture global network dynamics associated with cue encoding and maintenance. The high ensemble correlation immediately following sample onset reflects the emergence of a coordinated population response, whereas the subsequent fluctuations during the delay indicate evolving population states, consistent with dynamic WM representations rather than passive sensory or motor processes. In line with this, our high-dimensional decoding analyses demonstrate that CLA population activity reliably predicts behavioral outcomes.

Notably, classifiers trained on correct trials could decode sample identity from single-trial activity during the delay, and their performance was markedly reduced when applied to incorrect trials. This indicates that claustral population representations are degraded or less consistent on error trials, linking population dynamics to behavioral performance rather than reflecting movement-related activity. We note, however, that the relatively low number of incorrect trials per session, particularly at delays where performance remained largely above chance, limited further analyses of error-related activity. Specifically, this constraint precluded reliable single-neuron comparisons between correct and incorrect trials, as well as the training of classifiers exclusively on incorrect trials. Similar limitations are common in studies combining similar behavioral tasks with single-photon calcium imaging. Future work leveraging approaches that enable stable longitudinal tracking of identified neurons across multiple sessions, such as two-photon imaging combined with improved registration methods, will allow pooling of incorrect trials across days. This will increase statistical power and enable a more detailed characterization of how population activity differs between correct and error trials.

Does sustained activity at the end of the delay period, which best decodes stimulus identity, predict correct behavioral responses? Conversely, does decayed activity correspond to incorrect trials, suggesting a failure in maintaining working memory-related information?

We thank the reviewer for this question. This issue has been addressed in the response above.

Figure 3.

What is the relationship between the population of neurons classified as “cue” selective in Fig. 1 and the neurons classified here using stimulus-specificity measured by Wilcoxon rank sum test? Do all three “types” of neurons in Fig. 1 contribute to the cue-selective stimulus neurons in Fig. 3? And in what proportion?

We thank the reviewer for this question. The “cue-selective” neurons analyzed in **Fig. 2** were identified by averaging each neuron’s activity across all correct trials, irrespective of stimulus identity (Sample A or Sample B). This approach was intended to provide an unbiased overview of the temporal diversity of CLA responses during the delayed WM task, without presupposing stimulus-specific tuning. In contrast, the stimulus-specific analysis in the new **Fig. 4** was explicitly designed to determine whether individual neurons preferred Sample A or Sample B. For this analysis, trials were separated by sample identity, and responses were compared using a non-parametric Wilcoxon rank-sum test. Neurons showing significantly stronger responses to one cue were classified as cue-selective.

When examining these cue-selective neurons, we found that the three functional response categories identified in **Fig. 2** do not correspond to strictly segregated subpopulations. Instead, they reflect flexible functional states expressed by the same underlying ensemble. As

noted above, stimulus-selective neurons can display dynamic patterns, responding during the sample period for one cue, being suppressed for the alternate cue, or remaining active into the delay (**Fig. 4c**).

To directly address the reviewer's question, we have now added a quantitative summary of cue-selective neuron distribution in the revised manuscript. We also identified a subset of neurons that responded similarly to both cues; these could contribute to general task-related processes, such as encoding task structure or trial timing, rather than specific sensory identity per se.

The analysis based on a Wilcoxon rank-sum test assumes differences in activity are due to stimulus specificity, thus it is not clear that the neurons are "tuned" to the stimuli. If the neurons are stimulus-responsive, one might predict that the sensory response would be similar in correct and incorrect trials. Are the results for each cell the same if incorrect trials are analyzed separately? Why were regression-based methods that could account for multiple variables – such as lick rate – not applied?

We thank the reviewer for this thoughtful and important comment. We fully agree that the Wilcoxon rank-sum test detects differences in activity between two conditions (in our case, Sample A versus Sample B trials) but does not by itself demonstrate mechanistic "tuning". To further address this concern, we performed a temporal correlation analysis to identify neurons that responded to both types of stimuli irrespective of behavioral outcome. This analysis revealed a small subset of neurons that were stimulus-responsive to both cues (**Fig. 4a**). However, because stimulus presentation is typically accompanied by short bouts of licking and modest increases in body movement, these neurons might contribute to general task-related processes (e.g., encoding task structure or timing) rather than selective sensory coding.

Regarding the reviewer's second point, we agree that regression-based approaches, such as generalized linear models (GLMs) or hierarchical/mixed-effects models, provide a powerful framework for disentangling the contributions of multiple variables, including sensory identity, movement, licking, trial outcome, and delay duration. However, two constraints limited the application of such methods in the present study. First, regression models require a large number of repeated observations per condition to yield stable parameter estimates. In our dataset, particularly for incorrect trials, the number of samples per neuron was low due to high behavioral accuracy and the inability to reliably track the same neurons across multiple days. Second, our primary objective was to identify cue-selective neurons under well-controlled correct-trial conditions. For this purpose, the Wilcoxon rank-sum test provided a simple, conservative, and statistically transparent approach, without the risk of overfitting that more complex models might entail, given our data limitations. Nevertheless, we fully acknowledge the value of multivariate regression-based frameworks and agree that these approaches will be highly informative in future work, particularly once longitudinal tracking of individual neurons across sessions becomes feasible.

What is the distribution of neurons with different response types across mice? It is not clear what Fig. 3e is demonstrating as the template-matching classifier may assign classifications based on the highest correlation, even when correlations are very weak. The analysis also lacks statistical validation (e.g., permutation tests, cross-validation).

We thank the reviewer for this question. The distribution of neurons exhibiting different response types across animals has already been quantified and is presented in **Fig. 2d**. This

panel shows the relative proportions of stimulus-selective, delay-active, and inhibited neurons for each individual mouse.

Regarding the template-matching classifier used in the former Fig. 3e, we agree that such methods can assign class labels based on relative similarity even when absolute correlation values are low. We have therefore removed this analysis and replaced it with a linear classifier approach incorporating bootstrapping and label shuffling to rigorously validate decoding performance (**Fig. 4f-h** and **Supplementary fig. 2i,j**).

Figure 4.

Although the authors do show that stimulus discrimination itself is not disrupted by silencing, alternative interpretations should be considered as silencing of the claustrum may have effects (i.e., on engagement, motivation, pain as shown in recent studies) that may contribute to or account for degraded behavioral performance without affect working memory encoding specifically.

We thank the reviewer for raising this important point. We agree that manipulations of the CLA could, in principle, influence general internal states such as engagement, motivation, or arousal, potentially impairing task performance independently of WM processes. This is a valid concern, particularly in light of recent studies suggesting a role for the CLA in regulating broader behavioral states and/or salience detection.

Given the widespread reciprocal connectivity of the CLA with cortical and subcortical regions, optogenetic or chemogenetic perturbations may affect distributed networks beyond WM-specific functions. For example, prior work has shown that the claustro-cingulate pathway can exert complex, and in some cases opposing, effects on pain modulation[1, 2, 3]. To determine whether optogenetic inhibition of CLA neurons during WM tasks induced nociceptive-, stress-, or arousal-related responses, we monitored pupil size and body movements in *Smim32*-cre mice expressing eNpHR3.0 in CLA neurons (n = 5 males and 5 females), with light delivered for one second following sample cue presentation (**Supplementary fig. 4**). While CLA inhibition impaired WM performance (**Supplementary fig. 4c**), it did not produce detectable differences in pupil dynamics or gross body movements between light and no-light trials. (**Supplementary fig. 4d**). In all conditions, movements were minimal and largely restricted to salient task epochs such as trial onset tone and cue presentations (**Supplementary fig. 4a**), and animals remained behaviorally engaged even during long delays without excessive movement that could spuriously influence responses. Moreover, movement features from voluntarily controlled body parts failed to predict cue identity during sample cue presentation or delay periods in no-light trials (**Supplementary fig. 4b**), indicating that animals did not rely on posture-based strategies in place of WM. Taken together, these results suggest that the observed performance impairments cannot be attributed to pain, stress, altered arousal, or gross motor dysfunction.

We next examined whether the reduction in WM performance could be attributed to impaired motor execution, particularly the licking responses used to report choices. Lick patterns showed no systematic differences during sample cue presentation and delays across chemogenetic or optogenetic manipulations (**Fig. 6a,b**), indicating intact motor output and task engagement[12]. Importantly, licking traces revealed no increase in premature or sustained licking during the delay period, even at long delays, arguing against heightened impulsivity or impaired response withholding under CLA inactivation. Instead, CLA inhibition produced a selective and reproducible reduction in hit rates (**Fig. 6c,d**), while false alarm rates varied

across individuals, suggesting alterations in decision strategy rather than disengagement. Consistent with this, mice showed heterogeneous baseline response biases, spanning conservative to liberal strategies, and tended to adopt more conservative response criterion as task difficulty increased (e.g. longer delays or when using tactile cues), even in control conditions (**Fig. 1i** and **Fig. 6e-g**). Although CLA inhibition sometimes increased this conservative bias, the effect was not consistent across conditions. Moreover, changes in decision bias did not reliably correlate with performance declines between manipulated and control trials (**Fig. 6h,i**), suggesting that WM impairments are unlikely to arise from shifts in response criterion or motor suppression.

Together, these analyses make it unlikely that sensory deficits or nonspecific factors (including pain, altered arousal, impulsivity, impaired inhibitory control, disengagement, motor abnormalities, or decision bias) account for the performance impairments observed following chemo- and optogenetic manipulations. Instead, they support a direct role for the CLA in maintaining task-relevant information across delay periods. Consistent with this interpretation, discrimination performance remained intact in control tasks lacking a delay, indicating that the CLA is not required for immediate sensory processing.

In summary, our data demonstrate that the performance impairments observed during CLA inhibition reflect a specific disruption of WM maintenance rather than nonspecific deficits in task engagement, motivation, or motor execution.

Which aspects of behavior (hits, FA, CR, misses) are most influenced by CLA silencing? Affecting different aspects of behavior (i.e., hit rate, FA rate) could similarly alter performance measures plotted here.

Thank you for raising this point. This issue has already been addressed in detail in our responses above.

Discussion.

Alternative interpretations of the data should be more strongly considered, and some claims (i.e., lines 227-228) should be more qualified. For example, whether CLA ensembles represent "working memory engrams" (line 239) or whether it functions as "RAM" (i.e., lines 277-287) has not been shown by the analyses presented.

We thank the reviewer for emphasizing the importance of considering alternative interpretations and appropriately qualifying our mechanistic conclusions. We therefore carefully evaluated whether CLA manipulation affects general physiological processes such as pain perception, arousal, or motor function. Specifically, we assessed pain- and stress-related responses by monitoring body movement and pupil-linked arousal in *Smim32*-iCre mice during optogenetic inhibition. These analyses revealed no significant differences between light-on and light-off trials (**Supplementary Fig. 4**), indicating that the observed behavioral impairments are unlikely to arise from nonspecific discomfort or distress.

To further exclude motor-related confounds, we analyzed licking behavior across experimental conditions (**Fig. 6a,b**). Lick execution remained intact, with mice exhibiting normal lick rates and temporal patterns during both control and manipulation sessions. Although CLA inhibition consistently reduced Hit rates, False Alarm rates varied across individuals, consistent with shifts in decision strategy rather than motor suppression (**Fig. 6c–g**). Importantly, changes in task accuracy did not correlate with changes in decision bias (**Fig.**

6h,i), reinforcing the conclusion that CLA disruption selectively impacts memory-related processes rather than general behavioral engagement or motivation. We further demonstrated that WM information is robustly represented in CLA population activity and remains decodable throughout the delay period.

We also acknowledge that the CLA is unlikely to serve as the brain's sole site of memory storage. Instead, consistent with its widespread connectivity, sustained activity, and content-specific coding, our data support a role for the CLA as a component of distributed WM circuits that contributes to maintaining task-relevant information. In line with the reviewer's comment, we have revised the manuscript to remove language implying a dedicated storage site or "RAM"-like function (or as the locus of "working memory engrams"), and instead emphasize a more integrative and network-based interpretation of CLA function.

Methods.

Lines 310-316 –How were any differences among genotypes assessed?

We thank the reviewer for raising this important point, which we have now clarified in the revised text. In this study, we examined multiple cohorts of mice that differed in genetic background and/or viral manipulations. Specifically, we employed three Cre driver lines: *Vglut2*-Cre, Tg(*Smim32*-Cre)61^{Irod}, and *Smim32*-iCre (knock-in line). The genetic characterization of the Tg(*Smim32*-Cre)61^{Irod} BAC transgenic line and the *Smim32*-iCre knock-in line has been comprehensively described in a previous study[28]. That work demonstrated that *Smim32*-Cre selectively labels a broad population of excitatory CLA projection neurons with minimal off-target expression. The primary difference between the BAC transgenic and knock-in lines lies in the fraction of the *Smim32*-expressing CLA neuron population that undergoes recombination, with a reduced amount observed in the BAC transgenic line. Importantly, the same study reported substantial overlap between *Smim32*-expressing neurons and *Vglut2*$^{+}$ glutamatergic neurons within the CLA, indicating that these Cre lines together provide robust and complementary access to the excitatory CLA neuron population.

In the revised manuscript, we imaged both *Smim32*-Cre and *Vglut2*-Cre mouse lines under identical task conditions and using the same imaging setup to directly assess the similarity of neural response properties across genotypes. We compared their activity profiles and observed highly comparable patterns of stimulus selectivity, temporal dynamics, and task-related modulation during the WM task (**Figs. 2–3** and **Supplementary Fig. 2**). These similarities were consistent across all major analyses, including cue-selective responses and persistent activity during the delay period.

In the revised manuscript, we also report WM performance across multiple mouse cohorts that differed in genetic background and/or viral manipulations. **Supplementary Fig. 5a** presents overall OWM performance under control conditions for each group. We did not observe any systematic differences between genotypes. Instead, modest variations across cohorts likely reflect intrinsic inter-individual variability and, more plausibly, differences in training duration (e.g. Tg(*Smim32*-Cre)61^{Irod} with optogenetic inhibition). Notably, the only statistically significant difference occurred between the cohort that underwent prolonged training and those with substantially shorter training periods. We further report, across multiple figures in the study, results from four independent cohorts combining different Cre driver lines with distinct optogenetic or chemogenetic inhibitory actuators. A consistent and robust finding is that reducing CLA excitability, regardless of the Cre line or inhibitory tool used, produced a

marked impairment in WM performance (**Supplementary Fig. 5b**). We note that the magnitude of the behavioral effect varied somewhat across genotypes and manipulations, likely reflecting differences in the fraction of CLA neurons undergoing recombination as well as variation in the efficacy of excitability suppression across actuators (**Supplementary Fig. 5c**). These differences between the strongest line–manipulation pairs remained apparent, although not statistically significant, after normalizing performance changes to individual control sessions to account for inter-cohort variability (**Supplementary Fig. 5d**).

Taken together, these findings support the conclusion that Smim32-Cre and Vglut2-Cre mice target overlapping claustral neuron populations with functionally similar contributions to WM within our behavioral paradigm.

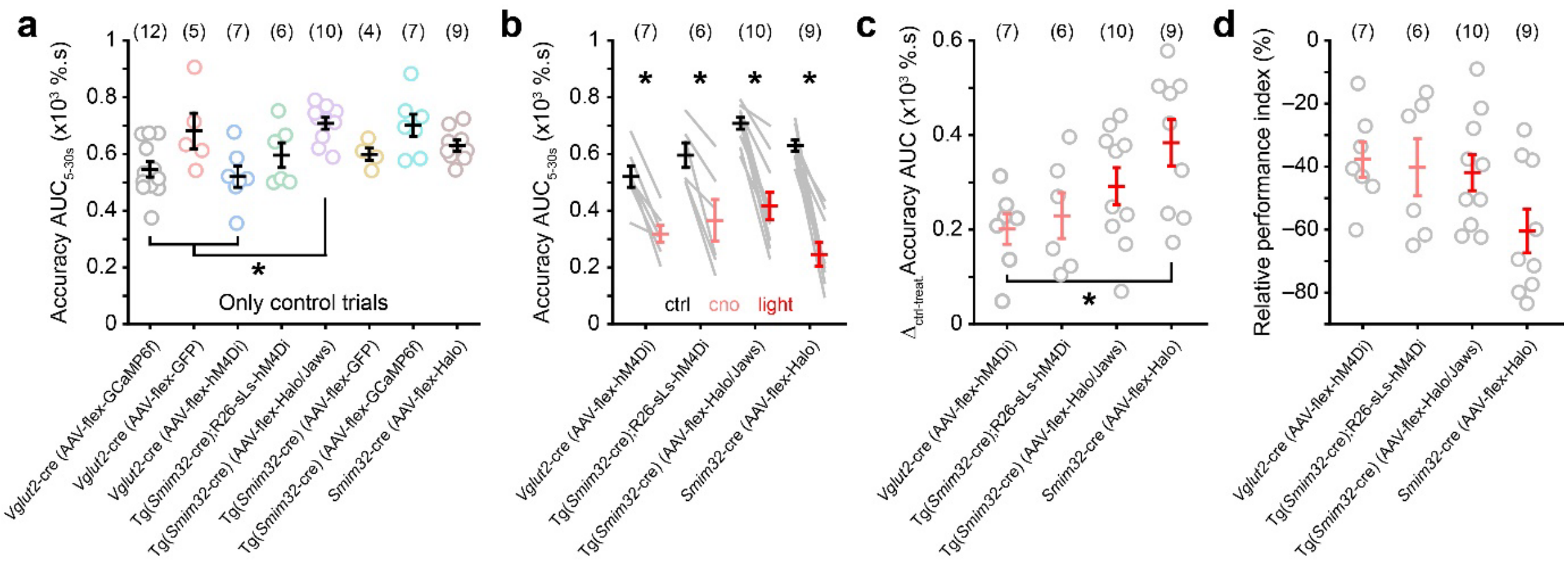

Supplementary Figure 5. Effects of CLA inhibition across optogenetic and chemogenetic manipulations in multiple Cre driver lines.
a, Quantification of overall OWM performance during the first 30 s, measured as the area under the curve (AUC), across the mouse cohorts shown in **Fig. 2**, **Fig. 5b-f**, **Supplementary fig. 1a**, and **Supplementary fig. 4c**. Only control conditions are included (Brown-Forsythe ANOVA $F_{(7,27.96)} = 4.57$ P =0.0017; post-hoc Dunnett's test $*P < 0.05$). **b,** Change in overall OWM performance following chemogenetic or optogenetic CLA inhibition for the different mouse cohorts shown in **Fig. 2**, **Fig. 5b-f**, **Supplementary fig. 1a**, and **Supplementary fig. 4c** (2WRM ANOVA treatment effect $F_{(3,28)} = 154.3$ $P < 10^{-10}$ & cohort x treatment interaction $F_{(3, 28)} = 154.3$ $P = 0.032$, post-hoc Bonferroni's test $*P < 0.00038$). **c,** Difference in OWM AUC corresponding to the data shown in **b** (Brown-Forsythe ANOVA $F_{(3,25.22)} = 3.59$ $P = 0.028$; post-hoc Dunnett's test $*P < 0.05$). **d,** Relative change in performance index (i.e. normalization to individual control conditions) for the data shown in **b** (Brown-Forsythe ANOVA $F_{(3,22.46)} = 2.44$ P =0.091). Genotype, manipulation type, and number of mice are indicated in each panel.

Lines 334-335 – "dexamethasone (4 mg/mL)" – What dose was delivered to the mice? The methods state a concentration. Same for carprofen (1 mg/mL) in line 341.

We thank the reviewer to have raised out this point. We have modified the methods to include the dosage for all drugs administered to mice during surgery and behavioral experimentations.

Line 376 – Were the same coordinates used for GRIN lens implantation as for viral injection but for the DV position? Please specify.

We thank the reviewer for pointing this out. Yes, the same coordinates were used for the GRIN lens implantation as for the viral injections. This information has now been added to the Methods section of the revised manuscript.

Line 400 – "a 0.2s tone, with a frequency ranging between 3 and 9 kHz depending on the animal". What was the rationale for presenting different tone frequencies to different animals?

We thank the reviewer for raising this point. To facilitate discrimination between the inter-trial interval and the inter-cue delay period, a brief 0.2-s auditory tone (3–9 kHz; frequency assigned individually to each mouse) was presented immediately before the sample cue on every trial. Behavioral sessions were conducted simultaneously across three to four rigs, and each mouse was consistently trained on the same setup using a fixed tone frequency. Although potential auditory contamination across adjacent rigs was minimized, assigning distinct tone frequencies ensured that each animal reliably associated its specific cue with the onset of the sample period. This clarification has now been incorporated into the revised manuscript.

Lines 403-407 and lines 450-451 – What criteria were used to determine the "higher count of licks" required to qualify as a 'Hit' (correct) trial? Was only one lick event during the response window required to classify the trial as the 'False Alarm' trial or was some other criterion used? Showing the motor behavior of different mice in different trial types in the supplement would help clarify this point.

We thank the reviewer for requesting clarification on trial classification criteria. During task training, mice learned to associate licking within the designated response window of non-match trials with reward delivery. To classify a trial as a "Hit," we applied a conservative relative criterion: a non-match trial was labeled as a Hit only when the number of licks during the response window exceeded the number of anticipatory licks in the pre-response window (defined as the interval immediately preceding the response period). This approach ensured that spontaneous or incidental licking did not confound behavioral classification. False Alarm trials were identified using the same relative comparison. Specifically, a match trial was classified as a False Alarm when licking during the response window exceeded licking in the pre-response window. This method accounts for individual differences in baseline licking behavior and minimizes spurious classifications arising from isolated or nonspecific lick events.

To further clarify this procedure, we have added motor-behavior data (lick-rate traces) from individual mice across all trial types (Hits, Misses, False Alarms, and Correct Rejections). These examples illustrate the temporal structure of licking behavior and its alignment with task contingencies. A detailed description of the classification criteria and thresholds has also been incorporated into the revised Methods section.

Lines 441-443 – Do mice withhold licks for 5 seconds after the tone following only 20 trials of training? Plots provided in the supplement showing the licking behavior of several example mice at different training stages would clarify the methods.

We thank the reviewer for this question. Mice do not reliably withhold licking for 5 seconds after the tone within the first 20 training trials. The behavioral protocol was intentionally structured in progressive stages (habituation, pre-training, and task training) to ensure gradual acquisition of cue associations, delayed responding, and appropriate lick timing. During habituation, mice were head-fixed and learned to lick for manually delivered water. In pre-training Stage 1, they then underwent a stepwise shaping procedure: in the first 20 trials, mice received an automatic water reward (2–3 µl) immediately after a 0.2 s tone (**Fig. Rebuttal 2a**). To begin uncoupling the tone from immediate reward, the delay between tone and water

delivery was gradually increased in blocks of 10 trials (**Fig. Rebuttal 2a**), teaching mice to associate licking with reward delivery rather than with the tone itself.

Once mice reliably licked for reward, we introduced the DNMS task using two odors with a fixed 5 s delay. In the first 20 non-match trials, water was again delivered automatically at the start of the response window to help mice learn the new cue–reward relationship. After this shaping period, water was delivered only if licking occurred during the response window, reinforcing the correct temporal structure (**Fig. Rebuttal 2b**). To ensure intentional responding, a "Hit" required that licking during the response window exceed licking during the pre-response period.

Across animals, approximately 150–200 trials were required for mice to learn the full task structure, suppress premature licking, and reliably initiate licking only at the appropriate time. Thus, withholding licking for the full 5-second delay does not emerge within the first 20 trials but develops gradually through structured training and reinforcement.

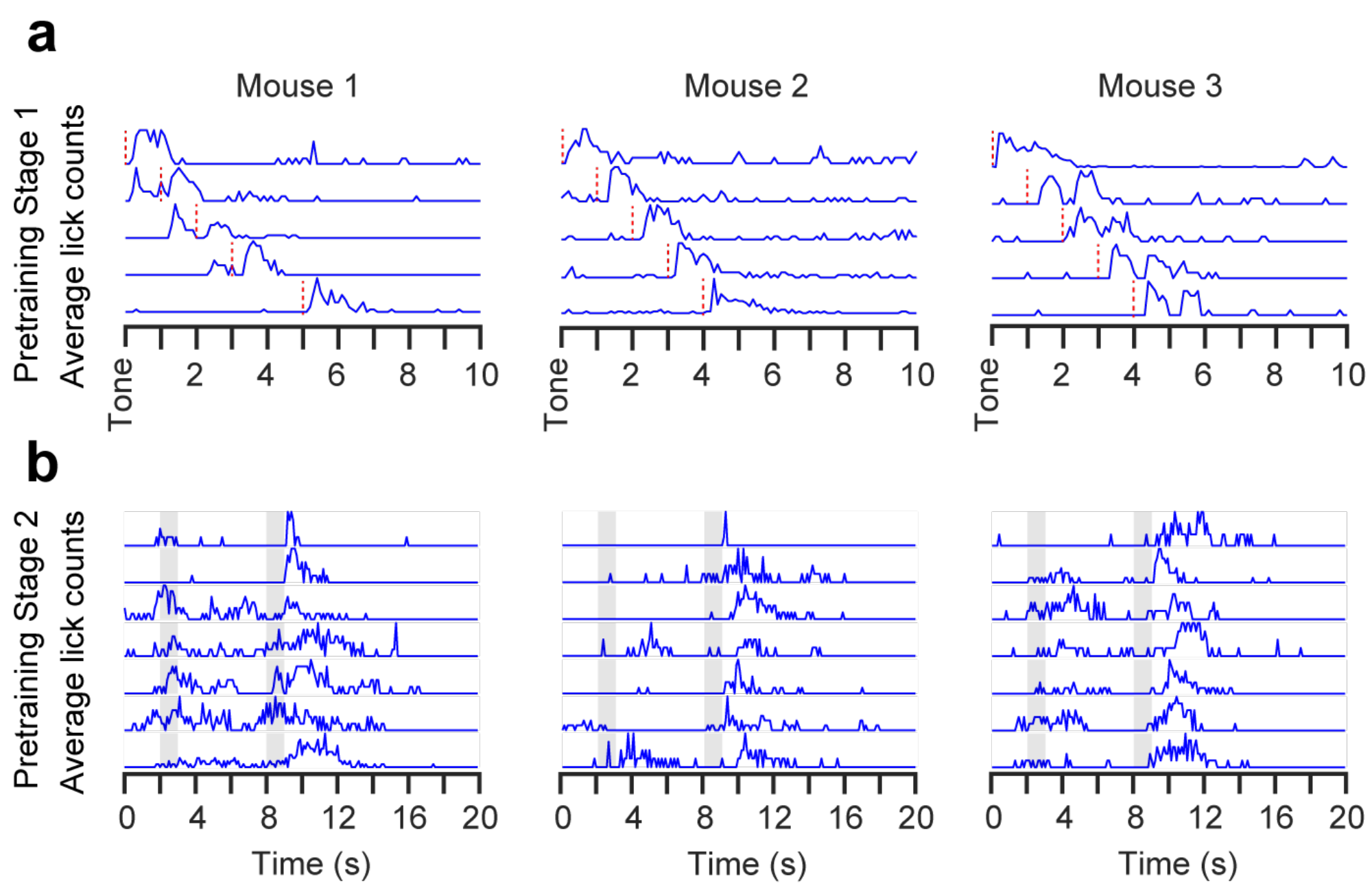

Figure Rebuttal 2. Progressive shaping of licking behavior during pre-training.
a, Average lick-rate traces across consecutive blocks of 10 trials during Stage 1 of pre-training in three representative mice. Water was initially delivered immediately following the auditory tone, and the delay between the tone and reward was progressively increased across trial blocks to decouple the cue from immediate reinforcement. Red dashed lines indicate the timing of water delivery for each block. **b,** Average lick-rate traces across consecutive 10-trial blocks during Stage 2. In the initial trials (first trace), free water was delivered at the onset of the response window to reinforce its temporal association with reward. In subsequent trials, automatic reward delivery was removed, and mice were required to lick within the response window to obtain water. Gray dashed lines indicate cue onset.

Lines 477-478 – The description of how the trial types were organized (i.e., delay times, match and non-match trials) in each behavioral session is not clear. Similarly, for lines 662-665.

Each daily session consisted of 100 trials, with matched and non-matched trials presented in equal proportions. Each delay condition was presented in approximately 14–16 trials. The full range of delays included 1 s (TSWM task only), 5 s, 10 s, 15 s, 20 s, 30 s, 40 s, and 60 s. Delay durations were pseudo-randomly varied in blocks of 2–4 trials and counterbalanced across

rewarded and non-rewarded conditions. This clarification has been added to the updated Methods section.

Line 604 – "DREADD in approximately 50% of the entire CLA population". Please provide quantification data to support this conclusion.

For some experiments, we used Tg(*Smim32*-cre)61^{Irod};Gt(ROSA)26^{Sortm1}(CAG-CHRM4*,-mCitrine)^{Ute}/J heterozygous mice to drive expression of the inhibitory DREADD in CLA neurons without requiring viral delivery. In our previous characterization of the *Smim32* transgenic lines [8], we demonstrated that *Smim32* is a reliable genetic marker for distinguishing claustral neurons from neighboring claustro-insular populations. *Smim32* is expressed in ~80% of *Nr4a2*$^{+}$ neurons and ~65% of *Gnb4*$^{+}$ neurons located within the anatomical boundaries attributed to the CLA. The Tg(*Smim32*-cre)61^{Irod} BAC transgenic line drives Cre expression in ~70% of all Smim32^{+} neurons. For simplicity, assuming that *Nr4a2* and *Gnb4* represent exclusive canonical claustral markers, the proportion of the total CLA population targeted in the Tg(*Smim32*-cre)61^{Irod} line can be estimated as:

- $0.7 \times 0.8 =$ **0.56** of the *Nr4a2*$^{+}$ population
- $0.7 \times 0.65 =$ **0.46** of the *Gnb4*$^{+}$ population

Thus, the statement that DREADD expression covers "approximately 50% of the entire CLA population" is consistent with these estimates, even though the exact percentage may vary given the known complexity of claustro-insular organization and the fact that *Nr4a2* and *Gnb4* are not exclusively selective for the CLA [10]. This clarification has been added to the updated Methods section.

Line 630 – 11 Vglut2-Cre mice? The total does not add up here.

We thank the reviewer for noting this potential discrepancy. We confirm that there is no inconsistency in the total number of mice reported. The 11 *Vglut2*-Cre mice in question were injected with the GCaMP8f calcium indicator; however, they were not included in the imaging experiments due to insufficient image quality through the GRIN lens. Instead, these animals were included exclusively in the behavioral control group lacking hM4Di expression (the "no hM4Di" group), thereby maintaining genetic background consistency across all experimental conditions.

Reviewer #3 (Remarks to the Author):

I co-reviewed this manuscript with one of the reviewers who provided the listed reports. This is part of the Nature Communications initiative to facilitate training in peer review and to provide appropriate recognition for Early Career Researchers who co-review manuscripts.

Thank you for your comments and for contributing to the peer review process.

References

1. Faig CA, Kim GHK, Do AD, Dworsky-Fried Z, Jackson J, Taylor AMW. Claustrum projections to the anterior cingulate modulate nociceptive and pain-associated behavior. *Curr Biol* **34**, 1987-1995 e1984 (2024).

2. Koga K, Kobayashi K, Tsuda M, Pickering AE, Furue H. Anterior cingulate cross-hemispheric inhibition via the claustrum resolves painful sensory conflict. *Commun Biol* **7**, 330 (2024).

3. Ntamati NR, Acuna MA, Nevian T. Pain-induced adaptations in the claustro-cingulate pathway. *Cell reports* **42**, 112506 (2023).

4. Goard MJ, Pho GN, Woodson J, Sur M. Distinct roles of visual, parietal, and frontal motor cortices in memory-guided sensorimotor decisions. *eLife* **5**, (2016).

5. Taxidis J*, et al.* Differential Emergence and Stability of Sensory and Temporal Representations in Context-Specific Hippocampal Sequences. *Neuron* **108**, 984-998 e989 (2020).

6. MacDonald CJ, Carrow S, Place R, Eichenbaum H. Distinct hippocampal time cell sequences represent odor memories in immobilized rats. *J Neurosci* **33**, 14607-14616 (2013).

7. Pnevmatikakis EA*, et al.* Simultaneous Denoising, Deconvolution, and Demixing of Calcium Imaging Data. *Neuron* **89**, 285-299 (2016).

8. Tuberosa J*, et al.* A toolbox for genetic targeting of the claustrum. *eLife*, (2024).

9. Chevee M, Finkel EA, Kim SJ, O'Connor DH, Brown SP. Neural activity in the mouse claustrum in a cross-modal sensory selection task. *Neuron* **110**, 486-501 e487 (2022).

10. Fodoulian L, Boillat M, Moulinier M, Carleton A, Rodriguez I. A spatial single-cell atlas of the claustro-insular region uncovers key regulators of neuronal identity and excitability. *Nature communications* **16**, 7830 (2025).

11. D'Esposito M, Postle BR. The cognitive neuroscience of working memory. *Annu Rev Psychol* **66**, 115-142 (2015).

12. Atlan G*, et al.* Claustrum neurons projecting to the anterior cingulate restrict engagement during sleep and behavior. *Nature communications* **15**, 5415 (2024).

Detailed response to reviewers' comments

We thank the reviewers for their time and are grateful for their insightful comments, which greatly helped improve the quality and clarity of our manuscript. In response, we performed additional analyses to address all concerns in detail, as outlined below.

For clarity, reviewers' comments are shown in black text and our responses in blue. In the revised manuscript, sections that have been substantially modified are highlighted in blue.

REVIEWER COMMENTS

Reviewer #1 (Remarks to the Author):

The authors have addressed my comments on the original manuscript to my satisfaction.

We thank the reviewer for their positive evaluation and are pleased that the revisions have addressed their concerns.

However, in one place, they have added new analysis which they claim shows that cue identity was maintained throughout the delay:

"Classifier performance was evaluated during two epochs: the early delay (ED; defined as the first second after the sample period) and the late delay (LD; the final second preceding target presentation). Classifier performance during both ED and LD periods was comparable and consistently exceeded chance levels (i.e., relative to shuffled data), indicating that cue identity was retained throughout the delay (Fig. 4f,g)."

This points out that they only tested the classifier on the first and last second of the delay. I believe this alone would not necessarily be enough to conclude that the cue representations were separable throughout the delay, as suggested by the PCA trajectories in Fig. 4e. Please also test the classifier on (held out) trials for arbitrary moments throughout the delay (including the middle, 1/4, and 3/4 of the way through the delay) and report the results alongside the early and late delay results.

We thank the reviewer for this helpful comment and agree that demonstrating classifier performance only during the early and late delay periods does not, by itself, establish that cue identity is maintained continuously throughout the delay. We appreciate the opportunity to strengthen this analysis.

As suggested, we expanded the decoding analysis to additional time points across the delay period. Specifically, in addition to the early- and late-delay epochs, classifier performance was evaluated in 1 s windows centered at one-quarter, mid-delay, and three-quarter delay. At each time point, the classifier was tested on held-out trials, allowing us to determine whether cue-related information remained decodable throughout the delay rather than only at its beginning and end. The revised analysis revealed that decoding performance remained consistently above shuffled controls at all sampled time points, including the one-quarter, mid-delay, and three-quarter-delay epochs. Thus, cue representations remained reliably separable throughout the delay period. These new analyses have been incorporated into the revised manuscript and are presented in the updated **Figure 4f**.

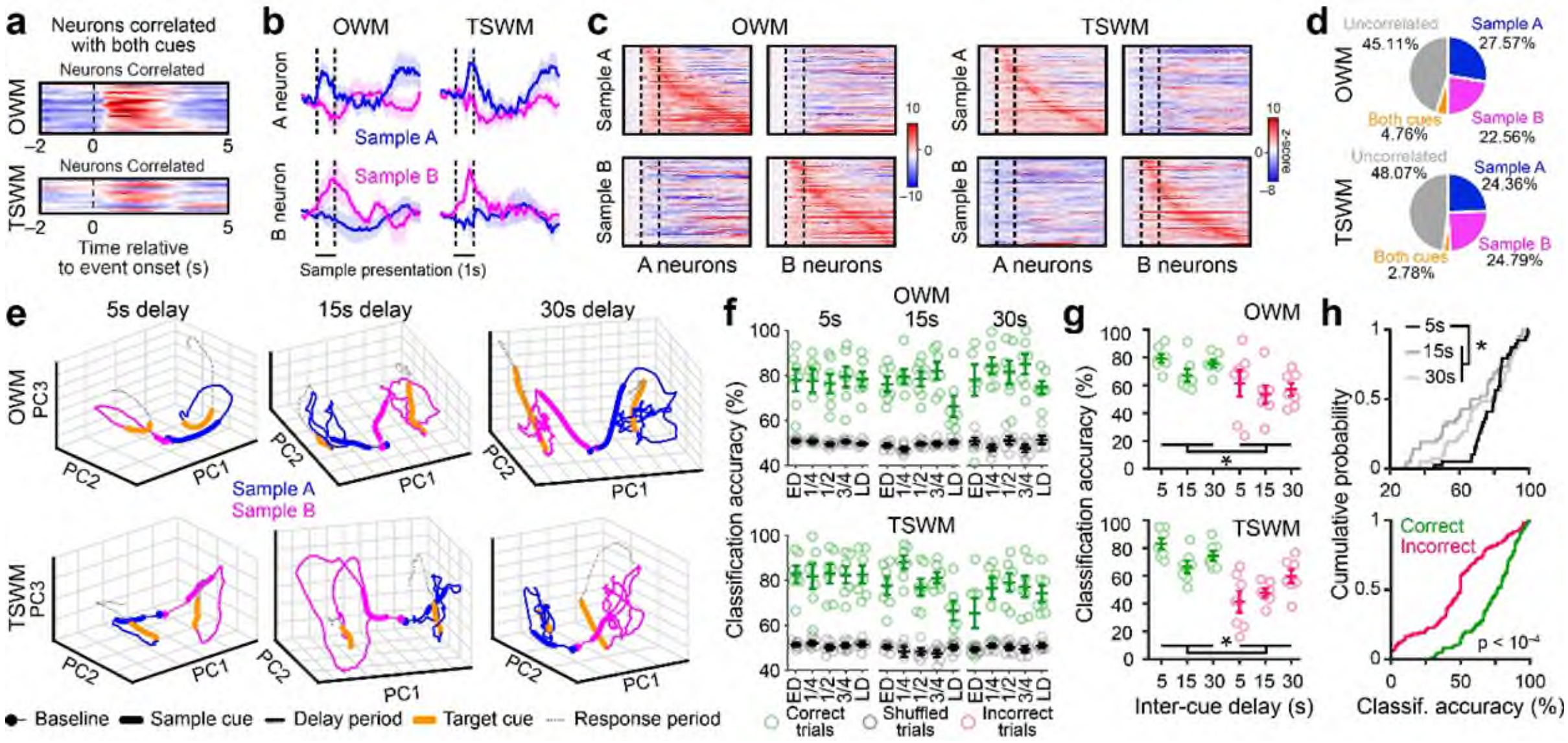

Figure 4. Claustrum ensembles maintain cue information during working memory.

a, Pseudo-colored heatmaps of neurons significantly correlated with both Sample A and B cues during OWM and TSWM tasks. **b,** Averaged calcium traces from four cue-selective stimulus neurons (mean ± SD). Each neuron responded to one cue but often showed complex delay responses for the non-preferred cue. **c,** Heatmaps of trial-averaged responses from all cue-selective stimulus neurons, sorted by peak latency for the preferred cue. Neuron order was preserved for non-preferred cue (n = 165, 150, 187, 191 neurons for samples A & B in OWM and TSWM tasks, respectively). **d**, Pie charts showing proportions of neurons correlated with both sample cues or only one, relative to the total recorded population in OWM and TSWM tasks. **e,** CLA population activity trajectories for the averaged correct trials with Sample A or B, plotted in 3D PCA space. **f,** Classification accuracy for decoding sample identity at regularly spaced time points across the delay period: early delay (ED; 1 s after sample onset), one-quarter delay (1/4), mid-delay (1/2), three-quarter delay (3/4), and late delay (LD; 1 s before target onset). Classification accuracy is shown for correct trials and shuffled controls in both tasks (*n* = 7 mice, RM ANOVA: shuffling effect $F_{(1,40)} = 350.9$ $P < 0.0001$ and $F_{(1,40)} = 353.2$ $P < 0.0001$, effect across time periods: $F_{(4,30)} = 1.4$ $P = 0.24$ and $F_{(4,30)} = 1.396$ $P = 0.26$, for OWM and TSWM, respectively). **g,** Classification accuracy for decoding sample identity during late delay periods in correct and incorrect trials for both tasks (*n* = 7 mice, correct vs. incorrect trials: RM ANOVA $F_{(1,6)} = 15.48$, $P = 0.0077$ and $F_{(1,6)} = 27.71$, $P = 0.0019$ for OWM and TSWM tasks, respectively). **h,** Cumulative probability distributions of sample cue classification accuracy. *Top graph*: correct trials for different delays (*n* = 42 animal/session/task triplets, Kolmogorov-Smirnov test $P = 3.10^{-4}$, $P = 0.009$, and $P = 0.29$ for 5s vs. 15s. 5s vs. 30s, and 15s vs. 30s, respectively). *Bottom graph*: comparison between correct and incorrect trials (*n* = 126 & 124 animal/delay/session/task quadruplets for correct & incorrect trials, respectively). Data are presented as mean ± SEM. See **supplementary Table 1** for detailed statistics.

Reviewer #2 (Remarks to the Author):

The authors were very responsive to the reviewer's comments, presenting additional experiments, new analyses and a substantive revision of the text and figures, thus addressing the major concerns raised in the prior reviews. However, the clarifications in the methods related to how behavioral trials are classified raises an additional point.

The authors now clarify that trial outcomes are defined based on the number of licks in a fixed response window (2.5s) relative to the number of licks in a variable pre-response period which can extend from ~5s to ~60s (p. 25). The period over which to accumulate licks to compare with the licks detected in the fixed response window varies across the delay lengths (short to long), raising the possibility that trials are classified differently for shorter and longer delays. For example, in Supplementary Figure 1a, it seems that fewer licks during the fixed response window are allowed to call a trial a CR trial for shorter delays than for longer delays (compare amplitudes of top pink and bottom pink traces in OWM and TSWM plots). Similarly, more licks may be required to call a long delay trial a FA trial relative to the number for short delay trials, and trials may fail to be classified as hits not due to impaired memory per se, but because mice are required to generate a larger number of licks within the 2.5s response window for long trial types (with longer pre-response periods) than for short trial types. Supplementary Figure 1b also suggests such effects: at short trial durations, hits and CRs are about the same percentage as are misses and FAs; however, as trial duration gets longer, the proportions change with fewer hits and more CRs as well as more misses and fewer FAs. Determining whether and to what extent these differences in pre-response period lengths relative to the fixed response window contribute to the classification of trial outcomes is central to many points in the paper. Does using a fixed 5 s pre-response period (like the period just prior to the response window) affect the trial classification (hit, FA, CA, miss)?

Further, in addition to plotting normalized average lick traces, giving the absolute values and ranges (i.e. a measure of central tendency - median lick count - and variability - IQR) for the number of licks in the response and pre-response windows across delay conditions and trial-outcome types further clarify the results.

We thank the reviewer for recognizing the substantial revisions and additional analyses included in the revised manuscript. We also appreciate this insightful comment regarding trial classification, which prompted us to examine more directly whether the use of a variable pre-response window could have influenced the assignment of behavioral outcomes across delay durations.

We agree that, because the original pre-response window increased with delay length, longer-delay trials provided a greater opportunity for sample-evoked or anticipatory licking to accumulate. In principle, this could result in different classification criteria across delay durations, making the criterion more stringent for longer delays because the increase in licking during the fixed 2.5 s response window would need to exceed a baseline measured over a progressively longer period. We had anticipated this potential trade-off when designing the task and therefore evaluated it explicitly (see analyses below). However, our primary concern was that mice might exhibit increasing levels of spontaneous or compulsive licking as the delay lengthened. By keeping the pre-response window proportional to the delay, we normalized the baseline to the amount of time available for such non-specific licking, thereby reducing the

likelihood that spontaneous licking at longer delays would be misclassified as a Hit or False Alarm.

To address this concern, we performed a post hoc trial reclassification using a fixed-duration pre-response window. Specifically, we constructed a 5 s baseline consisting of the first 2.5 s following sample-cue onset and the final 2.5 s immediately preceding the response window. This window was selected because it captures the two periods during which non-response licking is most likely to occur: sample-evoked licking immediately after cue presentation and anticipatory licking just before the response window. Licking during this fixed baseline was then compared with licking during the 2.5 s response window using the same classification criteria as in the original analysis. Importantly, applying this fixed-baseline classification produced behavioral outcome distributions that were highly similar to those obtained with the original variable pre-response window (**Supplementary Fig. 1c,d**). The delay-dependent changes in Hit, Miss, Correct Rejection, and False Alarm rates were preserved, demonstrating that the behavioral effects were not an artifact of the increasing baseline duration but instead reflected genuine changes in task performance with increasing delay.

In addition, as suggested by the reviewer, we now report the absolute lick counts during both the pre-response and response windows across delay conditions and behavioral outcome categories using individual mouse-session data (**Supplementary Fig. 1b**). The resulting lick-count distributions are broadly comparable across delay durations and provide no evidence that longer-delay trials required disproportionately greater response-window licking to be classified as Hits or False Alarms.

We have also revised the **Methods** section to clarify the trial-classification procedure used during behavioral testing.

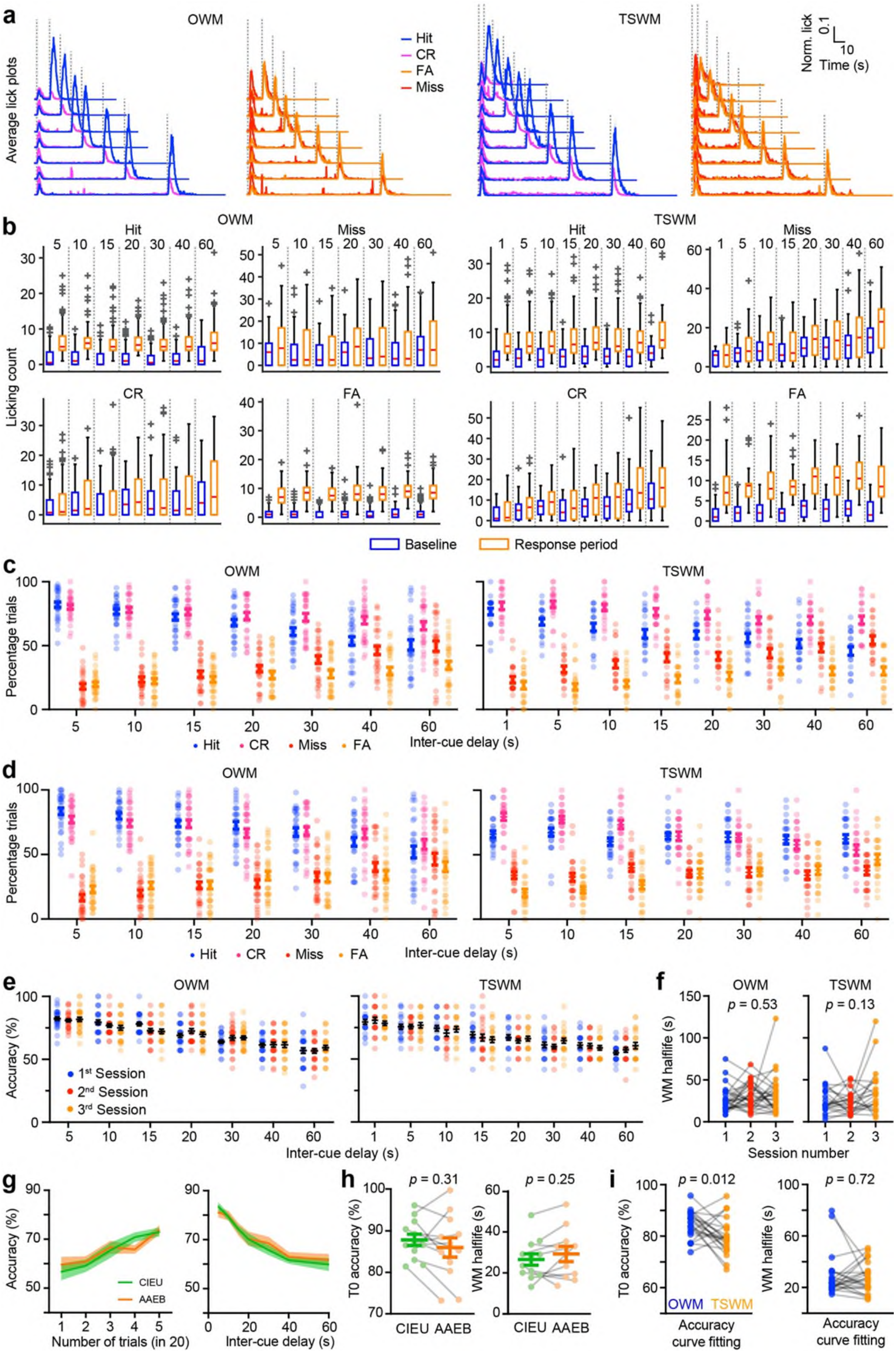
a
OWM
TSWM
Average lick plots
Hit
CR
FA
Miss
Norm. lick
0.1
10
Time (s)
b
OWM
TSWM
Hit
Miss
CR
FA
Licking count
Baseline
Response period
c
OWM
TSWM
Percentage trials
Hit
CR
Miss
FA
Inter-cue delay (s)
d
OWM
TSWM
Percentage trials
Hit
CR
Miss
FA
Inter-cue delay (s)
e
OWM
TSWM
Accuracy (%)
1st Session
2nd Session
3rd Session
Inter-cue delay (s)
f
OWM
TSWM
p = 0.53
p = 0.13
WM halflife (s)
Session number
g
Accuracy (%)
CIEU
AAEB
Number of trials (in 20)
Inter-cue delay (s)
h
p = 0.31
p = 0.25
T0 accuracy (%)
WM halflife (s)
CIEU
AAEB
i
p = 0.012
p = 0.72
T0 accuracy (%)
WM halflife (s)
OWM
TSWM
Accuracy curve fitting

Supplementary Figure 1. Stability of WM task performance across conditions.
a, PSTHs of normalized lick onset events for each behavioral outcome (Hits, Misses, Correct Rejections, and False Alarms) across inter-cue delays in OWM and TSWM tasks. Grey dotted lines mark the onset of sample and target cues. **b,** Lick counts during the baseline and response windows periods for each behavioral outcome (Hits, Misses, Correct Rejections, and False Alarms). Data are shown as box plots representing individual mouse-session measurements, with the median, interquartile range and outliers (gray crosses). **c,** Proportions of behavioral outcomes (Hits, Misses, Correct Rejections, and False Alarms) across inter-cue delays in the OWM and TSWM tasks, using the trial-classification criterion employed during behavioral testing, in which the pre-response baseline spanned the entire delay period. **d,** Proportions of behavioral outcomes across inter-cue delays following post hoc reclassification using a fixed 5 s pre-response baseline composed of the first 2.5 s after sample-cue onset and the final 2.5 s immediately preceding the response window. **e,** Session-wise accuracy comparison across delays in OWM and TSWM tasks. **f,** Stability of WM performance assessed via session-wise half-life values (RM one-way ANOVA: OWM, $P = 0.53$; TSWM, $P = 0.13$). **g,** *Left*: Early learning curves for two odor pairs (CIEU and AAEB) using a fixed 5s inter-cue delay (2WRM ANOVA: $F_{(1,22)} = 0.03$, $P = 0.86$; $n = 12$). *Right*: WM performance across inter-cue delays for the same odor pairs (2WRM ANOVA: $F_{(1,22)} = 0.07$, $P = 0.80$). **h,** Comparison of WM performance amplitude at 0 delay and half-life for the two odor pairs (paired t-test). **i,** Comparison of WM accuracy at 0s delay and the half-life value (parameters derived from exponential curve fitting) for the two odor pairs (paired *t*-test). Data are presented as mean ± SEM. See **Supplementary Table 1** for detailed statistics.

Minor comments:

Lines 435-436, 856-858: What is the difference between Tg(Smim32-Cre)61Irod heterozygotes and Smim32-Cre knock-in mice? Are the latter homozygous and the former hemizygous for the transgene?

The *Smim32*-IRES-Cre knock-in mouse line was generated by inserting an IRES-Cre recombinase cassette immediately downstream of the endogenous *Smim32* stop codon, thereby preserving endogenous Smim32 expression while driving Cre recombinase from the native *Smim32* promoter.

The Tg(*Smim32*-Cre)61Irod transgenic line was generated by introducing a Cre-expressing transgene under the control of the *Smim32* regulatory elements using a bacterial artificial chromosome (BAC). The transgene was derived by modifying BAC RP23-125G21 from the RPCI-23 BAC library, which spans the genomic region from the 3′ intergenic region upstream of *Smad5* to the second intron of *Trpc7*, encompassing the entire *Smim32* locus. Using BAC recombineering, the Cre–rabbit β-globin polyA–frt–PuroR–ZeoR–frt cassette was inserted in place of the *Smim32* start codon. The modified BAC was then injected into the pronuclei of C57BL/6J × DBA/2J F2 zygotes to generate transgenic founders. Four founders were obtained, of which two independent lines were established based on their Cre expression patterns, including Tg(Smim32-Cre)61Irod.

Both mouse lines are maintained as hemizygotes. A detailed description of their generation, characterization, and expression specificity is provided in Tuberosa et al., *A toolbox for genetic targeting of the claustrum*, eLife (2025), 14:e99168. https://doi.org/10.7554/eLife.99168.1

Some minor typos: Line 462: “0.9jM NaCl” Line 599-600: sensitivity Index Line 848: one Rosa-ISL-hM4Di mouse

We thank the reviewer for pointing out these typo errors. They have now been corrected in the revised manuscript.
